# The molecular signature and prognosis of glioma with preoperative intratumoral hemorrhage: a retrospective cohort analysis

**DOI:** 10.1186/s12883-024-03703-2

**Published:** 2024-06-14

**Authors:** Yixin Shi, Xiaoman Kang, Yulu Ge, Yaning Cao, Yilin Li, Xiaopeng Guo, Wenlin Chen, Siying Guo, Yaning Wang, Delin Liu, Yuekun Wang, Hao Xing, Yu Xia, Junlin Li, Jiaming Wu, Tingyu Liang, Hai Wang, Qianshu Liu, Shanmu Jin, Tian Qu, Huanzhang Li, Tianrui Yang, Kun Zhang, Feng Feng, Yu Wang, Hui You, Wenbin Ma

**Affiliations:** 1 Department of Neurosurgery, Center for Malignant Brain Tumors, Peking Union Medical College Hospital, National Glioma MDT Alliance, Chinese Academy of Medical Sciences and Peking Union Medical College, Beijing, 100730 China; 2 China Anti-Cancer Association Specialty Committee of Glioma, Beijing, 100730 China; 3 Eight-year Medical Doctor Program, Chinese Academy of Medical Sciences and Peking Union Medical College, Beijing, 100730 China; 4 ‘4+4’ Medical Doctor Program, Chinese Academy of Medical Sciences and Peking Union Medical College, Beijing, 100730 China; 5 Department of Radiology, Peking Union Medical College Hospital, Chinese Academy of Medical Sciences, Peking Union Medical College, Beijing, 100730 China

**Keywords:** Glioma, Intracranial hemorrhage, Magnetic resonance imaging, Biomarkers, tumor, Central Nervous System Neoplasms

## Abstract

**Background:**

Intratumoral hemorrhage, though less common, could be the first clinical manifestation of glioma and is detectable via MRI; however, its exact impacts on patient outcomes remain unclear and controversial. The 2021 WHO CNS 5 classification emphasised genetic and molecular features, initiating the necessity to establish the correlation between hemorrhage and molecular alterations. This study aims to determine the prevalence of intratumoral hemorrhage in glioma subtypes and identify associated molecular and clinical characteristics to improve patient management.

**Methods:**

Integrated clinical data and imaging studies of patients who underwent surgery at the Department of Neurosurgery at Peking Union Medical College Hospital from January 2011 to January 2022 with pathological confirmation of glioma were retrospectively reviewed. Patients were divided into hemorrhage and non-hemorrhage groups based on preoperative magnetic resonance imaging. A comparison and survival analysis were conducted with the two groups. In terms of subgroup analysis, we classified patients into astrocytoma, IDH-mutant; oligodendroglioma, IDH-mutant, 1p/19q-codeleted; glioblastoma, IDH-wildtype; pediatric-type gliomas; or circumscribed glioma using integrated histological and molecular characteristics, according to WHO CNS 5 classifications.

**Results:**

457 patients were enrolled in the analysis, including 67 (14.7%) patients with intratumoral hemorrhage. The hemorrhage group was significantly older and had worse preoperative Karnofsky performance scores. The hemorrhage group had a higher occurrence of neurological impairment and a higher Ki-67 index. Molecular analysis indicated that CDKN2B, KMT5B, and PIK3CA alteration occurred more in the hemorrhage group (CDKN2B, 84.4% vs. 62.2%, *p* = 0.029; KMT5B, 25.0% vs. 8.9%, *p* = 0.029; and PIK3CA, 81.3% vs. 58.5%, *p* = 0.029). Survival analysis showed significantly worse prognoses for the hemorrhage group (hemorrhage 18.4 months vs. non-hemorrhage 39.1 months, *p* = 0.01). In subgroup analysis, the multivariate analysis showed that intra-tumoral hemorrhage is an independent risk factor only in glioblastoma, IDH-wildtype (162 cases of 457 overall, HR = 1.72, *p* = 0.026), but not in other types of gliomas. The molecular alteration of CDK6 (hemorrhage group *p* = 0.004, non-hemorrhage group *p* < 0.001), EGFR (hemorrhage group *p* = 0.003, non-hemorrhage group *p* = 0.001), and FGFR2 (hemorrhage group *p* = 0.007, non-hemorrhage group *p* = 0.001) was associated with shorter overall survival time in both hemorrhage and non-hemorrhage groups.

**Conclusions:**

Glioma patients with preoperative intratumoral hemorrhage had unfavorable prognoses compared to their nonhemorrhage counterparts. CDKN2B, KMT5B, and PIK3CA alterations were associated with an increased occurrence of intratumoral hemorrhage, which might be future targets for further investigation of intratumoral hemorrhage.

**Supplementary Information:**

The online version contains supplementary material available at 10.1186/s12883-024-03703-2.

## Background

Gliomas are a heterogeneous group of primary brain tumors, with an annual incidence of six cases per 100,000 worldwide [1]. Common symptoms associated with adult diffuse gliomas are epilepsy, neurocognitive alterations, and signs of elevated intracranial pressure [1], and current standard treatments include temozolomide chemotherapy, radiotherapy, and surgery [1]. However, even with ongoing improvements in treatment, the prognosis for some glioma subtypes remains quite dismal, with a median survival of less than two years for glioblastoma, the most common primary malignancy of the central nervous system [2]. Magnetic resonance imaging (MRI) is a non-invasive method to visualize intracranial lesions and can detect imaging-based prognostic factors early in disease management [3]. Intratumoral hemorrhage, diagnosed by preoperative MRI, is a feature occasionally seen in brain tumor patients [3]. Although intratumoral hemorrhage occurs less frequently than other common symptoms mentioned above for gliomas (2.5% for glioblastomas [4]), it can be the first sign of glioma and may lead to unfavorable outcomes [5, 6].

However, the exact impact of tumor-associated hemorrhages on patient outcomes remains unclear, and this is incredibly disappointing in light of the newly released 2021 World Health Organization classifications (WHO CNS 5 classification) [7]. These diagnostic criteria emphasize genetic and molecular features, but ignore hemorrhages. This update has also changed the classification for specific tumor types. For example, IDH-wildtype diffuse astrocytoma with WHO grades II to III in the previous version of classification could be upgraded to glioblastoma if it has any of the molecular alterations of EGFR amplification, chromosome + 7/-10, or TERT promoter mutation in the latest classification [7]. As a result, the incidence of hemorrhage in different glioma subtypes might also now be diagnostically reclassified accordingly. Therefore, an updated investigation of glioma-related hemorrhage is urgently needed and may provide valuable insights into the stratified clinical management of patients with distinct molecular and clinical characteristics.

Aside from controversies over the clinical implications of intratumoral hemorrhage, the tumor types associated with higher hemorrhagic risk are also unclear. Previous studies have adopted the older 2016 WHO classification [4] or analyzed heterogeneous patient populations, including primary and metastatic brain tumors [8, 9]. Although there is evidence that in brain metastasis patients, intratumoral hemorrhage before surgery is an independent predictor for worse survival time [10], studies with large homogeneous cohorts are lacking for intratumoral hemorrhage associated with glioma. Understanding how hemorrhage affects outcomes in glioma patients is particularly crucial because intratumoral hemorrhage can be the first presentation for specific subtypes of gliomas, such as glioblastoma (GBM) [6].

To date, only a few studies focused on glioma-associated intratumoral hemorrhage, and most of them employed the old 2016 WHO classifications with heterogeneous patient populations [4, 9, 11, 12]. Therefore, this study aims to establish the prevalence of intratumoral hemorrhage in glioma subtypes based on the new WHO classifications and identify distinctive molecular and clinical characteristics among hemorrhagic patients compared to non-hemorrhage patients. With a large cohort of only primary brain tumors, this study offers reliable insights into potential hemorrhage-associated molecular markers as predictors of survival, as well as guidance for clinical management.

## Methods

### Study cohort

This study is a retrospective cohort study and is reported following the STROBE guidelines. A retrospective review of integrated clinical data and imaging studies of patients who underwent surgery at the Department of Neurosurgery at Peking Union Medical College Hospital from January 2011 to January 2022 was carried out with pathological confirmation of glioma. Patients with comprehensive clinical and imaging data were finally enrolled for analysis and divided into hemorrhage and non-hemorrhage groups based on their MRI presentations. This study was approved by the hospital’s Institutional Ethics Review Board (S-424).

### Clinical data acquisition

Clinical and radiological information was collected retrospectively from patients’ medical records and examinations. This information included patient gender, age at diagnosis, body mass index (BMI), preoperative Karnofsky performance score (KPS), and clinical symptoms. Histopathological data were obtained from reports from the Department of Pathology of Peking Union Medical College Hospital, and these data included the Ki-67 index and histological grade of the tumors. Additionally, survival status and survival time were acquired via regular follow-up. Overall survival (OS) was defined as the time from the surgery date to the patient’s death or final follow-up (treated as censored values).

### Imaging assessment and definition of intratumoral hemorrhage

The radiological features of the patients were collected from their preoperative magnetic resonance imaging (MRI) results using 3.0-Tesla equipment (Discovery MR750, GE, US) before treatment. Two authors (X.K. and Y.G.) who were blind to patient identification, independently assessed the imaging data for each patient and determined whether there was intracerebral hemorrhage within a tumor. A third author (Y.S.) assessed the imaging data to break the tie if a disagreement occurred. An MRI diagnosis of intratumoral hemorrhage was based on precontrast T1-weighted and T2-weighted imaging, as defined by standard MRI criteria with hyperintensity on T1-weighted images and hyper- or hypointensity on T2-weighted images, or purely low signal intensity on both T1 and T2-weighted images [3]. Patients with indeterminate hemorrhage underwent further CT imaging screening if necessary.

### Molecular analysis and integrated classification

We analyzed molecular alterations of each patient using second-generation sequencing, polymerase chain reaction-based assay, and fluorescence in situ hybridization methods. Sixty molecular markers were screened, including EGFR, TERT, CDKN2A/B, MYB, and MYBL1, which have been indicated as significant in the development mechanism and prognosis prediction of gliomas in previous studies. All patients were then re-classified into astrocytoma, IDH-mutant; oligodendroglioma, IDH-mutant, 1p/19q-codeleted; glioblastoma, IDH-wildtype; pediatric-type gliomas; or circumscribed glioma using integrated histological and molecular characteristics by an experienced pathologist, according to WHO CNS 5 classifications. We used the new classification to conduct subgroup analysis in the subsequent analysis.

### Statistical analysis

Normally distributed random variables were expressed as means ± standard deviations (SDs), and differences between groups for such variables were determined by Student’s t-test. Non-normally distributed variables were expressed as medians (first quartile, third quartile), and comparisons of differences in these variables between groups were conducted by the Kruskal-Wallis H test. Comparisons of categorical variables were performed using the chi-squared test. Additionally, a waterfall heat map was utilized to visualize the molecular signatures of the hemorrhage and non-hemorrhage groups. Survival analysis was performed using the Kaplan-Meier method and Log-Rank test, and the results are presented using Kaplan-Meier curves. Univariate Cox and multivariate Cox regression were utilized for prognostic risk factor screening in different glioma groups, including glioblastoma, astrocytoma and oligodendroglioma. The predictive value of baseline characteristics for intratumoral hemorrhage is investigated utilizing logistic regression. A two-sided *P* < 0.05 was considered to indicate statistically significant test results for all tests. All statistical and graphical analysis was performed using SPSS (version 26.0, IBM, USA) statistical software and R Studio (PBC & Certified B Corp.®, USA) software, respectively.

## Results

### Baseline characteristics of glioma patients with and without intratumoral hemorrhage

A total of 457 glioma patients with comprehensive imaging data were retrospectively enrolled in this study, including 67 (14.7%) patients with intratumoral hemorrhage. Among the enrolled patients, 263 were males, and 58.2% of the hemorrhage patients were male. The median age of all patients was 49.0, and patients with hemorrhage were older than non-hemorrhage patients (53.0 vs. 48.0, *p* = 0.012). The median preoperative KPS for patients in the hemorrhage group was 80, worse than those in the non-hemorrhage group, with a median KPS of 90 (*p* = 0.015). In terms of clinical presentations, neurological impairment was the most common symptom in both groups but tended to occur more in the hemorrhage group (74.6% vs. 53.9%, *p* = 0.002). However, intracranial hypertension was more common in the non-hemorrhage group (28.4% vs. 45.5%, *p* = 0.013). A significant difference in tumor growth was also observed between the two groups, with higher Ki-67 in the hemorrhage group (20% vs. 10%, *p* = 0.001). However, there was no difference in histological grade or WHO CNS 5 classification between the two groups. Finally, glioblastoma, IDH-wildtype, was the most common glioma subtype in the hemorrhage group (33/67, 57.9%). The detailed information is shown in Table 1.

**Table 1:** Baseline characteristics of glioma patients with and without intratumoral hemorrhage

	All patients (*n* = 457)	Hemorrhage Group (*n* = 67)	Non-hemorrhage Group (*n* = 390)	*P* value
**Gender**				1.000
Male	263, 57.5%	39, 58.2%	224, 57.4%	
Female	194, 42.5%	28, 41.8%	166, 42.6%	
**Mean Age, year** (median [IQR])	49.0 [36.0, 59.0]	53.0 [42.5, 62.5]	48.0 [35.2, 58.0]	**0.012**
**Age, year**				**0.013**
< 60	345, 75.5%	42, 62.7%	303, 77.7%	
≥ 60	112, 24.5%	25, 37.3%	87, 22.3%	
**Mean BMI, kg/m** ^**2**^	24.1 ± 3.5	24.0 ± 3.5	24.2 ± 3.5	0.666
**BMI, kg/m** ^**2**^				0.278
< 24	221, 48.4%	37, 55.2%	184, 47.2%	
≥ 24	236, 51.6%	30, 44.8%	206, 52.8%	
**Median Preoperative KPS** (median [IQR])	90.0 [80.0, 100.0]	80.0 [80.0, 95.0]	90.0 [80.0, 100.0]	**0.015**
**Preoperative KPS**				1.000
< 70	41, 9.0%	6, 9.0%	35, 9.0%	
≥ 70	416, 91%	61, 91.0%	355, 91.0%	
**Clinical symptoms**				
Intracranial hypertension	196, 43.0%	19, 28.4%	177, 45.5%	**0.013**
Neurologic impairment	259, 56.9%	50, 74.6%	209, 53.9%	**0.002**
Epilepsy	139, 30.8%	15, 22.7%	124, 32.2%	0.162
**Ki-67, %** (median [IQR])	10.0 [3.0, 30.0]	20.0 [5.0, 50.0]	10.0 [2.0, 30.0]	**0.001**
**WHO5 CNS classification**				0.297
Astrocytoma, IDH-mutant	63, 18.9%	11, 19.3%	52, 18.8%	
Oligodendroglioma, IDH-mutant and 1p/19q-codeleted	86, 25.8%	11, 19.3%	75, 27.2%	
Glioblastoma, IDH-wildtype	162, 48.6%	33, 57.9%	129, 46.7%	
Pediatric-type diffuse high-grade glioma	7, 2.1%	0, 0.0%	7, 2.5%	
Pediatric-type diffuse low-grade glioma	7, 2.1%	2, 3.5%	5, 1.8%	
Circumscribed glioma	8, 2.4%	0, 0.0%	8, 2.9%	

### Survival and subgroup analysis of patients with and without intratumoral hemorrhage

**Fig. 1:**
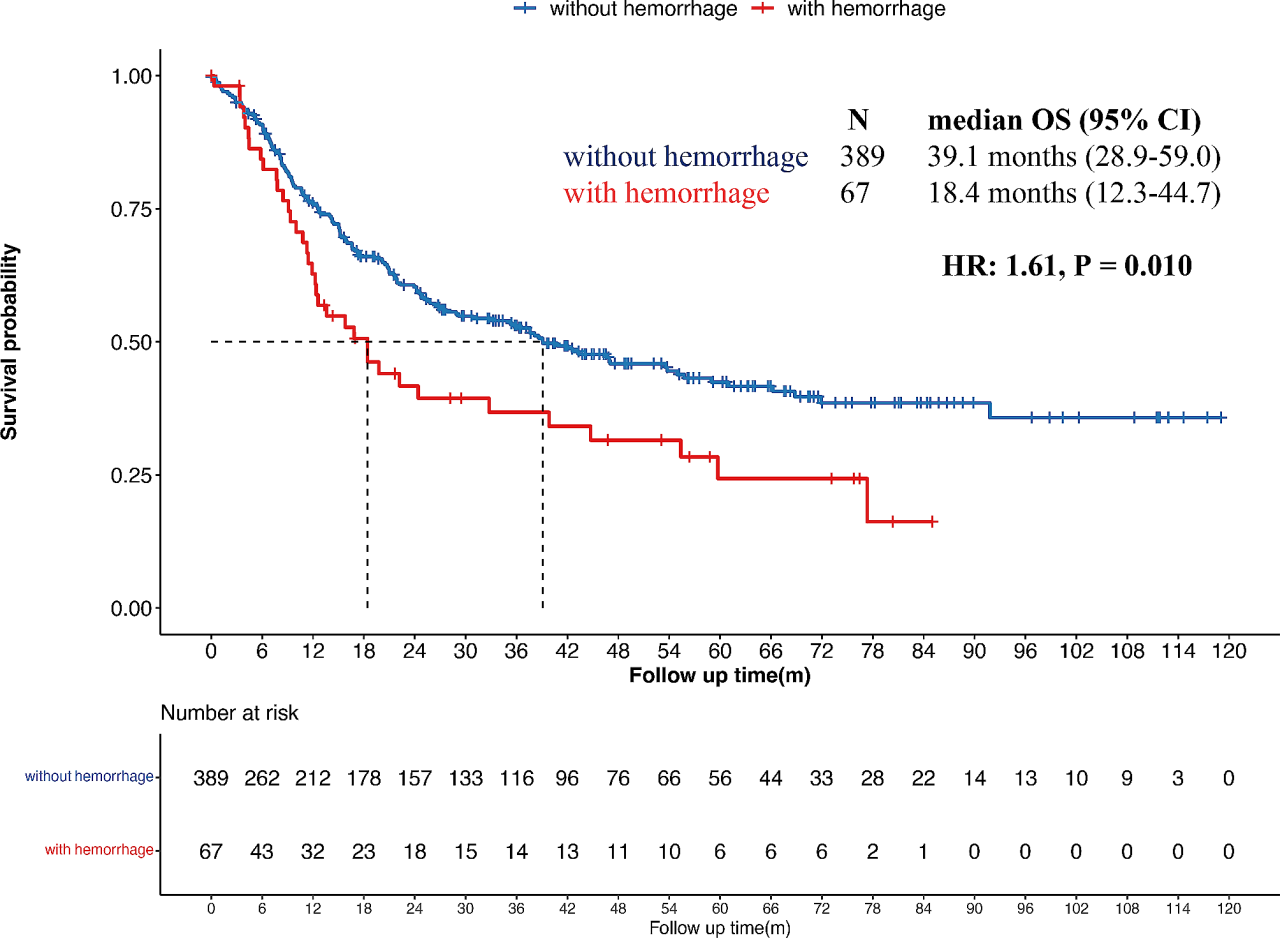
Survival analysis of patients with and without hemorrhage in the whole group. The median overall survival (mOS) times of glioma patients with intratumoral hemorrhage and without hemorrhage were 18.4 months and 39.1 months, respectively (*P* = 0.01)

We explored the survival of patients with and without intratumoral hemorrhage and found that the median overall survival (mOS) of patients in the two groups was 18.4 months and 39.1 months, respectively, and that patients with intratumoral hemorrhage had a significantly worse prognosis compared to those in the non-hemorrhage group (*p* = 0.010, Fig. 1). Further subgroup survival analysis showed that the baseline factors considerably influencing the prognosis of patients without hemorrhage were less prevalent in the hemorrhage group, including age, KPS score and pathology subtypes (Figure S1). Patients older than 60, with KPS less than 70, and with glioblastoma tended to have shorter survival times (*p* < 0.001, *p* < 0.001, and *p* = 0.002, respectively) in the non-hemorrhage group. However, in the hemorrhage group, age and WHO CNS 5 classification displayed no statistical significance in patient prognosis (*p* = 0.728 and *p* = 0.125, respectively), but KPS < 70 led to a significantly poorer prognosis (*p* = 0.029).

**Fig. 2:**
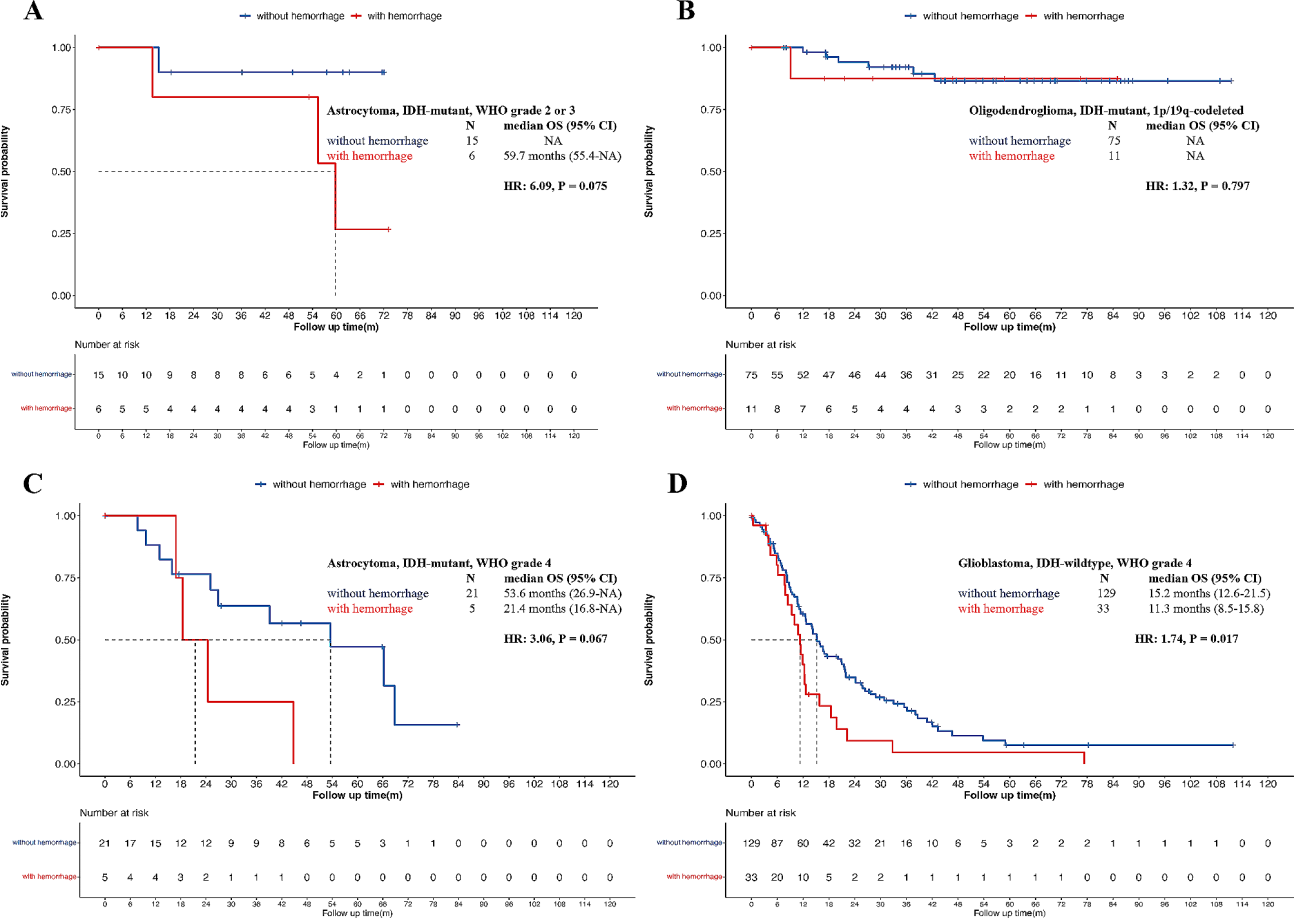
Survival analysis of patients with and without hemorrhage in pathological subgroups. This figure depicts that patients with and without intratumoral hemorrhage showed no significant difference in astrocytoma, IDH mutant, WHO grade and 3 (Fig. 2A, *p* = 0.075), oligodendroglioma (Fig. 2B, *p* = 0.797) and astrocytoma, IDH mutant, WHO grade 4 (Fig. 2C, *p* = 0.067). Within the group of glioblastoma, patients with intratumoral hemorrhage have significantly shorter survival time than those without hemorrhage (Fig. 2D, *p* = 0.017)

Furthermore, the subgroup analysis in most tumor types did not show significant differences between hemorrhage and non-hemorrhage groups among astrocytoma, grade 2 or 3 and grade 4, and oligodendroglioma (*p* = 0.075, *p =* 0.067 and *p* = 0.797, respectively). However, among glioblastoma patients, those with hemorrhage had significantly lower mOS (11.3 months vs. 15.2 months, *p* = 0.017, Fig. 2).

### Univariate and multivariate analysis of clinical characteristics related to survival

**Fig. 3:**
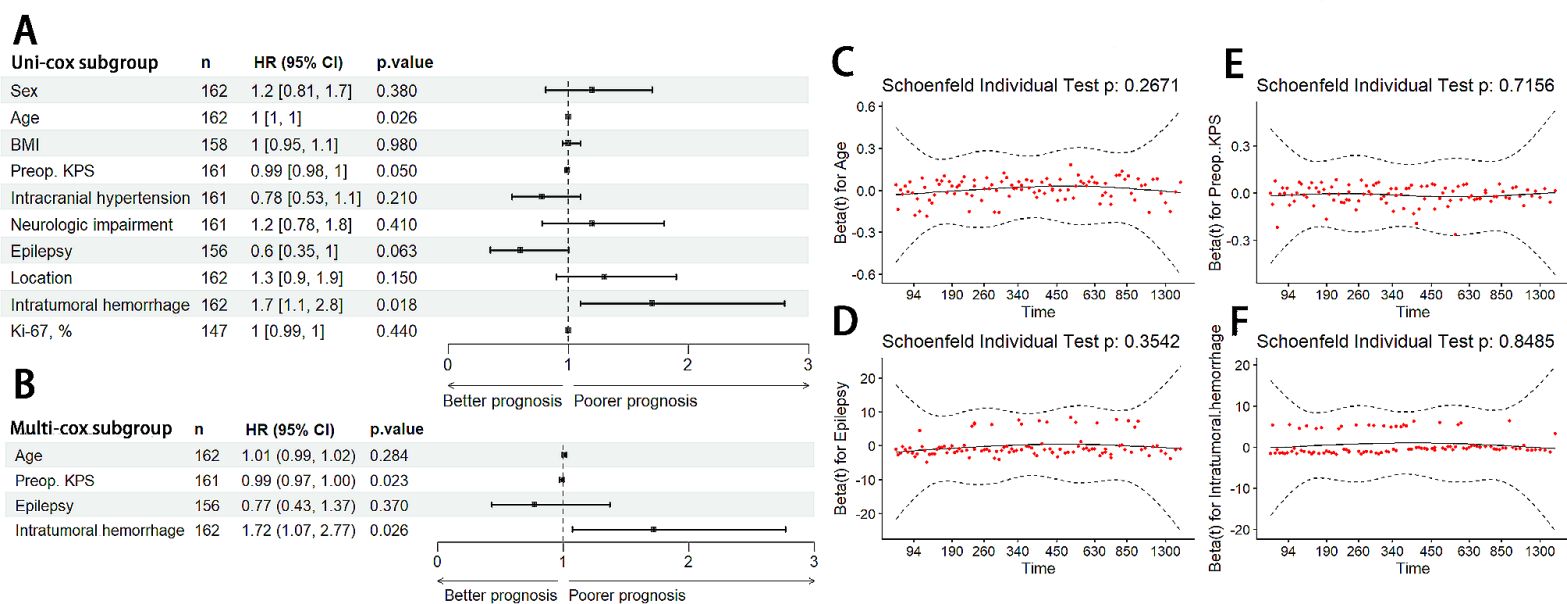
Uni-cox and multi-cox regression of prognostic risk factors in glioblastoma. **A**: Uni-cox regression analysis of glioblastoma showing that hemorrhage is a prognostic risk factor (HR = 1.7, *p* = 0.018). **B**: Multi-cox regression analysis indicating that hemorrhage is an independent prognostic risk factor in glioblastoma (HR = 1.72, *p* = 0.026) **C-F**: Analysis of proportional hazard assumption utilizing Schoenfeld residuals

Cox regression was used to identify variables with significant impact on the survival of glioma patients. In the subgroup analysis of astrocytoma (IDH mutant, WHO grade 2 and 3), oligodendroglioma (IDH mutant and 1p/19q co-deleted, WHO grade 2 and 3) and astrocytoma (IDH mutant, WHO grade 4), the cox regression result showed no significant impact of hemorrhage on prognosis (Figure S2, Figure A-C). In the subgroup analysis of patients with GBM, the univariate Cox regression analysis indicated that intratumoral hemorrhage was a significant adverse risk factor associated with a worse prognosis (HR = 1.7, *p* = 0.018, Fig. 3A). In multivariate analysis, both preoperative KPS and intratumoral hemorrhage were significant independent risk factors, with intratumoral hemorrhage as a negative poor prognostic factor (HR = 1.72, *p* = 0.026, Fig. 3B and C).

### Molecular signatures of patients with intratumoral hemorrhage

**Fig. 4:**
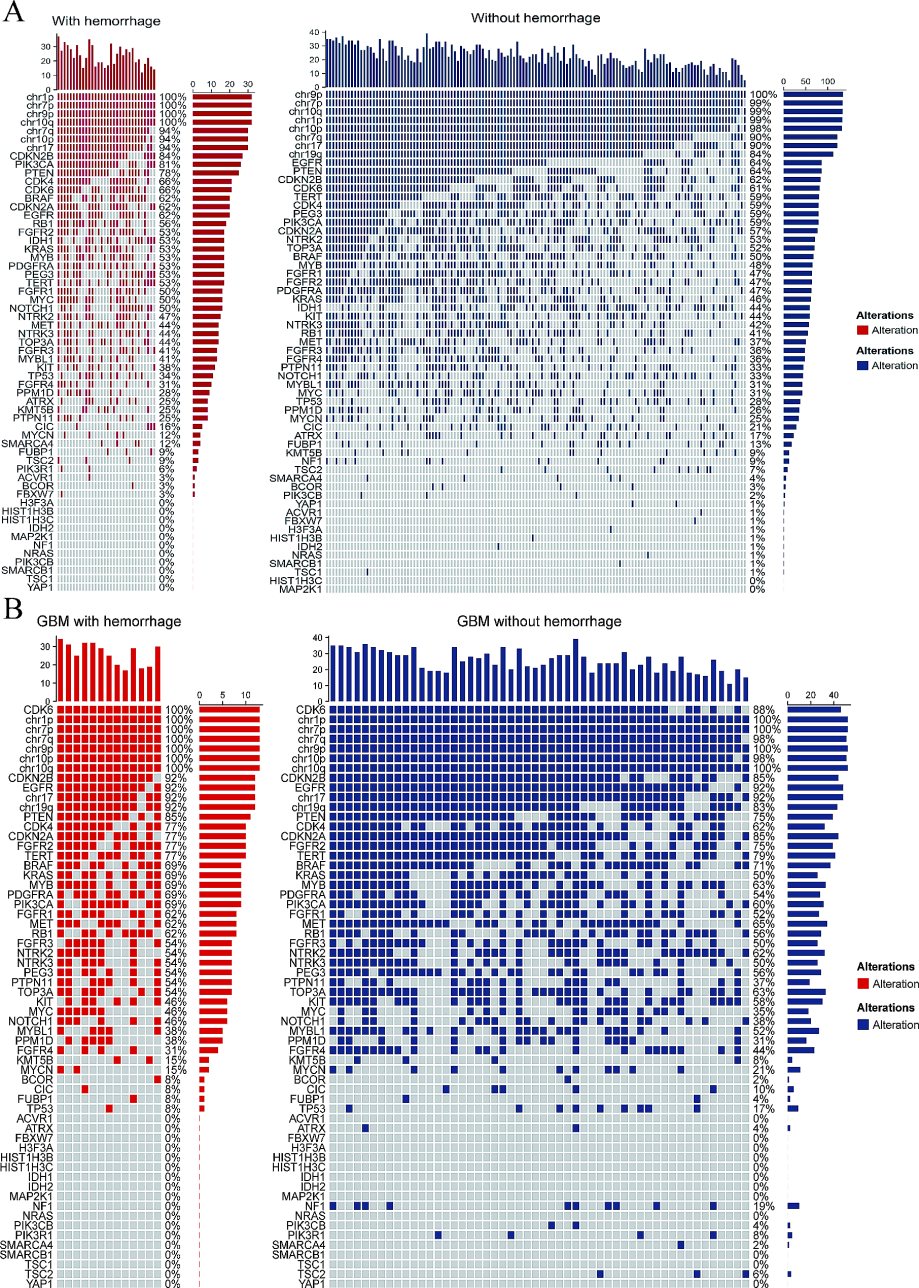
Molecular alterations of glioblastomas with and without hemorrhage **A**: This figure shows the genetic alterations of glioblastomas with and without hemorrhage. **B**: This figure shows the genetic alterations of glioblastomas with and without hemorrhage. Each column represents an individual patient, and the classification of tumors is displayed at the bottom. Each row indicates a genetic parameter listed from top to bottom based on the frequency of genetic alterations. The frequency of molecular alteration in each patient is shown in the right histogram

The molecular alterations in the with and without hemorrhage groups are summarized in Table 2; Fig. 4. A total of 167 patients had comprehensive molecular profiles: 32 in the hemorrhage group and 135 in the non-hemorrhage group. Alterations of CDKN2B, KMT5B, and PIK3CA were significantly different between the two groups, with higher alteration rates in the hemorrhage group (CDKN2B, 84.4% vs. 62.2%, *p* = 0.029; KMT5B, 25.0% vs. 8.9%, *p* = 0.029; and PIK3CA, 81.3% vs. 58.5%, *p* = 0.029), indicating that these molecular alterations may relate to the occurrence and potential mechanism of intratumoral hemorrhage. Other molecular alterations showed no statistical differences between the two groups (Table 2).

**Table 2:** Genetic alterations of glioma patients with and without intratumoral hemorrhage

gene alteration	All patients (*n* = 167)^1^	Patients with intra-tumoral hemorrhage (*n* = 32)	Patients without intra-tumoral hemorrhage (*n* = 135)	*P* value
ACVR1 alteration	2, 1.2%	1, 3.1%	1, 0.7%	0.3474
ATRX alteration	31, 18.6%	8, 25.0%	23, 17.0%	0.4302
BCOR alteration	5, 3.0%	1, 3.1%	4, 3.0%	1
BRAF alteration	88, 52.7%	20, 62.5%	68, 50.4%	0.2989
CDK4 alteration	100, 59.9%	21, 65.6%	79, 58.5%	0.5914
CDK6 alteration	103, 61.7%	21, 65.6%	82, 60.7%	0.7575
CDKN2A alteration	97, 58.1%	20, 62.5%	77, 57.0%	0.716
CDKN2B alteration	111, 66.5%	27, 84.4%	84, 62.2%	0.02938*
CIC alteration	34, 20.4%	5, 15.6%	29, 21.5%	0.6202
EGFR alteration	106, 63.5%	20, 62.5%	86, 63.7%	1
FBXW7 alteration	2, 1.2%	1, 3.1%	1, 0.7%	0.3474
FGFR1 alteration	80, 47.9%	16, 50.0%	64, 47.4%	0.9464
FGFR2 alteration	81, 48.5%	17, 53.1%	64, 47.4%	0.7001
FGFR3 alteration	61, 36.5%	13, 40.6%	48, 35.6%	0.7404
FGFR4 alteration	58, 34.7%	10, 31.3%	48, 35.6%	0.7999
FUBP1 alteration	21, 12.6%	3, 9.4%	18, 13.3%	0.7682
H3F3A alteration	1, 0.6%	0, 0.0%	1, 0.7%	1
HIST1H3B alteration	1, 0.6%	0, 0.0%	1, 0.7%	1
IDH1 alteration	76, 45.5%	17, 53.1%	59, 43.7%	0.4444
IDH2 alteration	1, 0.6%	0, 0.0%	1, 0.7%	1
KIT alteration	71, 42.5%	12, 37.5%	59, 43.7%	0.6604
KMT5B alteration	20, 12.0%	8, 25.0%	12, 8.9%	0.02851*
KRAS alteration	79, 47.3%	17, 53.1%	62, 45.9%	0.5916
MET alteration	64, 38.3%	14, 43.8%	50, 37.0%	0.617
MYB alteration	82, 49.1%	17, 53.1%	65, 48.1%	0.7568
MYBL1 alteration	55, 32.9%	13, 40.6%	42, 31.1%	0.412
MYC alteration	58, 34.7%	16, 50.0%	42, 31.1%	0.07009
MYCN alteration	38, 22.8%	4, 12.5%	34, 25.2%	0.1921
NF1 alteration	12, 7.2%	0, 0.0%	12, 8.9%	0.1255
NOTCH1 alteration	60, 35.9%	16, 50.0%	44, 32.6%	0.1009
NRAS alteration	1, 0.6%	0, 0.0%	1, 0.7%	1
NTRK2 alteration	86, 51.5%	15, 46.9%	71, 52.6%	0.7001
NTRK3 alteration	71, 42.5%	14, 43.8%	57, 42.2%	1
PDGFRA alteration	80, 47.9%	17, 53.1%	63, 46.7%	0.645
PEG3 alteration	96, 57.5%	17, 53.1%	79, 58.5%	0.7218
PIK3CA alteration	105, 62.9%	26, 81.3%	79, 58.5%	0.02856*
PIK3CB alteration	3, 1.8%	0, 0.0%	3, 2.2%	1
PIK3R1 alteration	11, 6.6%	2, 6.3%	9, 6.7%	1
PPM1D alteration	44, 26.3%	9, 28.1%	35, 25.9%	0.9755
PTEN alteration	111, 66.5%	25, 78.1%	86, 63.7%	0.1785
PTPN11 alteration	53, 31.7%	8, 25.0%	45, 33.3%	0.4843
RB1 alteration	73, 43.7%	18, 56.3%	55, 40.7%	0.1639
SMARCA4 alteration	9, 5.4%	4, 12.5%	5, 3.7%	0.06945
SMARCB1 alteration	1, 0.6%	0, 0.0%	1, 0.7%	1
TERT alteration	97, 58.1%	17, 53.1%	80, 59.3%	0.665
TOP3A alteration	84, 50.3%	14, 43.8%	70, 51.9%	0.5303
TP53 alteration	49, 29.3%	11, 34.4%	38, 28.1%	0.6315
TSC1 alteration	1, 0.6%	0, 0.0%	1, 0.7%	1
TSC2 alteration	12, 7.2%	3, 9.4%	9, 6.7%	0.702
YAP1 alteration	2, 1.2%	0, 0.0%	2, 1.5%	1
chr1p alteration	165, 98.8%	32, 100.0%	133, 98.5%	1
chr7p alteration	166, 99.4%	32, 100.0%	134, 99.3%	1
chr7q alteration	152, 91.0%	30, 93.8%	122, 90.4%	0.7384
chr10p alteration	162, 97.0%	30, 93.8%	132, 97.8%	0.2442
chr10q alteration	166, 99.4%	32, 100.0%	134, 99.3%	1
chr17 alteration	152, 91.0%	30, 93.8%	122, 90.4%	0.7384
chr19q alteration	144, 86.2%	31, 96.9%	113, 83.7%	0.08224

^1^ The results of molecular alteration are available only in 167 out of 457 patients due to retrospective design and lack of tissue specimen

### Implications of molecular alterations for survival in patients with intratumoral hemorrhage

**Fig. 5:**
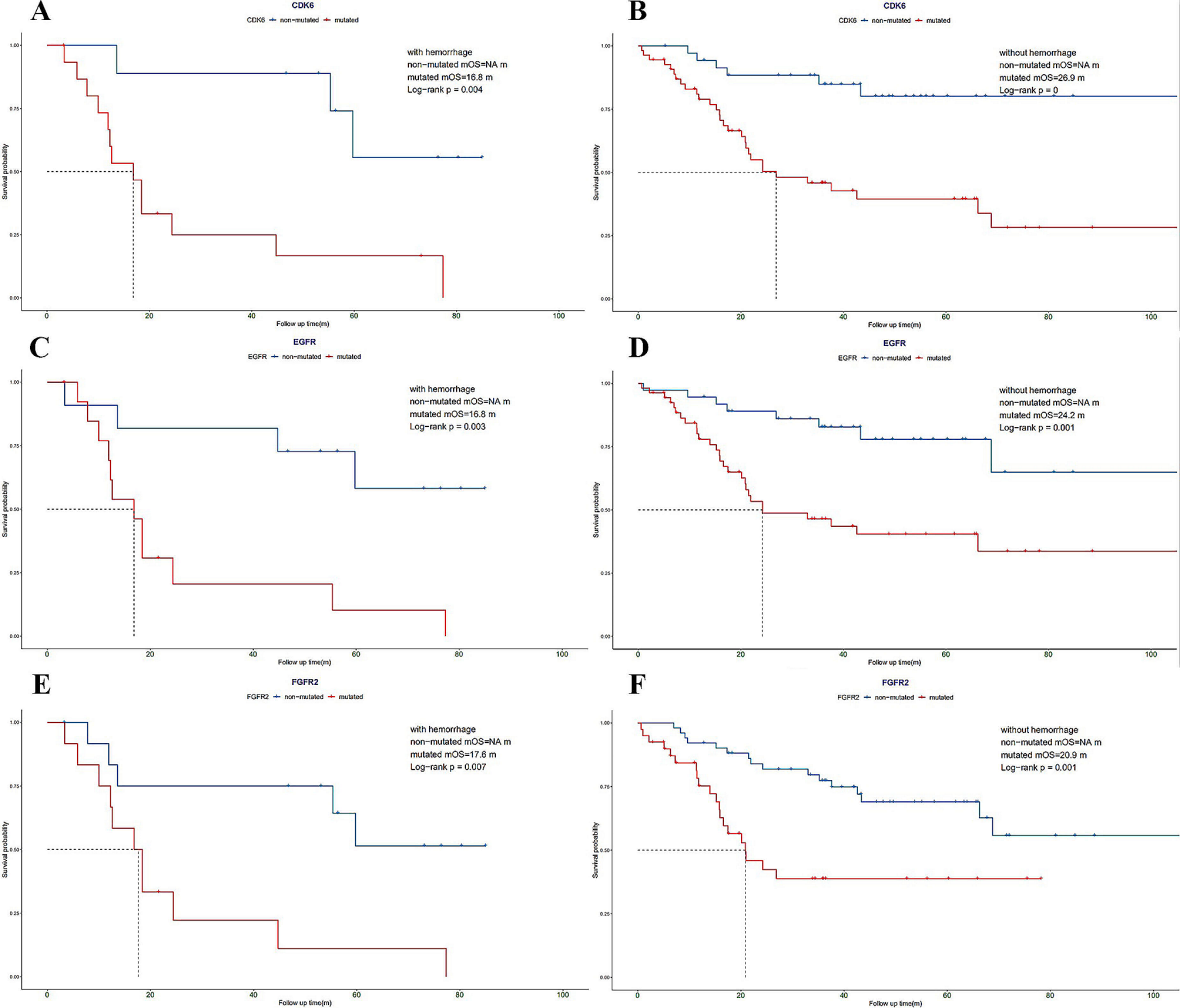
Molecular traits with significant prognostic significance in both non-hemorrhage and hemorrhage patients. This figure shows the Kaplan-Meier curves of CDK6, EGFR, and FGFR2 in the hemorrhage(**A**, **C** and **E**) and nonhemorrhage (**B**, **D** and **F**) groups

Due to the statistical significance of molecular alterations on the prognosis of patients with glioma, Kaplan-Meier survival analysis was also conducted to clarify the association of molecular markers and survival time for patients with and without hemorrhage in an attempt to find potential clues for prognostic prediction and more informed clinical decision making. The molecular alteration of CDK6, EGFR, and FGFR2 was each associated with shorter overall survival time of patients in both the hemorrhage and non-hemorrhage groups (Fig. 5), and the alteration of CDKN2A/B, FGFR3, MET, MYB, MYBL1, IDH1, and TERT showed prognostic significance in the non-hemorrhage group but displayed no difference in the hemorrhage group (Figure S3). Additionally, no correlation was observed between other molecular alterations and the survival time of patients in either group (Figure S4).

### Construction of Hemorrhage Prediction Model using Clinical and Imaging features

**Fig. 6:**
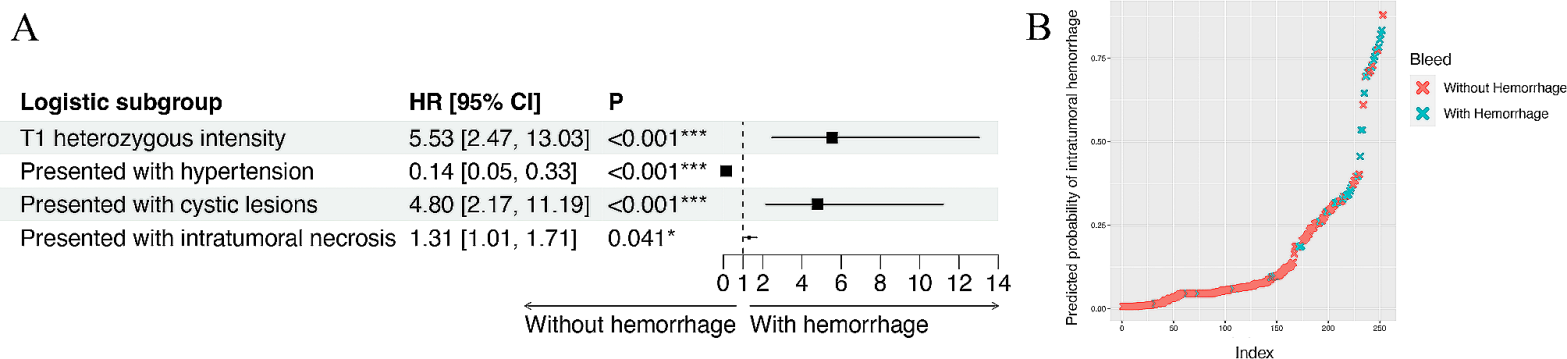
Logistic regression of predictive factors of intratumoral hemorrhage. **A**: Results of logistic regression showing that T1 heterozygous signaling, presentation of hypertension, cystic lesions and intratumoral necrosis are predictive of tumor hemorrhage. **B**: The validation of a predictive model of intratumoral hemorrhage

Because intratumoral hemorrhage is a significant negative prognostic factor in glioma patients, Cox regression analysis was used to screen features and construct a model to predict the presence of intratumoral hemorrhage among glioma patients (Fig. 6A). Patients with heterozygous T1 signalling, hypertension, cystic lesions, and necrosis tend to have an intratumoral hemorrhage. The model was then applied to the current cohort and could separate patients with hemorrrhage from those without hemorrhage (Fig. 6B).

## Discussion

This study employs preoperative MRI to reveal the incidence of intratumoral hemorrhage in glioma and its negative impact on survival. Surprisingly, the subgroup analysis showed that preoperative intratumoral hemorrhage overshadowed other factors typically associated with a poorer prognosis, indicating that preoperative intratumoral hemorrhage might be a more critical indicator for clinicians. Furthermore, we identified several molecular alterations potentially linked to the worse prognosis of both groups, including alterations in CDK6, EGFR, and FGFR2. Finally, various clinical presentations and molecular alterations were associated with an increased occurrence of intratumoral hemorrhage, such as higher Ki-67 levels and changes in CDKN2B, KMT5B, and PIK3CA, which might be potential targets for future research in preoperative intratumoral hemorrhage.

14.7% of glioma patients in the study showed intratumoral hemorrhage on preoperative imaging. This is similar to the fraction reported in the literature, reaffirming that intratumoral hemorrhage is not a rare complication among glioma patients [4, 8, 11]. However, previous studies have often combined all types of brain tumors into a single group and have yet to include genetic and molecular signatures in subgroup analysis. Our study stratifies patients based on the updated 2021 WHO 5 classification, thus providing a more clinically relevant report on the effects of hemorrhage. Comparable to prior data, the higher tumor grade and astrocytomas/glioblastomas are associated with the highest occurrence of intratumoral hemorrhage in primary brain tumors [11]. Nevertheless, previous reports on intratumoral hemorrhage have primarily focused on metastatic brain tumor [13, 14] and rarely on primary brain tumors. In contrast, our study includes only adult diffuse glioma patients, and our results can, therefore, more accurately reflect the clinical outcomes of this specific patient population. In summary, the current study provides the most updated data on the incidence of intratumoral hemorrhages among adult glioma patients.

The survival analysis in this study suggests that even with similar preoperative performance between the two groups, the hemorrhage group had significantly higher Ki-67, more frequently exhibited neurologic impairment and had a worse prognosis (Table 1). Intratumoral hemorrhage was also an independent adverse prognostic factor in both univariate and multivariate analyses (Fig. 3). This finding is essential since although the outcomes of glioma patients might depend on various factors, the imaging-based intratumoral hemorrhage remains the independent adverse prognostic factor, highlighting the inclusion of such a feature into clinical management considerations. Moreover, this negative impact of hemorrhage can be used clinically to aid outcome prediction. For instance, Kong et al. applied susceptibility-weighted imaging based on microhemorrhage features in tumors to differentiate lower-grade gliomas from higher-grade ones [15]. More importantly, intratumoral hemorrhage is a feature that can be easily detected by noninvasive MRI imaging even before the surgery, thus offering an opportunity to tailor management. The results of our study support the predictive value of intratumoral hemorrhage and the use of simple, noninvasive imaging to stratify glioma patients preoperatively.

Interestingly, in subgroup analysis, the factors usually associated with worse prognosis, such as lower KPS, older age, and glioblastoma, became less predictive of outcome in intratumoral hemorrhage. This suggests that hemorrhage can be an independent negative predictor of survival, overriding the effects of age, KPS, and WHO classification. Consequently, this highlights the importance of clinical management modification in this subgroup of glioma patients. Some researchers have recommended anti-angiogenic treatment in malignant glioma, but its effectiveness, especially for patients already exhibiting intratumoral hemorrhage, remains to be determined, and future studies are needed [16].

Moreover, our study showed that Ki-67, an indicator of cell proliferation, differed significantly between hemorrhage and non-hemorrhage groups (Table 1); however, in the Cox analysis (Fig. 3), it was shown as a non-independent risk factor (*p* = 0.440), which indicates that the cell proliferation might be related to intratumoral hemorrhage. This aligns with prior research, which shows glioblastomas with different microvascular patterns have significantly different Ki-67 indices and survival times [17]. High proliferative activity with high oxygen consumption is more likely to cause hypoxia in tumors, stimulating HIF-1α overexpression [18] and thus promoting angiogenesis. Previous studies have reported that the intensity of microvessels increased with higher astrocytoma grade [19], and gliomas of different grades have different neovascularity [20]. The angiogenesis and the newly generated vessels, which lack tight junctions and are fragile, increase the risk of vessel rupture and hemorrhage [21, 22]. These results suggest angiogenesis as a potential explanation for the correlation between Ki-67 and tumor-associated hemorrhage.

Surprisingly, hemorrhage did not correlate with glioma grade or classification (Table 1), considering that high-grade gliomas have more proliferation. However, the hemorrhage group did tend to have a higher percentage of high-grade gliomas (Table 1). Glioblastomas and astrocytomas also had a higher percentage of hemorrhage than oligodendrogliomas (Table 1). The higher rate of astrocytomas may be because 53.2% were graded 4. The insignificance may be due to the small sample size, which deserves further investigation.

Considering that intratumoral hemorrhage is associated with a worse prognosis, it is critical to identify biomarkers related to the presence of hemorrhage. The alterations of CDKN2B, KMT5B, MAP2K1, PIK3CA, and chr9p were associated with hemorrhage (Table 2), and this may be due to the fact that most of them affect glioma cell proliferation and invasion. KMT5B encodes a kind of histone lysine methyltransferases (KMTs), which employs H4K20me1 as a substrate, giving rise to H4K20me2 [23]. Previous studies have shown that KMT5B overexpression reduces the proliferation of glioblastoma cells [24], and enhanced activity of KMT5B can suppress VEFGR2 expression in endothelial cells [25]. In our study population, all KMT5B alterations were point mutations and likely caused loss of function, which may explain why patients with intra-tumoral hemorrhage tended to have more KMT5B alterations.

The survival analysis identified the following three molecular features that had a significant prognostic impact in both hemorrhage and nonhemorrhage groups: CDK6, EGFR, and FGFR2. First, CDK6 is a cell cycle regulation-related molecule, and its mutations often lead to enhanced cell cycle protein-dependent kinase six activity and tumor growth [26], consistent with our study’s significant prognostic differences. EGFR and FGFR2 play an essential role in tumor angiogenesis and, therefore, may increase the probability of hemorrhage, which may, in turn, lead to a poorer prognosis [27]. However, in this study, the prognostic deterioration due to these two molecular features was not related to the presence or absence of hemorrhage, and we, therefore, speculate that the prognostic impact of the angiogenic effects of EGFR and FGFR2 is more likely to arise through the promotion of tumor proliferation and migration than through hemorrhage.

Nevertheless, ten genetic alterations only affected prognosis among patients without hemorrhage: CDKN2A/B, FGFR3, MET, MYB, MYBL1, IDH1, TERT, PTEN, and RB1. CDKN2A/B encode p16/p15 proteins and their inactivation results in the acceleration of tumor progression [28, 29]. FGFR3 and MET play critical roles in tumor growth [30, 31]. MYB and MYBL1 regulate the expression of cell cycle protein-dependent kinases, which contribute to cancer development [32]. However, overexpression of wild-type MYB is insufficient to transform human epithelial cells fully; it promotes tumorigenesis only in combination with additional genetic alterations [33]. IDH1 mutations are prevalent and associated with better prognosis in gliomas [34]. TERT promoter mutations can lead to TERT transcription upregulation and cell proliferation [35]. RB1 and PTEN are both negative regulators of cells [36, 37].

The mechanisms described above indicate the effects of these genes in the without hemorrhage group; mutations can lead to significant differences in prognosis. However, in the hemorrhage group, we found that hemorrhage can mask the prognostic differences of these genes, regardless of their original prognostic impact. In particular, IDH1 and TERT have a significant prognostic impact on gliomas to the extent that they can be used as clear criteria for CNS tumor classification [38]. Still, as we previously mentioned, they do not predict survival in patients with hemorrhage. Therefore, Particular attention should be paid to these issues in a clinical setting, and a renewed prognosis-related molecular profile of patients with hemorrhage should be developed.

Lastly, we constructed a model using imaging and clinical features to predict intratumoral haemorrhage occurrence in glioma patients. This model included discernible clinical presentations, such as intracranial hypertension and intratumoral necrosis, to aid in early hemorrhage diagnosis (Fig. 6). Many studies have examined the negative impact of intratumoral necrosis on the prognosis of glioma patients, such as GBM and oligodendrocytoma [39, 40]. However, the association between necrosis and intratumoral hemorrhage has not been explored. In our hemorrhage prediction model, necrosis is a positive risk factor for intratumoral hemorrhage. Whether there is a common molecular mechanism underlying these imaging features is worthy of further investigation.

However, our study still has some limitations. First, since the study was retrospective and intratumoral hemorrhage was defined based on imaging presentations, differences between radiological features and anatomical presentation may remain, leading to ambiguity during group division. Future studies should explore the differences in patient characteristics between histological versus imaging-defined intratumoral hemorrhage. Second, there may have been potential selection bias due to the small sample of this retrospective study, especially the unbalanced number in hemorrhage and non-hemorrhage groups and the subgroup analysis of genetic alterations. However, the unbalanced number in the two groups may also highlight the intrinsic occurrence of intratumoral hemorrhage among glioma patients. Therefore, future studies with larger patient populations are needed to validate the results of our study. Third, we pre-designed a molecular panel that was essential in characterizing the survival of glioma patients, however, more molecular biomarkers might be related to intratumoral hemorrhage, and in the future, whole-exome sequencing might be a potential solution to address this problem.

## Conclusions

This retrospective cohort study showed that glioma patients with preoperative intratumoral hemorrhage had a worse prognosis, regardless of age, KPS or glioma grades. CDKN2B, KMT5B, and PIK3CA alterations were common in the hemorrhage group, suggesting a possible mechanism for the prognostic value of intratumoral hemorrhage. Additionally, several molecular alterations concerning prognosis were identified in both groups and warrant further investigation.

### Electronic supplementary material

Below is the link to the electronic supplementary material.

Supplementary Material 1

Supplementary Material 2

Supplementary Material 3

Supplementary Material 4

## Data Availability

No datasets were generated or analysed during the current study.

## Abbreviations

BMI: Body mass index
CDKN2A/B: Cyclin-dependent kinase inhibitor 2 A or 2B
CDKN2A: Cyclin-dependent kinase inhibitor 2 A
CDKN2B: Cyclin-dependent kinase inhibitor 2B
CDKN2B-AS1: CDKN2B antisense RNA 1
CDK6: cyclin-dependent kinase 6
Chr9p: Chromosome 9 short arm
EGFR: Epidermal growth factor receptor
FGFR2: Fibroblast growth factor receptor 2
FGFR3: Fibroblast growth factor receptor 3
GBM: Glioblastoma
H4K20me1: Monomethylation of histone H4 Lys 20 methylation
HIF-1α: Hypoxia-inducible factor 1-alpha
IDH: Isocitrate dehydrogenase
KMT: Histone lysine methyltransferase
KMT5B: Lysine methyltransferase 5B
KPS: Karnofsky performance score
MAP2K1: Mitogen-activated protein kinase kinase 1
MET: MET proto-oncogene, receptor tyrosine kinase
mOS: Median overall survival
MRI: Magnetic resonance imaging
MYB: MYB proto-oncogene, transcription factor
MYBL1: MYB proto-oncogene like 1
OS: Overall survival
PI3K: Phosphoinositide 3-kinase
PIK3CA: Phosphatidylinositol-4,5-bisphosphate 3-kinase catalytic subunit alpha
PTEN: Phosphatase and tensin homolog
RB1: RB transcriptional corepressor 1
TERT: Telomerase reverse transcriptase
VEGFR2: Vascular endothelial growth factor receptor 2
WHO CNS 5 classifications: The 2021 WHO classification of tumors of the central nervous system, 5th edition
