# Supplementary material for: The molecular signature and prognosis of glioma with preoperative intratumoral hemorrhage: a retrospective cohort analysis
Source: BMC Neurol. 2024 Jun 14;24:202. doi: 10.1186/s12883-024-03703-2 (PMC11177380; doi:10.1186/s12883-024-03703-2)
Supplement: Supplementary file 1 — Supplementary Material 1 [file 12883_2024_3703_MOESM1_ESM.docx]

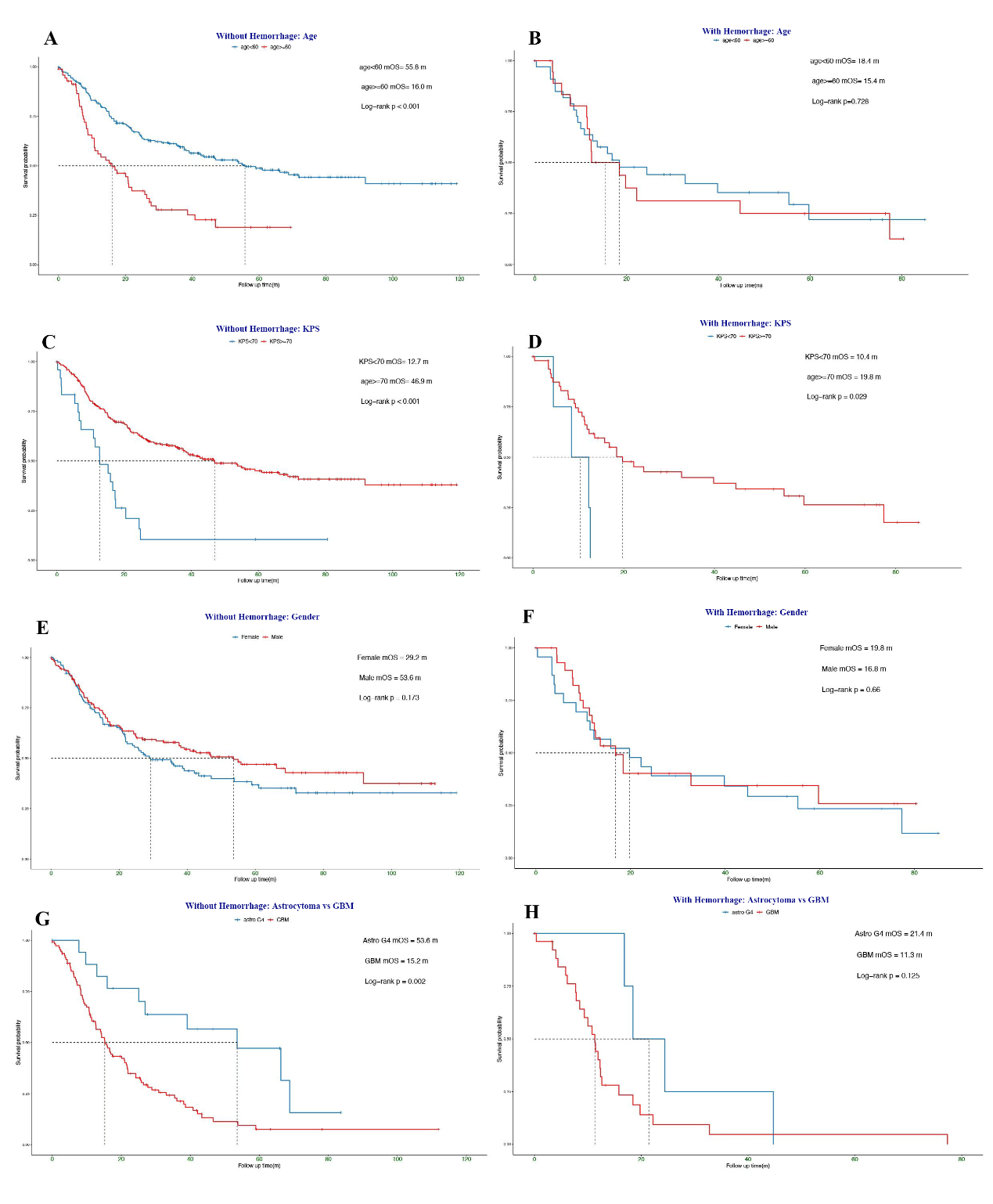


**FigS1 Comparisons of** **overall survival (OS) between the hemorrhage and no hemorrhage groups with different baseline characteristics**

We compared the differences in OS regarding age, preoperative KPS score, and gender in patients without intratumoral hemorrhage (**A**, **C**, and **E**) and with hemorrhage in postoperative imaging (**B**, **D**, and **F**). **A**: Patients younger than 60 years old had a significantly longer OS in the nonhemorrhage group (mOS: 55.8 vs. 16.0 months, *P* < 0.001). **B**: No significant difference in survival in the hemorrhage group (mOS: 18.4 vs. 15.4 months, *P* = 0.728). **C** and **D**: In both groups, patients with KPS score less than 70 had a significantly shorter OS (nonhemorrhage: mOS 12.7m for KPS<70, mOS 46.9m for KPS≥70, *p* < 0.001; hemorrhage: mOS 10.4 vs. 19.8m, *p* = 0.029). **E** and **F**: No significant differences in OS were seen between females (F) and males (M) in either group (nonhemorrhage: mOS 29.2m for F vs. 53.6 for M, *p* = 0.173; hemorrhage: mOS 19.8 for F vs. 16.8 for M, *p* = 0.66). **G** and **H**: differences in OS when comparing glioblastoma, IDH wildtype, grade 4 (GBM) with astrocytoma, IDH mutant, grade 4 (Astro G4). A significant difference in OS was only observed in the nonhemorrhage group (mOS 15.2m for GBM vs. 53.6m for Astro G4, *p* = 0.002), not in the hemorrhage group (mOS 11.3m for GBM vs. 21.4m for Astro G4, *p* = 0.125)
