# Supplementary material for: The molecular signature and prognosis of glioma with preoperative intratumoral hemorrhage: a retrospective cohort analysis
Source: BMC Neurol. 2024 Jun 14;24:202. doi: 10.1186/s12883-024-03703-2 (PMC11177380; doi:10.1186/s12883-024-03703-2)
Supplement: Supplementary file 2 — Supplementary Material 2 [file 12883_2024_3703_MOESM2_ESM.docx]

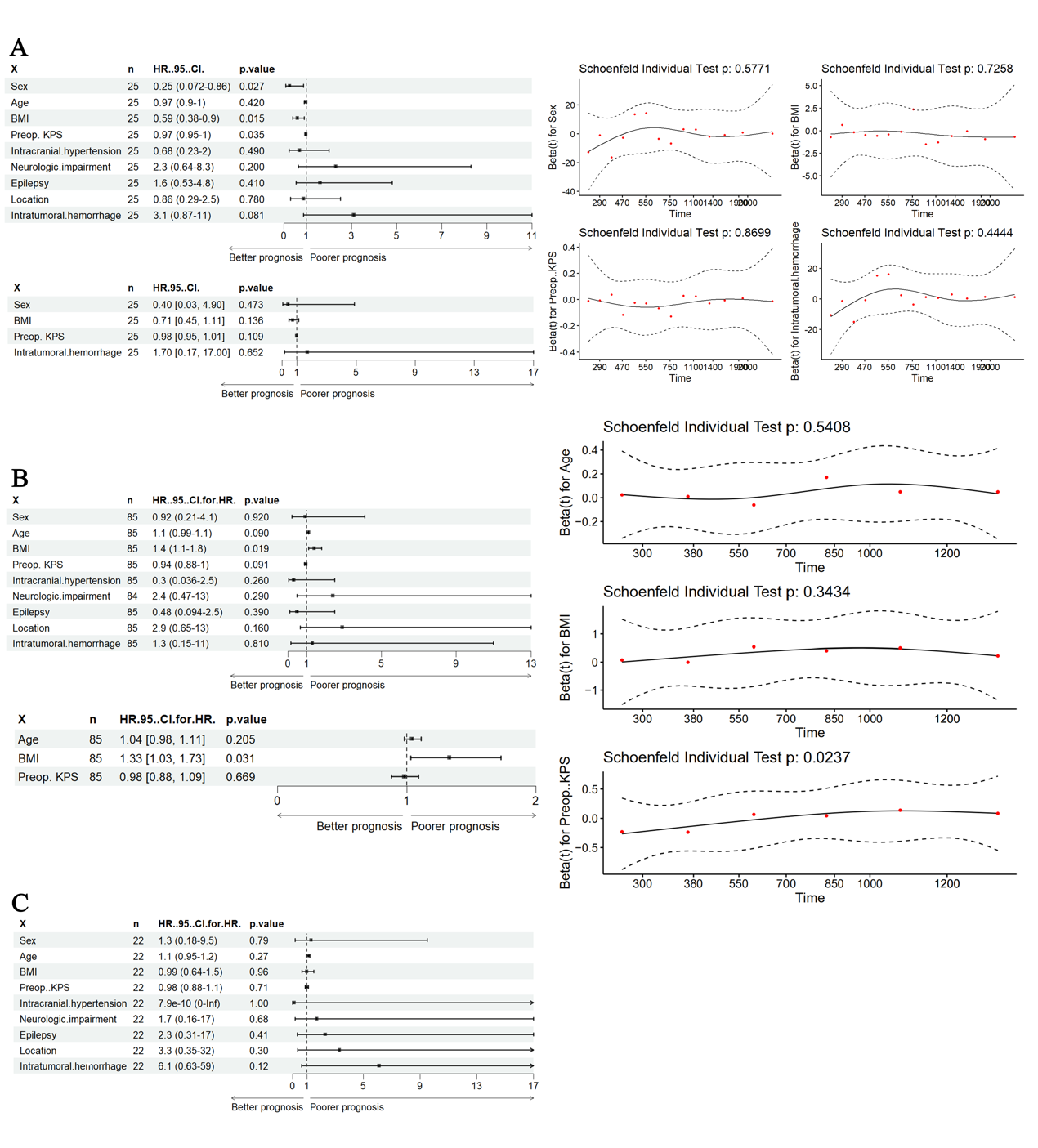


**FigS2. Uni-cox and Multi-cox regression of prognostic factors of glioma patients in different subgroups.**

This figure shows uni-cox and multi-cox regression result of prognostic factors in different subtypes of glioma patients, including astrocytoma, IDH mutant, WHO grade 2 and 3; oligodendroglioma, IDH mutant and 1p/19q co-deleted, WHO grade 2 and 3; and astrocytoma, IDH mutant, WHO grade 4 (A-C respectively).
