# Supplementary material for: The molecular signature and prognosis of glioma with preoperative intratumoral hemorrhage: a retrospective cohort analysis
Source: BMC Neurol. 2024 Jun 14;24:202. doi: 10.1186/s12883-024-03703-2 (PMC11177380; doi:10.1186/s12883-024-03703-2)
Supplement: Supplementary file 3 — Supplementary Material 3 [file 12883_2024_3703_MOESM3_ESM.docx]

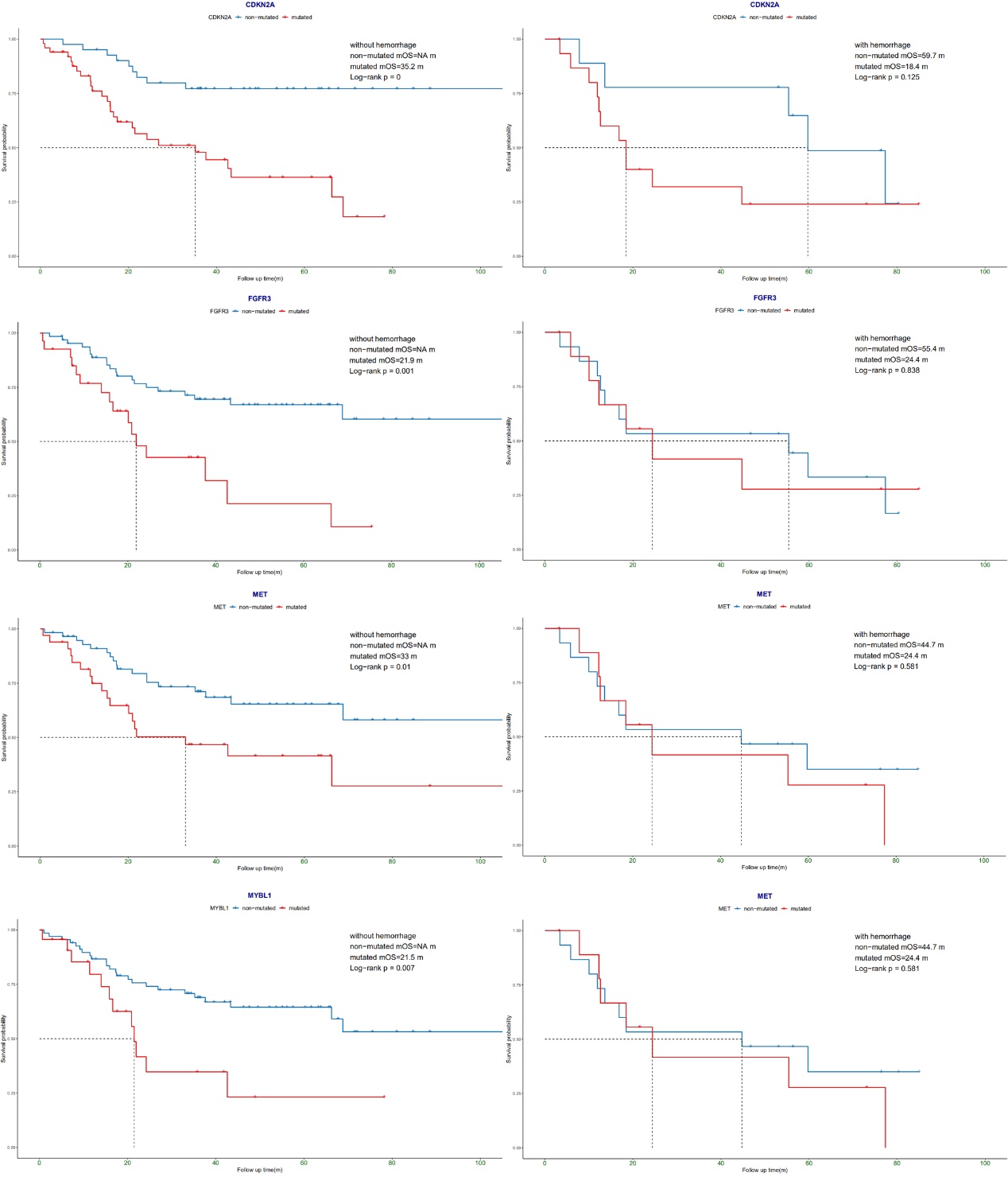


**FigS3 Molecular traits with significant prognostic significance in both the hemorrhage and nonhemorrhage groups**

This figure shows the Kaplan-Meier curves of CDK6, EGFR, and FGFR2 in the hemorrhage (A, C and E) and nonhemorrhage (B, D and F) groups.
