# Supplementary material for: The molecular signature and prognosis of glioma with preoperative intratumoral hemorrhage: a retrospective cohort analysis
Source: BMC Neurol. 2024 Jun 14;24:202. doi: 10.1186/s12883-024-03703-2 (PMC11177380; doi:10.1186/s12883-024-03703-2)
Supplement: Supplementary file 4 — Supplementary Material 4 [file 12883_2024_3703_MOESM4_ESM.pdf]

FigS4 Kaplan-Meier curves showing no significant effects of certain genetic alterations on the overall survival of patients with bleeding or without bleeding

Since the numbers of patients divided into four groups (e.g. BRAF wildtype, bleeding/ BRAF alteration, bleeding/ BRAF wildtype, non-bleeding/ BRAF alteration, non-bleeding) using different parameters varied, we only enrolled the parameters using which the number of patients in either group was above 3. Parameters used in this section were shown as follows.

(1) ATRX 1
(2) BRAF 3
(3) CDK4 5
(4) CIC 7
(5) FGFR1 9
(6) FGFR4 11
(7) KIT 13
(8) KMT5B 15
(9) KRAS 17
(10) MYC 19
(11) NOTCH1 21
(12) NTRK2 23
(13) NTRK3 25
(14) PDGFRA 27
(15) PEG3 29
(16) PIK3CA 31
(17) PPM1D 33
(18) PTPN11 35
(19) TERT 37
(20) TOP3A 39
(21) TP53 41

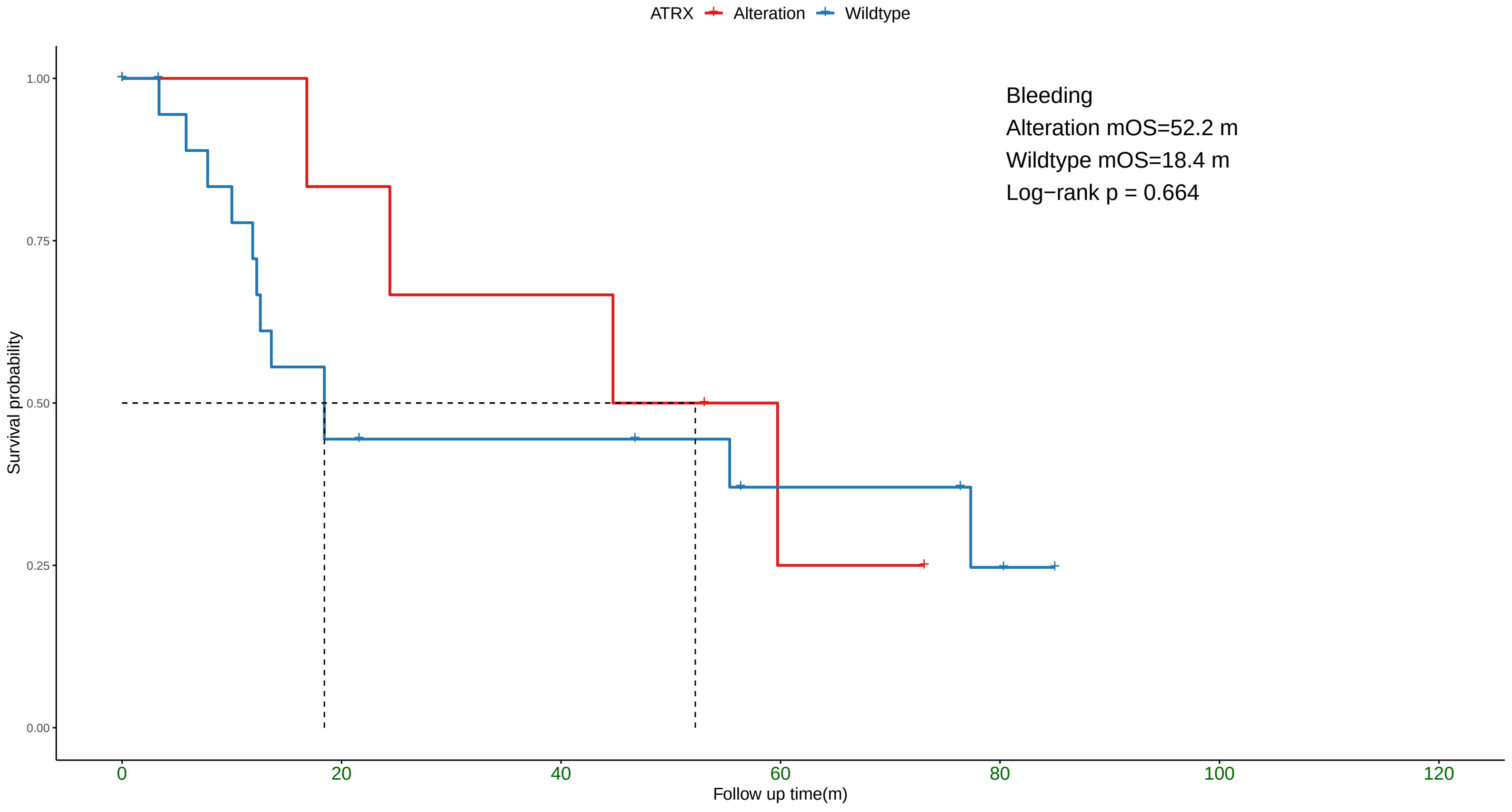
ATRX
Alteration
Wildtype
Bleeding
Alteration mOS=52.2 m
Wildtype mOS=18.4 m
Log−rank p = 0.664
Survival probability
1.00
0.75
0.50
0.25
0.00
0
20
40
60
80
100
120
Follow up time(m)

-2-

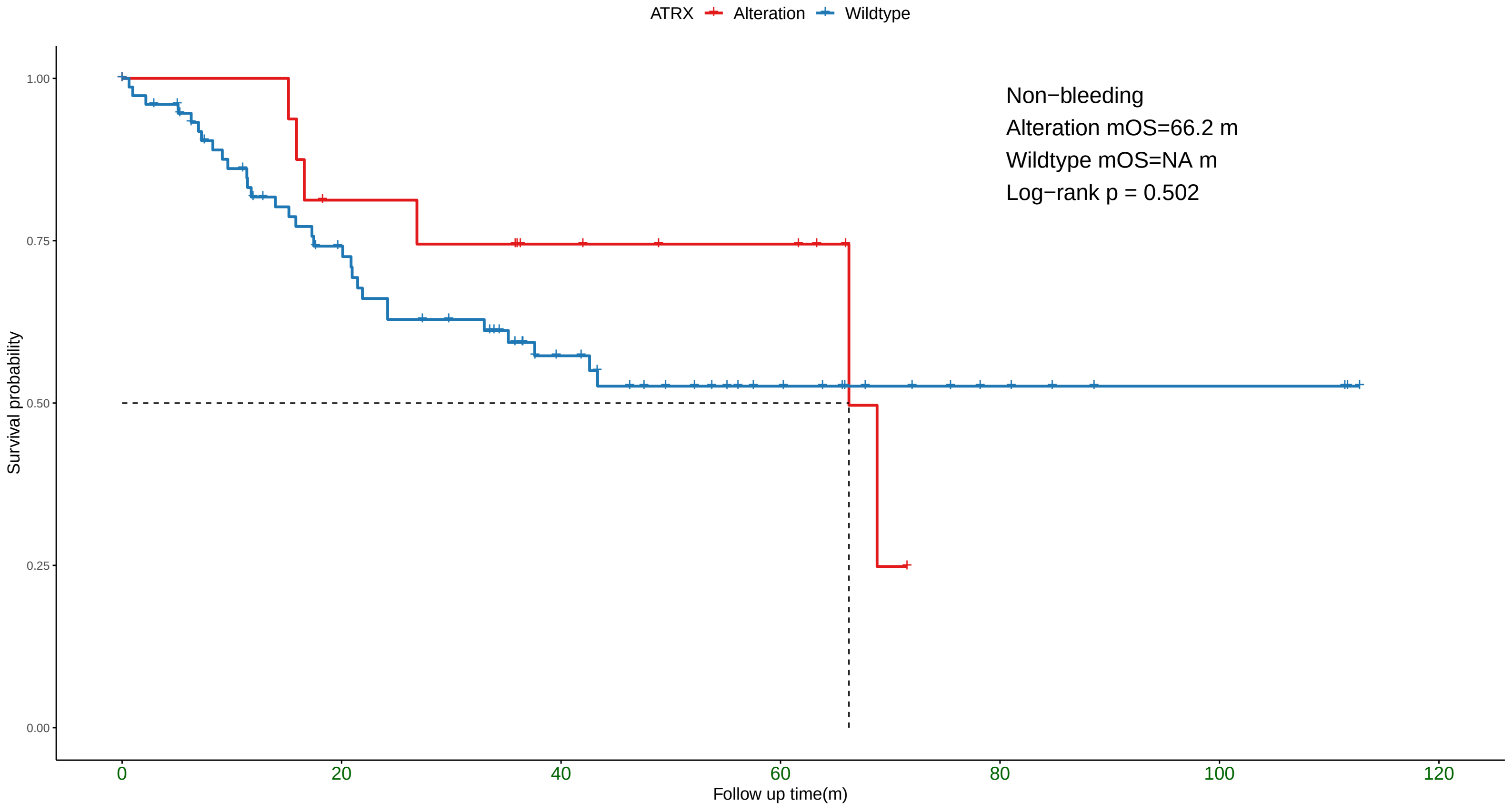

ATRX
Alteration
Wildtype
Non−bleeding
Alteration mOS=66.2 m
Wildtype mOS=NA m
Log−rank p = 0.502
Survival probability
1.00
0.75
0.50
0.25
0.00
0
20
40
60
80
100
120
Follow up time(m)

-3-

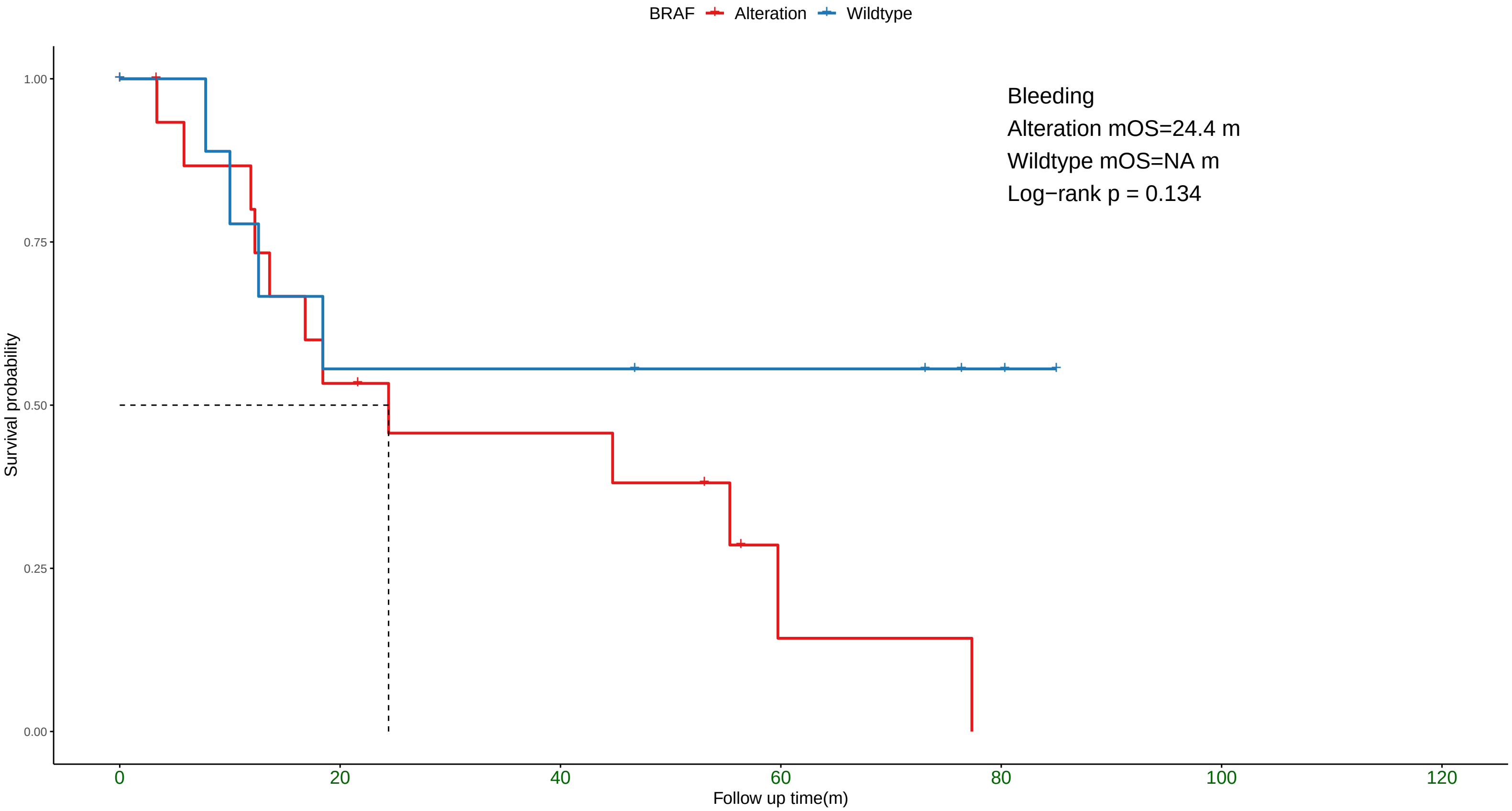

BRAF
Alteration
Wildtype
Bleeding
Alteration mOS=24.4 m
Wildtype mOS=NA m
Log−rank p = 0.134
Survival probability
1.00
0.75
0.50
0.25
0.00
0
20
40
60
80
100
120
Follow up time(m)

-4-

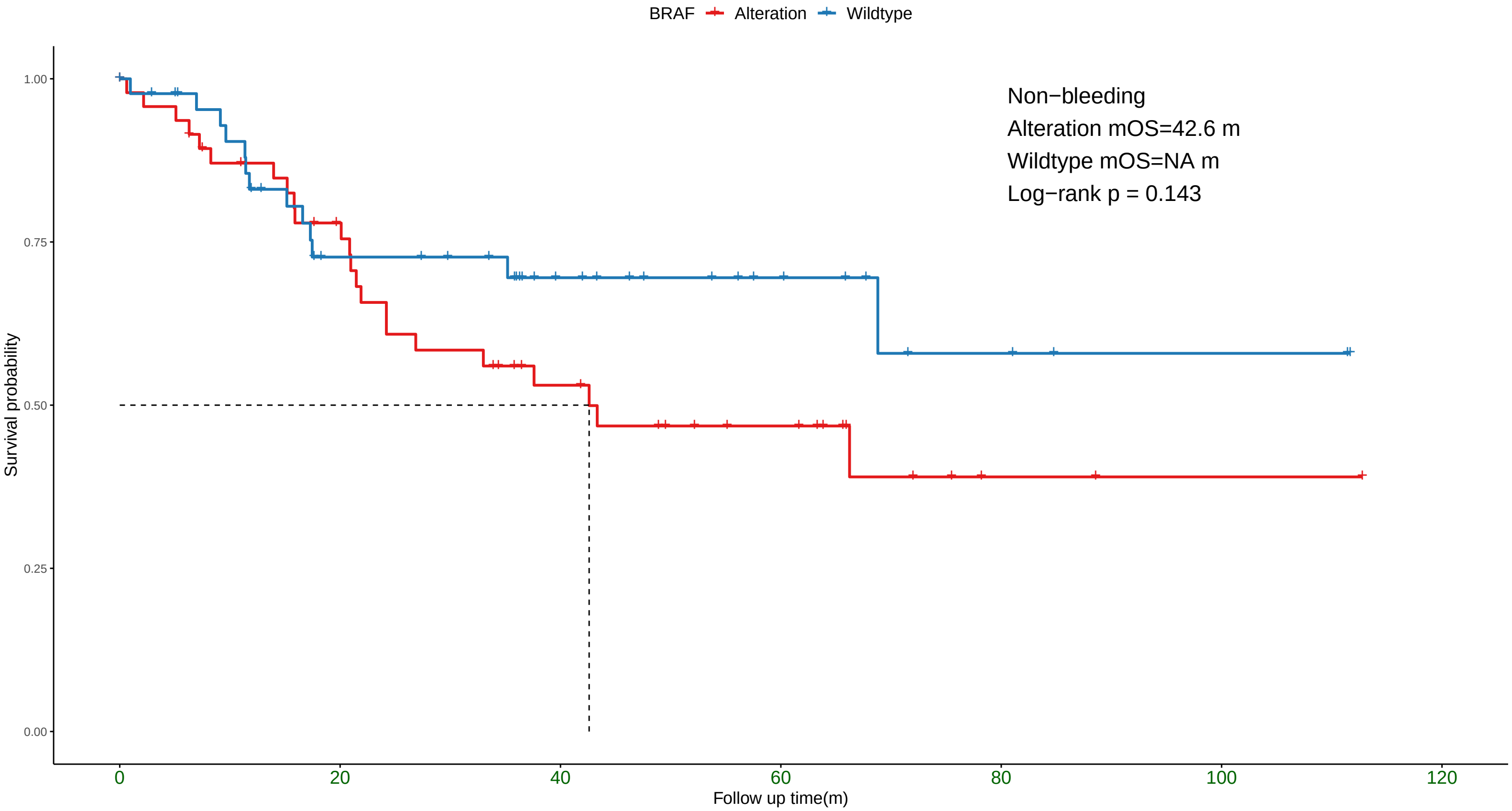
BRAF
Alteration
Wildtype
Non−bleeding
Alteration mOS=42.6 m
Wildtype mOS=NA m
Log−rank p = 0.143
Survival probability
1.00
0.75
0.50
0.25
0.00
0
20
40
60
80
100
120
Follow up time(m)

-5-

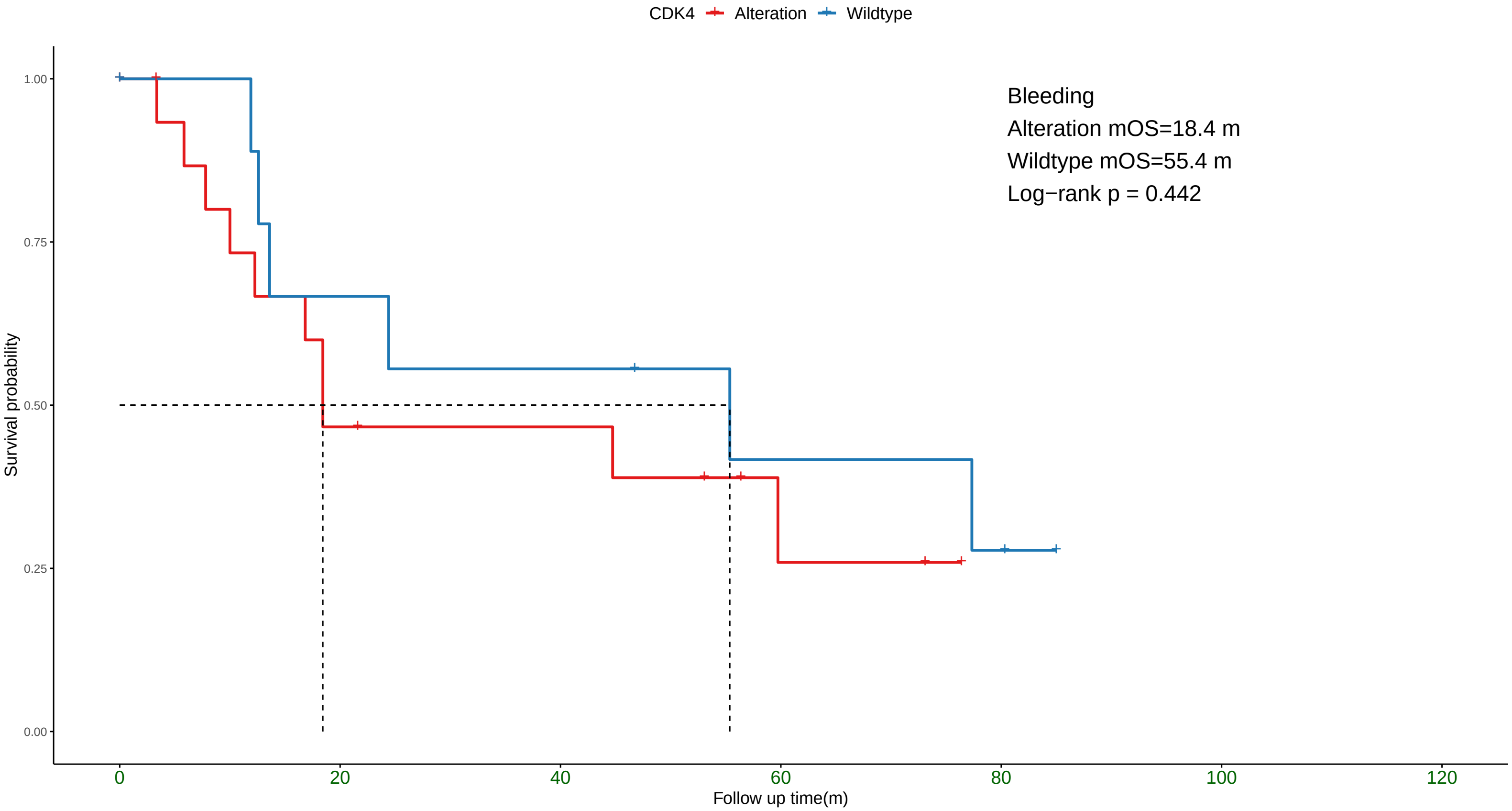

CDK4
Alteration
Wildtype
Bleeding
Alteration mOS=18.4 m
Wildtype mOS=55.4 m
Log−rank p = 0.442
Survival probability
1.00
0.75
0.50
0.25
0.00
0
20
40
60
80
100
120
Follow up time(m)

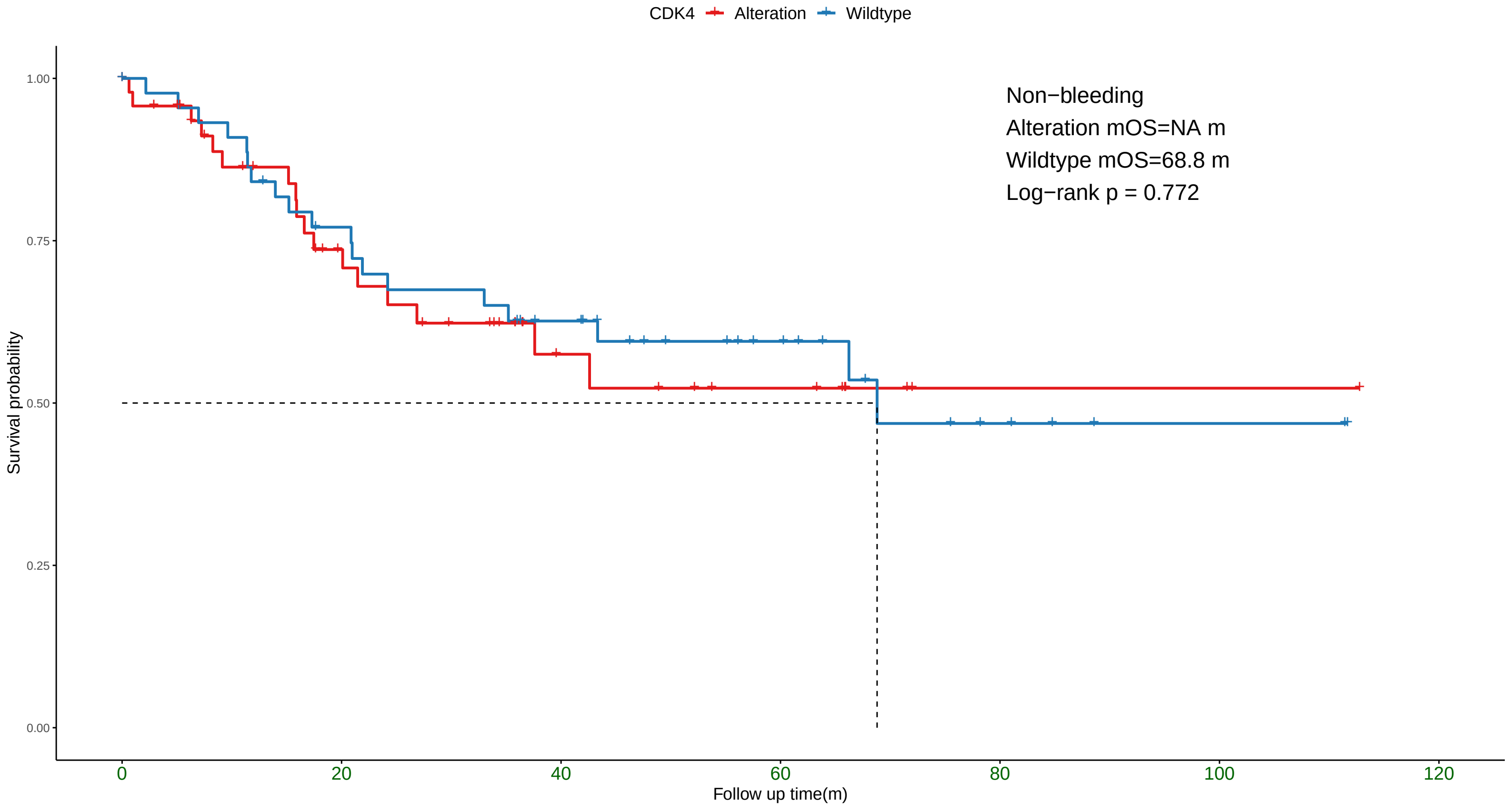
CDK4
Alteration
Wildtype
Non−bleeding
Alteration mOS=NA m
Wildtype mOS=68.8 m
Log−rank p = 0.772
Survival probability
1.00
0.75
0.50
0.25
0.00
0
20
40
60
80
100
120
Follow up time(m)

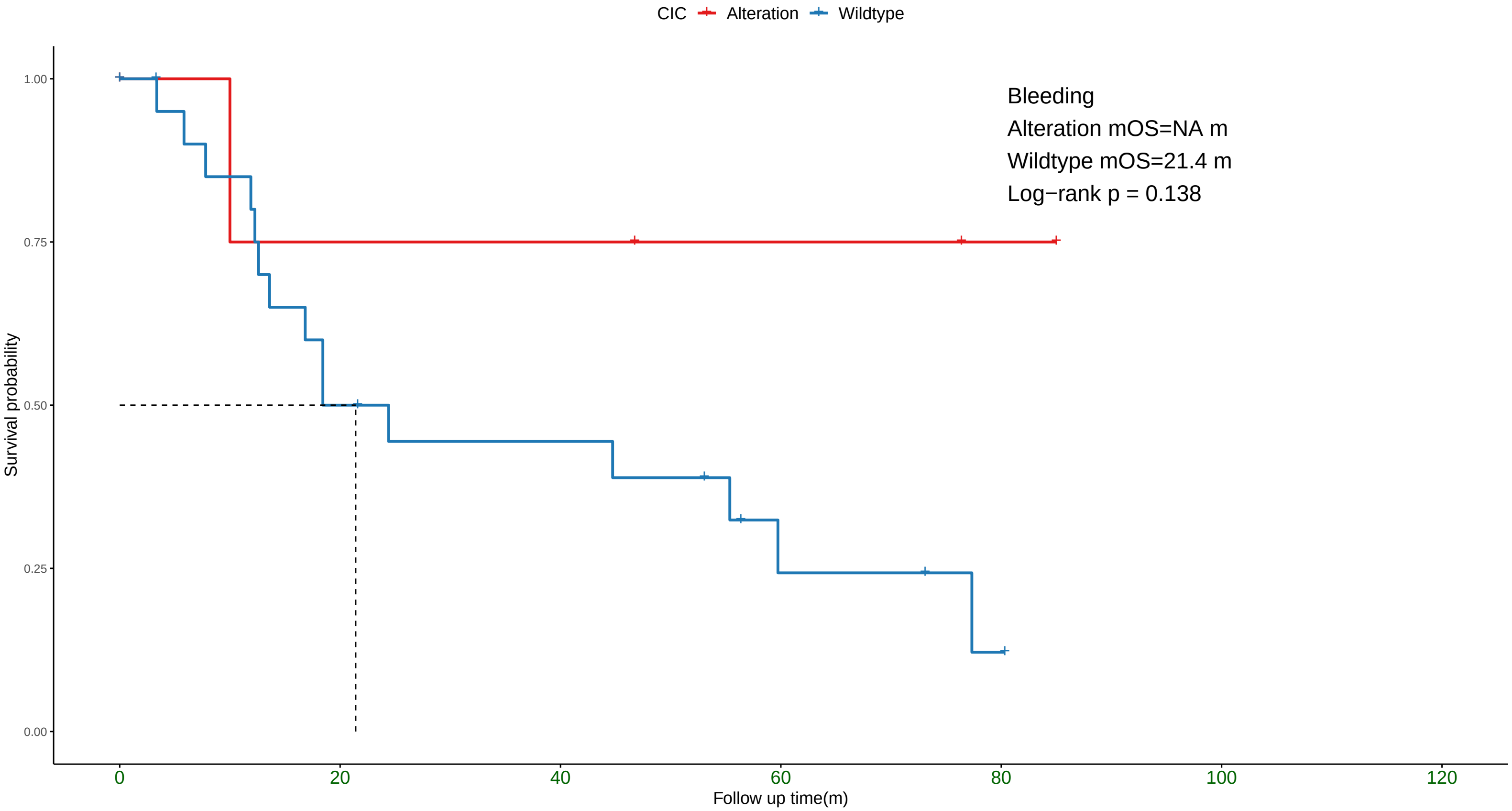
CIC
Alteration
Wildtype
Bleeding
Alteration mOS=NA m
Wildtype mOS=21.4 m
Log−rank p = 0.138
Survival probability
1.00
0.75
0.50
0.25
0.00
0
20
40
60
80
100
120
Follow up time(m)

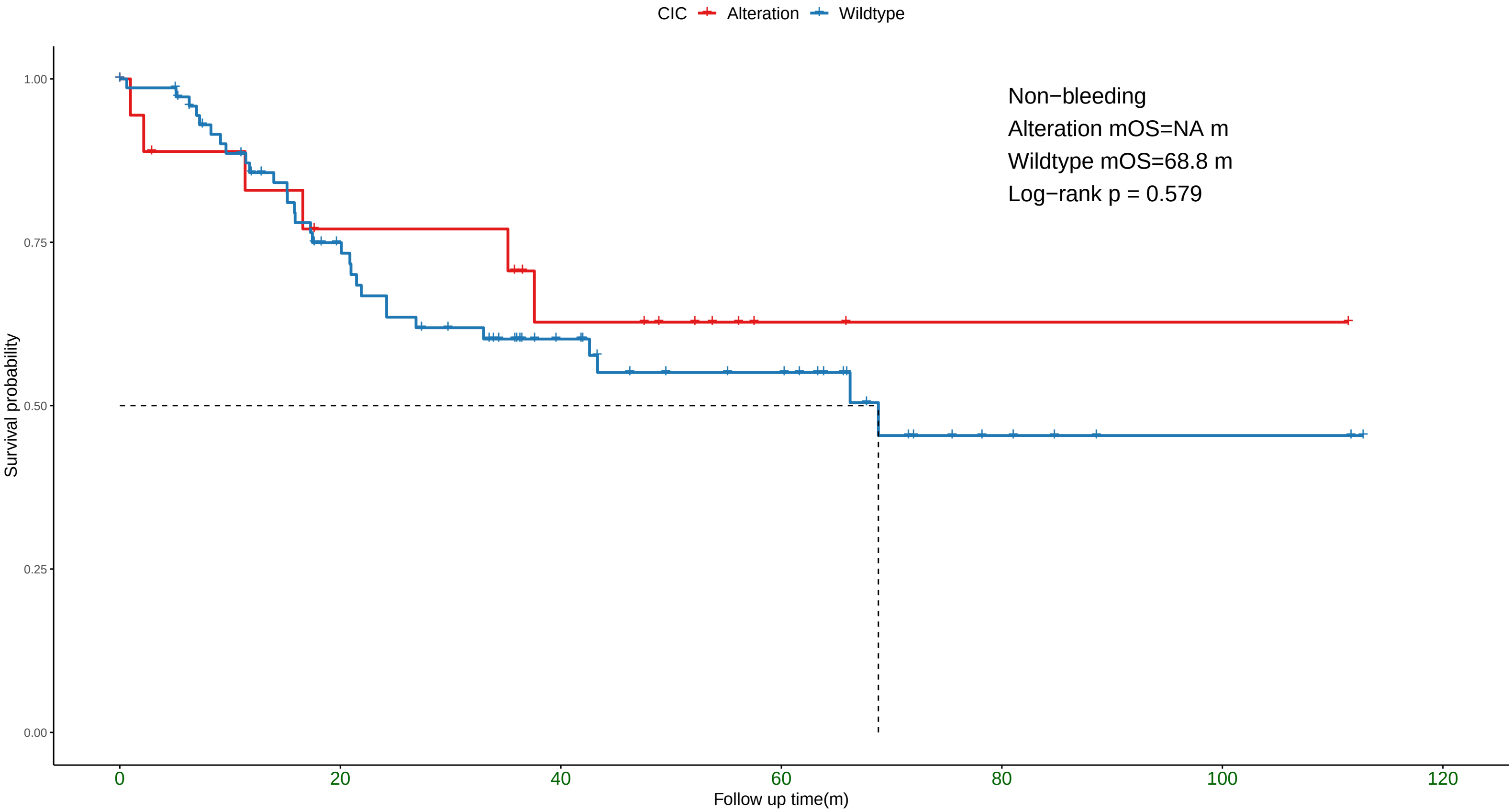
CIC
Alteration
Wildtype
Non−bleeding
Alteration mOS=NA m
Wildtype mOS=68.8 m
Log−rank p = 0.579
Survival probability
1.00
0.75
0.50
0.25
0.00
0
20
40
60
80
100
120
Follow up time(m)

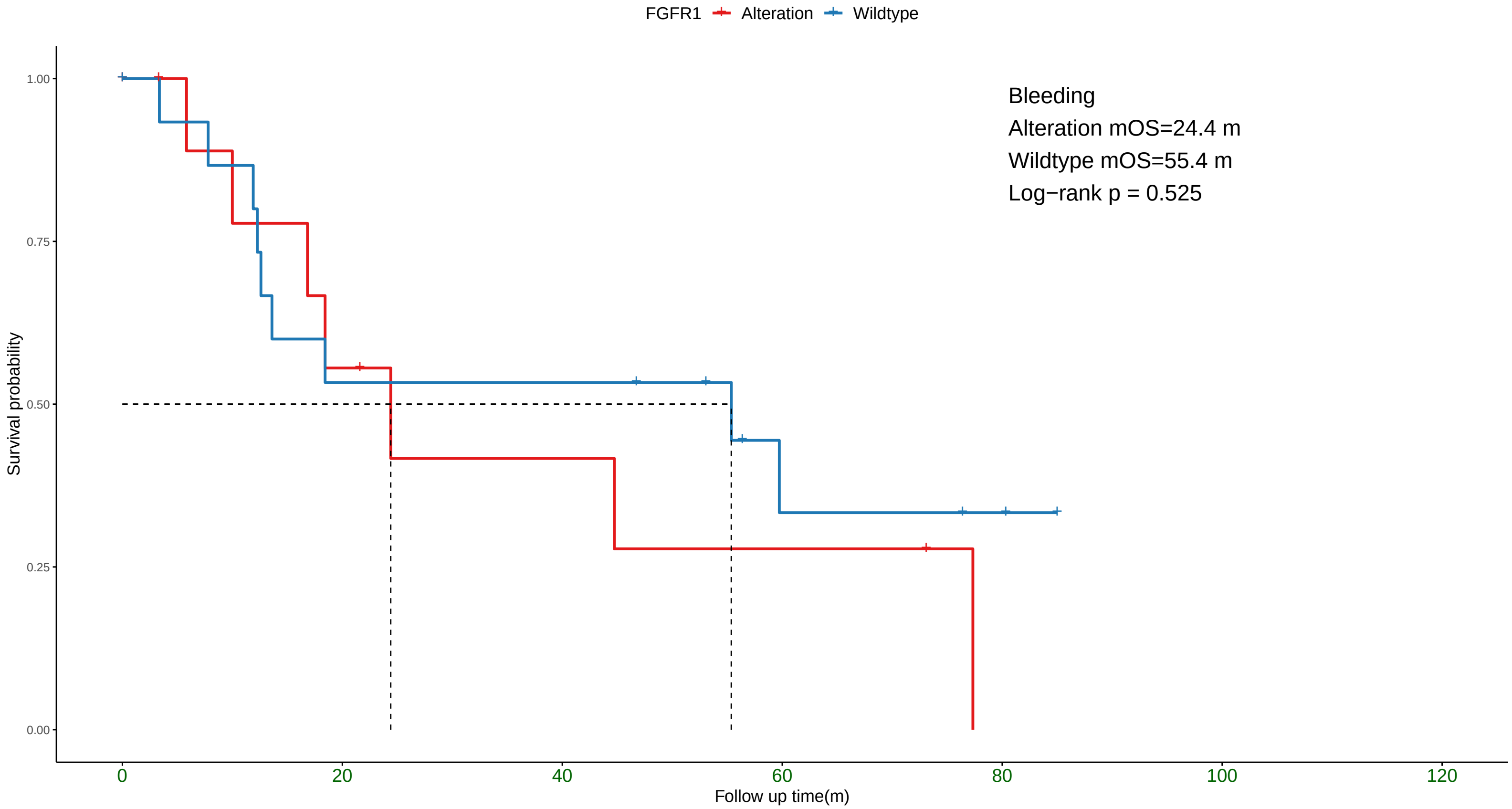
FGFR1
Alteration
Wildtype
Bleeding
Alteration mOS=24.4 m
Wildtype mOS=55.4 m
Log−rank p = 0.525
Survival probability
1.00
0.75
0.50
0.25
0.00
0
20
40
60
80
100
120
Follow up time(m)

-10-

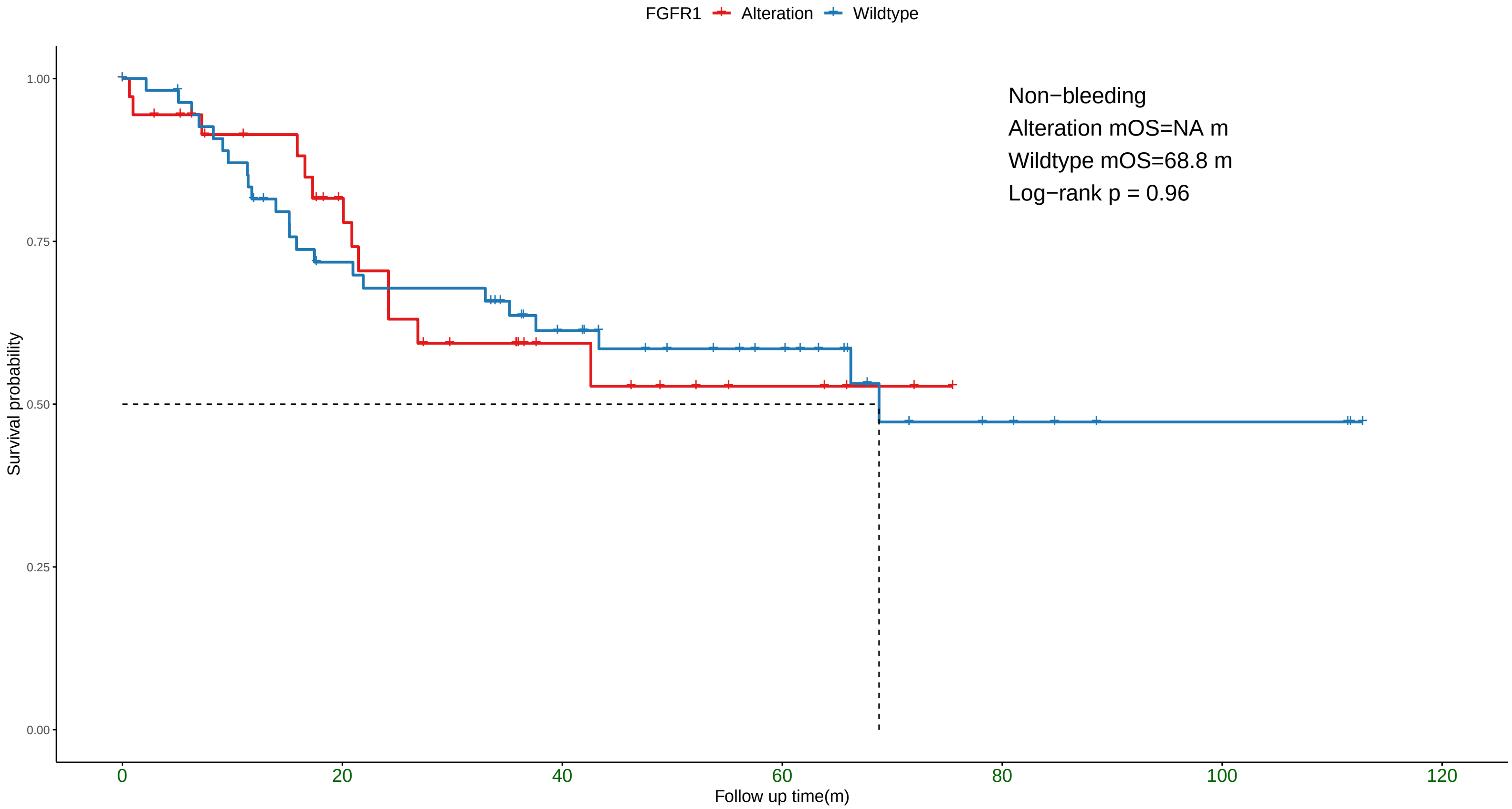
FGFR1 Alteration Wildtype
Non−bleeding
Alteration mOS=NA m
Wildtype mOS=68.8 m
Log−rank p = 0.96
Survival probability
1.00
0.75
0.50
0.25
0.00
0
20
40
60
80
100
120
Follow up time(m)

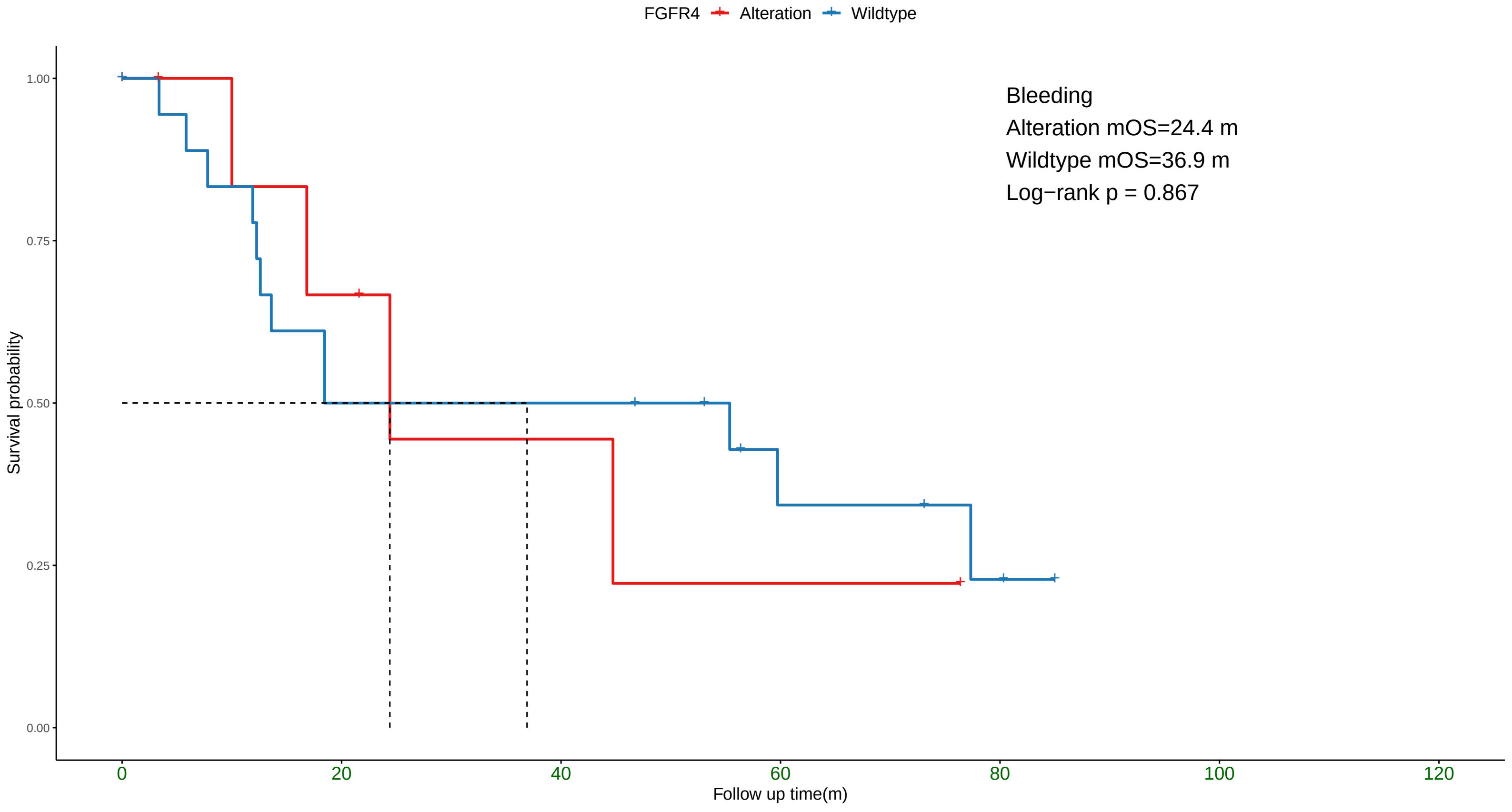
FGFR4
Alteration
Wildtype
Bleeding
Alteration mOS=24.4 m
Wildtype mOS=36.9 m
Log−rank p = 0.867
Survival probability
1.00
0.75
0.50
0.25
0.00
0
20
40
60
80
100
120
Follow up time(m)

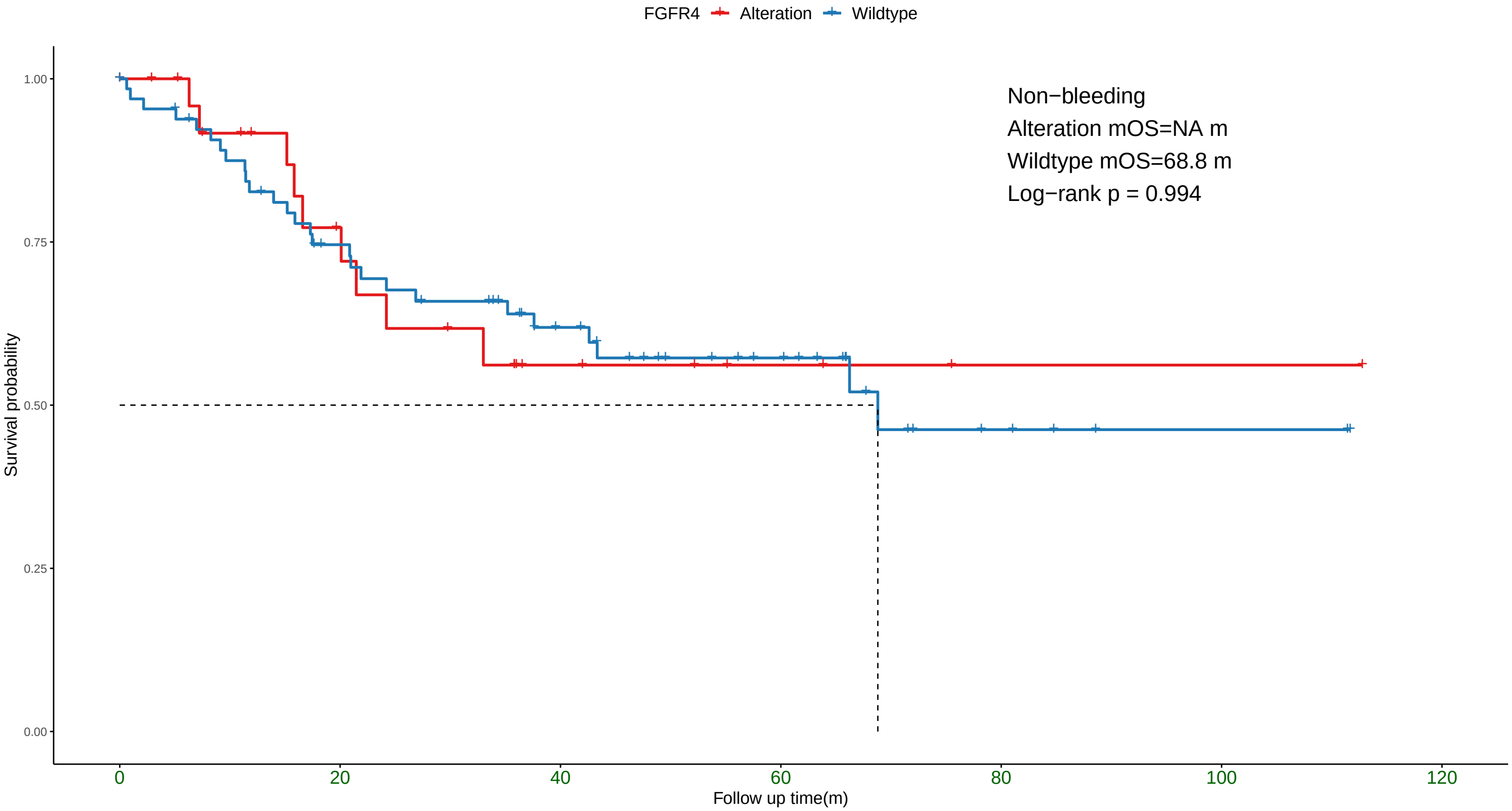
FGFR4 Alteration Wildtype
Non−bleeding
Alteration mOS=NA m
Wildtype mOS=68.8 m
Log−rank p = 0.994
Survival probability
1.00
0.75
0.50
0.25
0.00
0
20
40
60
80
100
120
Follow up time(m)

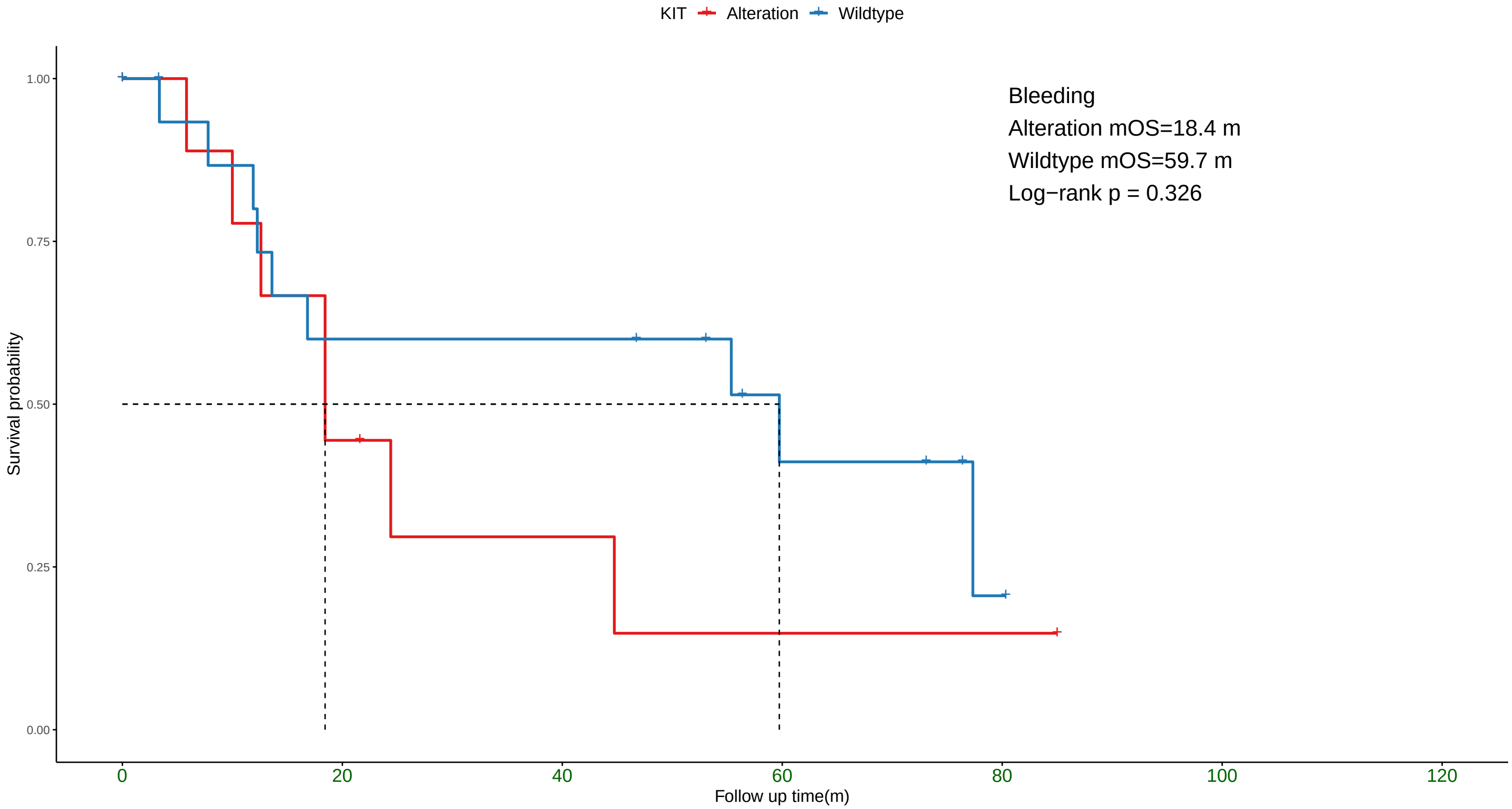
KIT
Alteration
Wildtype
Bleeding
Alteration mOS=18.4 m
Wildtype mOS=59.7 m
Log−rank p = 0.326
Survival probability
1.00
0.75
0.50
0.25
0.00
0
20
40
60
80
100
120
Follow up time(m)

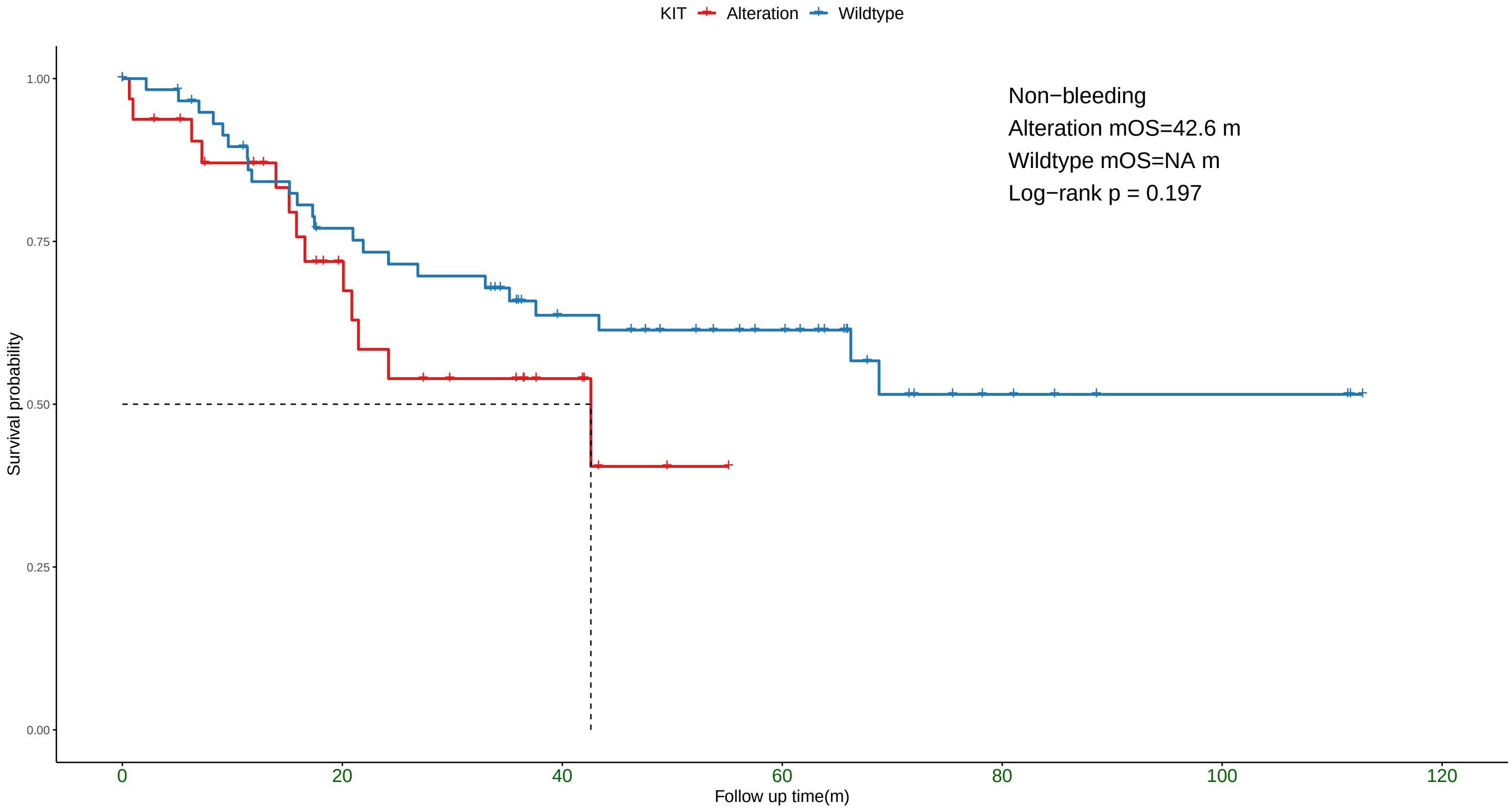
KIT
Alteration
Wildtype
Non−bleeding
Alteration mOS=42.6 m
Wildtype mOS=NA m
Log−rank p = 0.197
1.00
0.75
0.50
0.25
0.00
Survival probability
0
20
40
60
80
100
120
Follow up time(m)

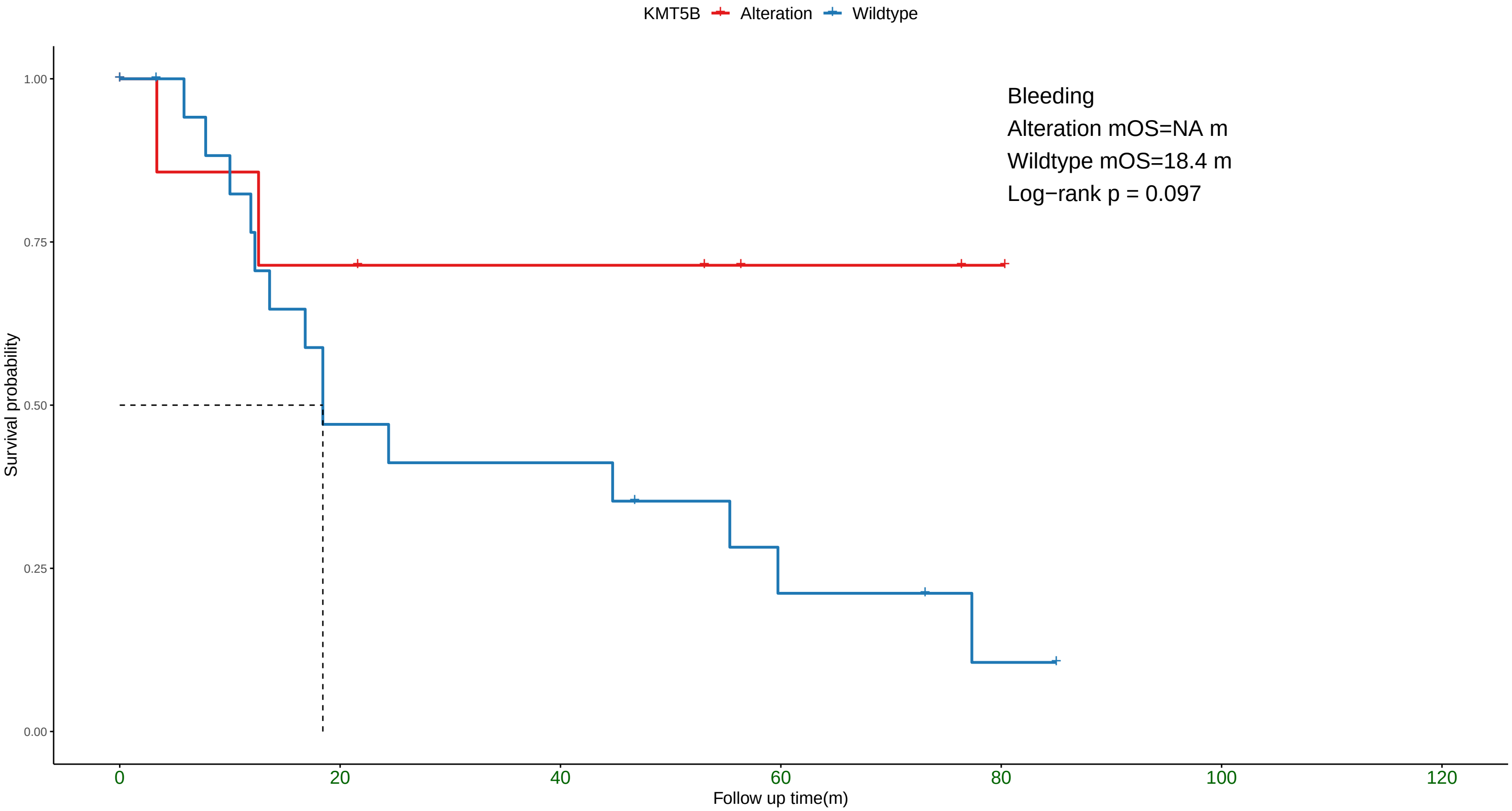
KMT5B
Alteration
Wildtype
Bleeding
Alteration mOS=NA m
Wildtype mOS=18.4 m
Log−rank p = 0.097
Survival probability
1.00
0.75
0.50
0.25
0.00
0
20
40
60
80
100
120
Follow up time(m)

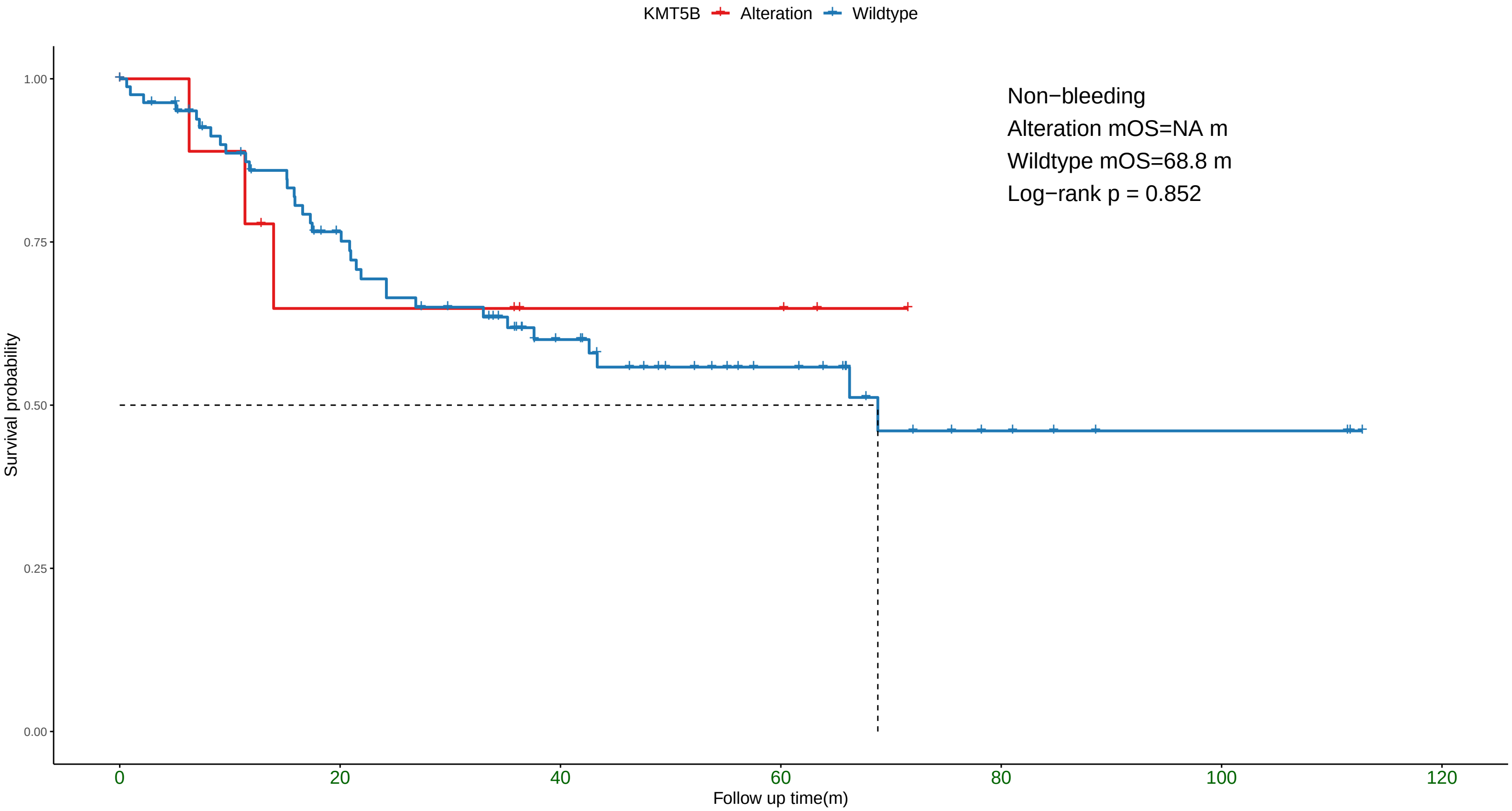
KMT5B
Alteration
Wildtype
Non−bleeding
Alteration mOS=NA m
Wildtype mOS=68.8 m
Log−rank p = 0.852
Survival probability
1.00
0.75
0.50
0.25
0.00
0
20
40
60
80
100
120
Follow up time(m)

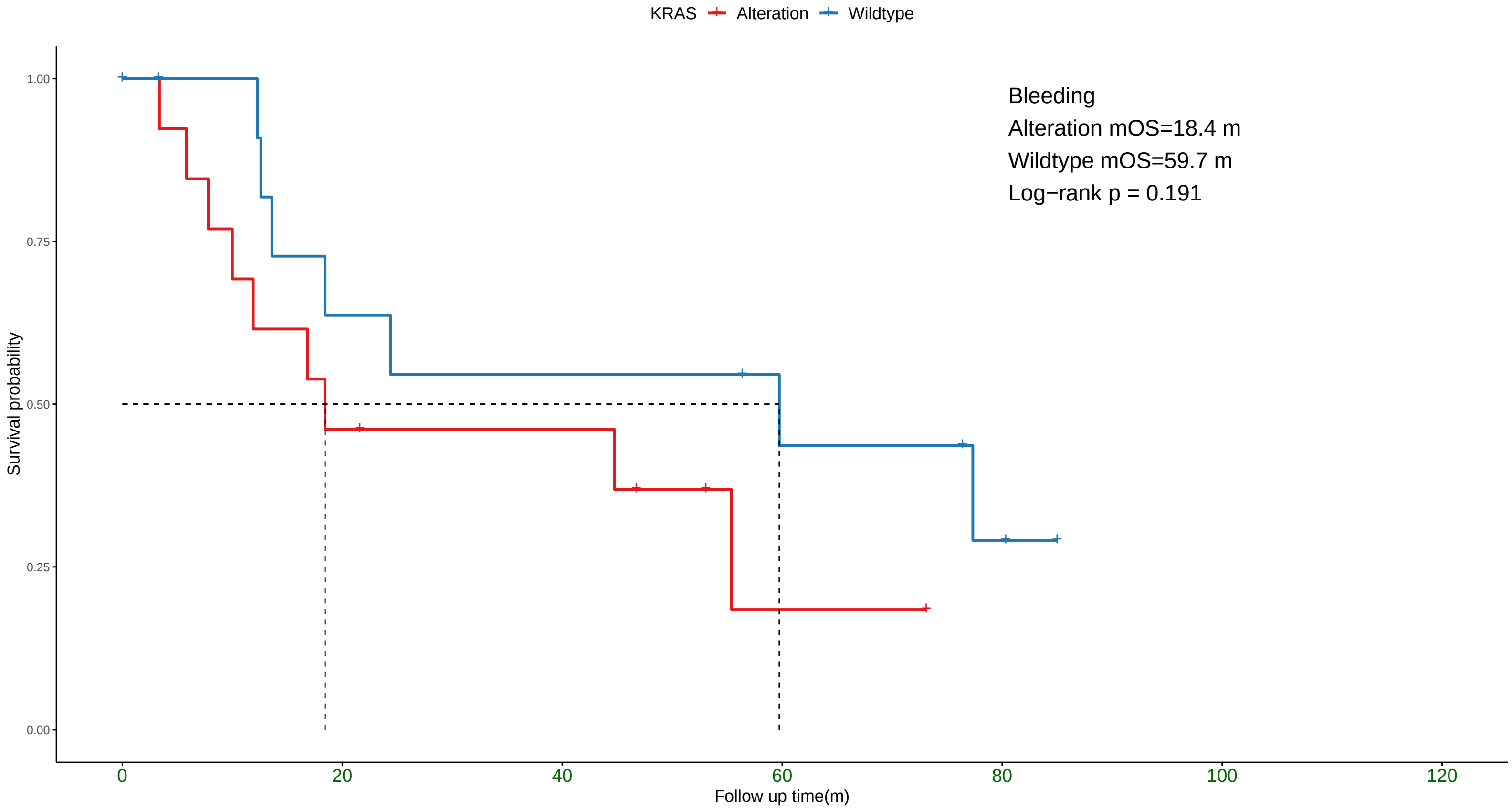
KRAS
Alteration
Wildtype
Bleeding
Alteration mOS=18.4 m
Wildtype mOS=59.7 m
Log−rank p = 0.191
Survival probability
1.00
0.75
0.50
0.25
0.00
0
20
40
60
80
100
120
Follow up time(m)

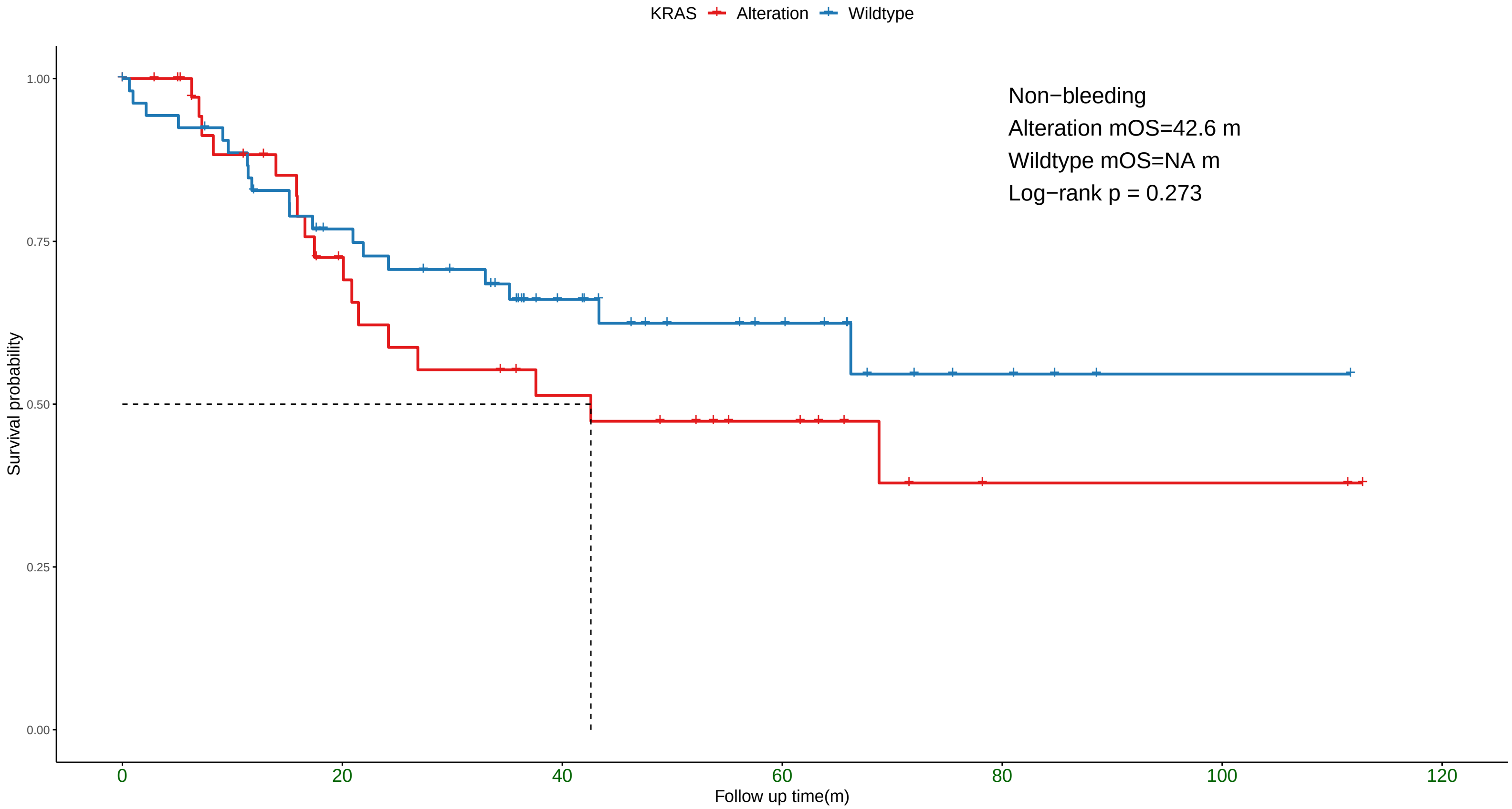
KRAS
Alteration
Wildtype
Non−bleeding
Alteration mOS=42.6 m
Wildtype mOS=NA m
Log−rank p = 0.273
Survival probability
1.00
0.75
0.50
0.25
0.00
0
20
40
60
80
100
120
Follow up time(m)

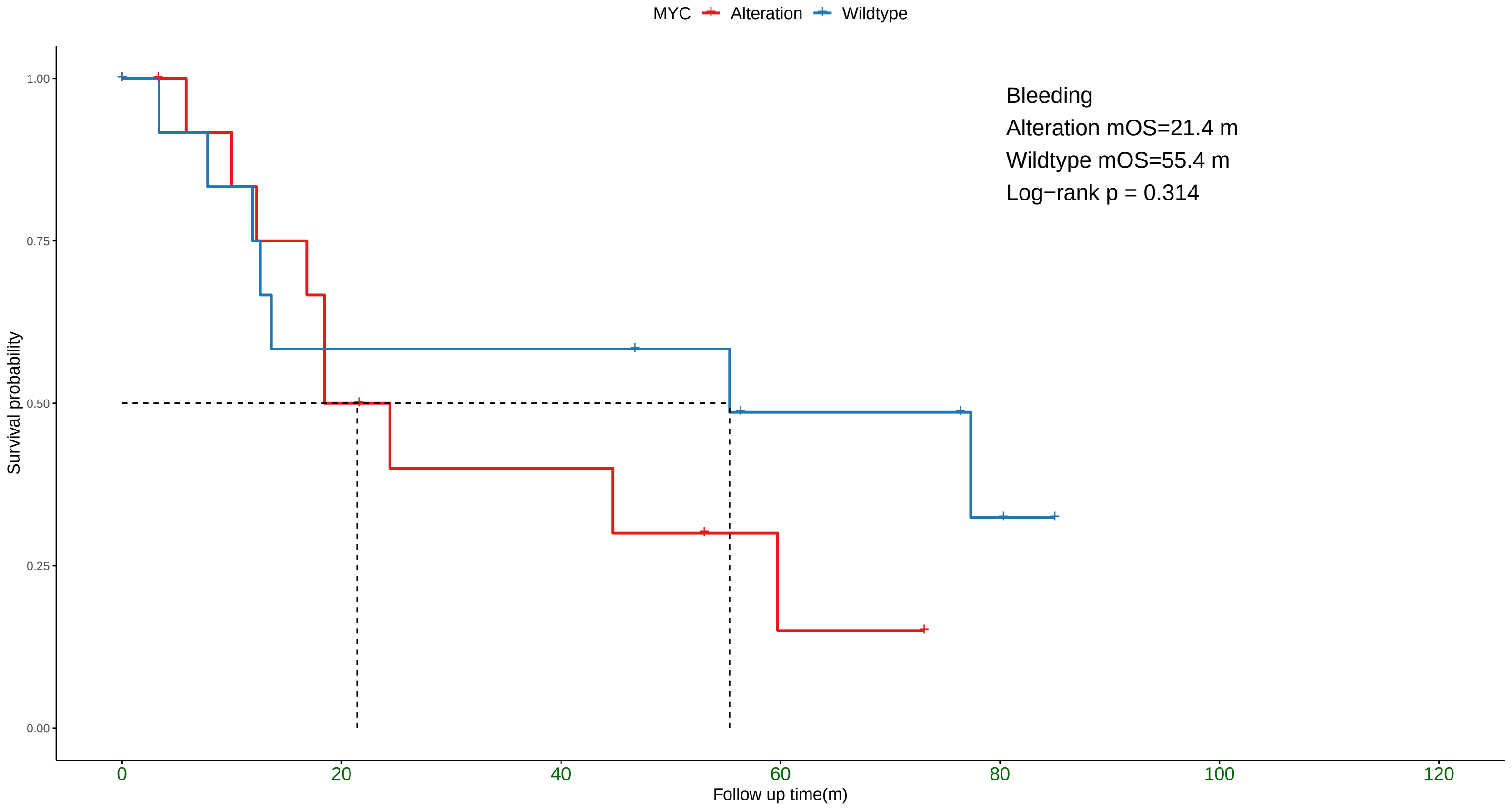
MYC
Alteration
Wildtype
Bleeding
Alteration mOS=21.4 m
Wildtype mOS=55.4 m
Log−rank p = 0.314
Survival probability
1.00
0.75
0.50
0.25
0.00
0
20
40
60
80
100
120
Follow up time(m)

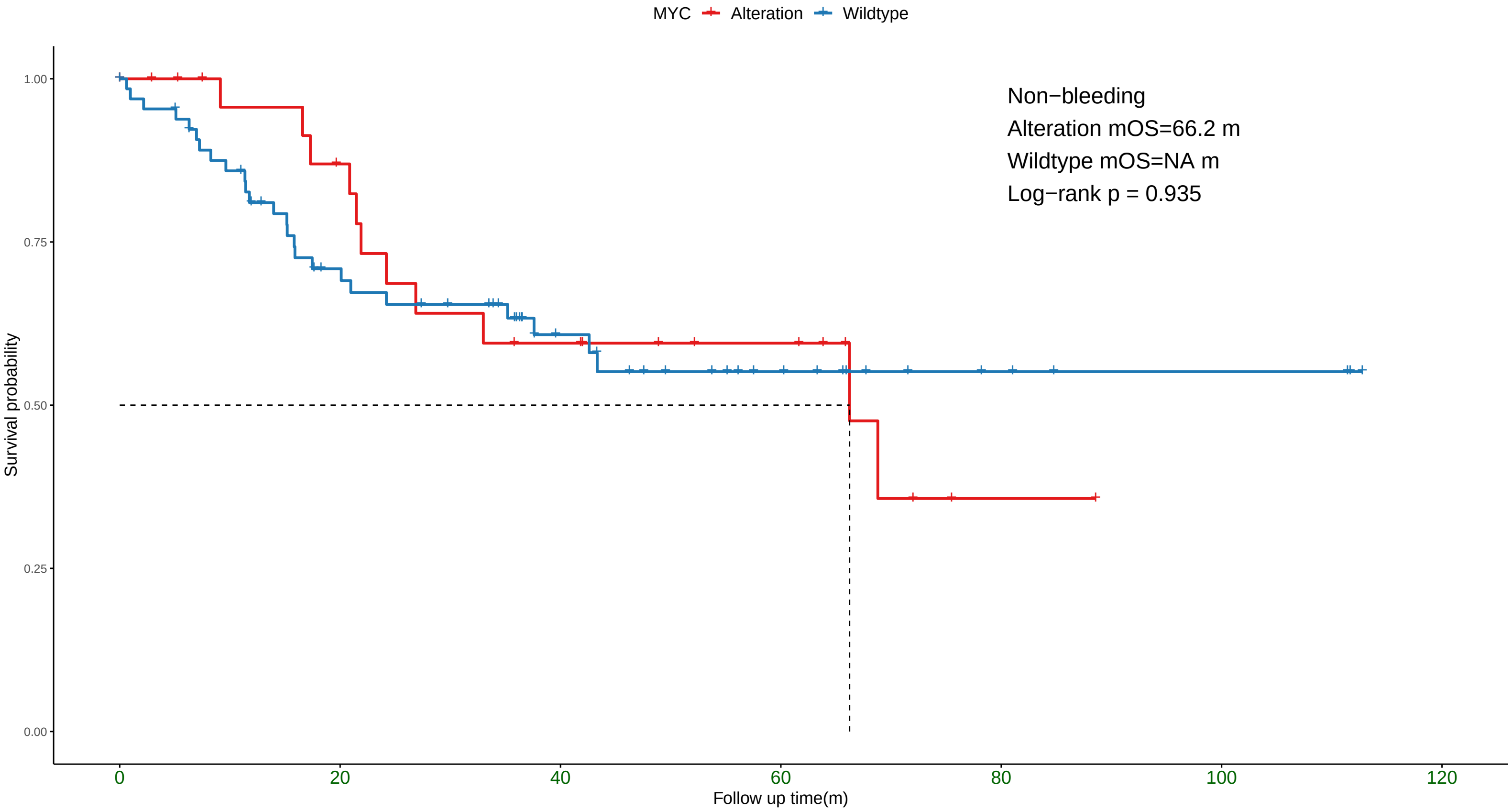
MYC Alteration Wildtype
Non−bleeding
Alteration mOS=66.2 m
Wildtype mOS=NA m
Log−rank p = 0.935
Survival probability
1.00
0.75
0.50
0.25
0.00
0
20
40
60
80
100
120
Follow up time(m)

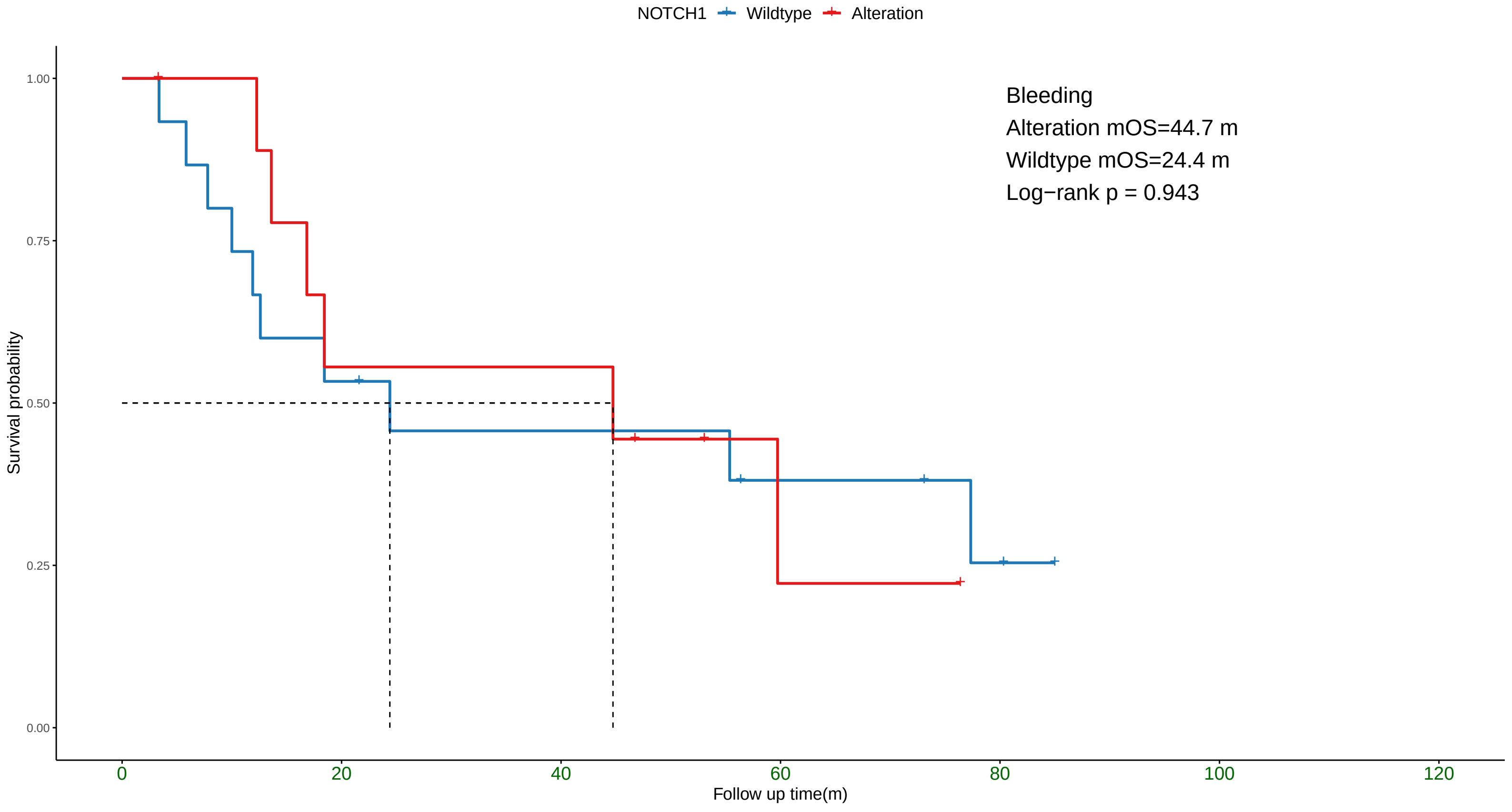
NOTCH1 Wildtype Alteration
Bleeding
Alteration mOS=44.7 m
Wildtype mOS=24.4 m
Log−rank p = 0.943
Survival probability
1.00
0.75
0.50
0.25
0.00
0
20
40
60
80
100
120
Follow up time(m)

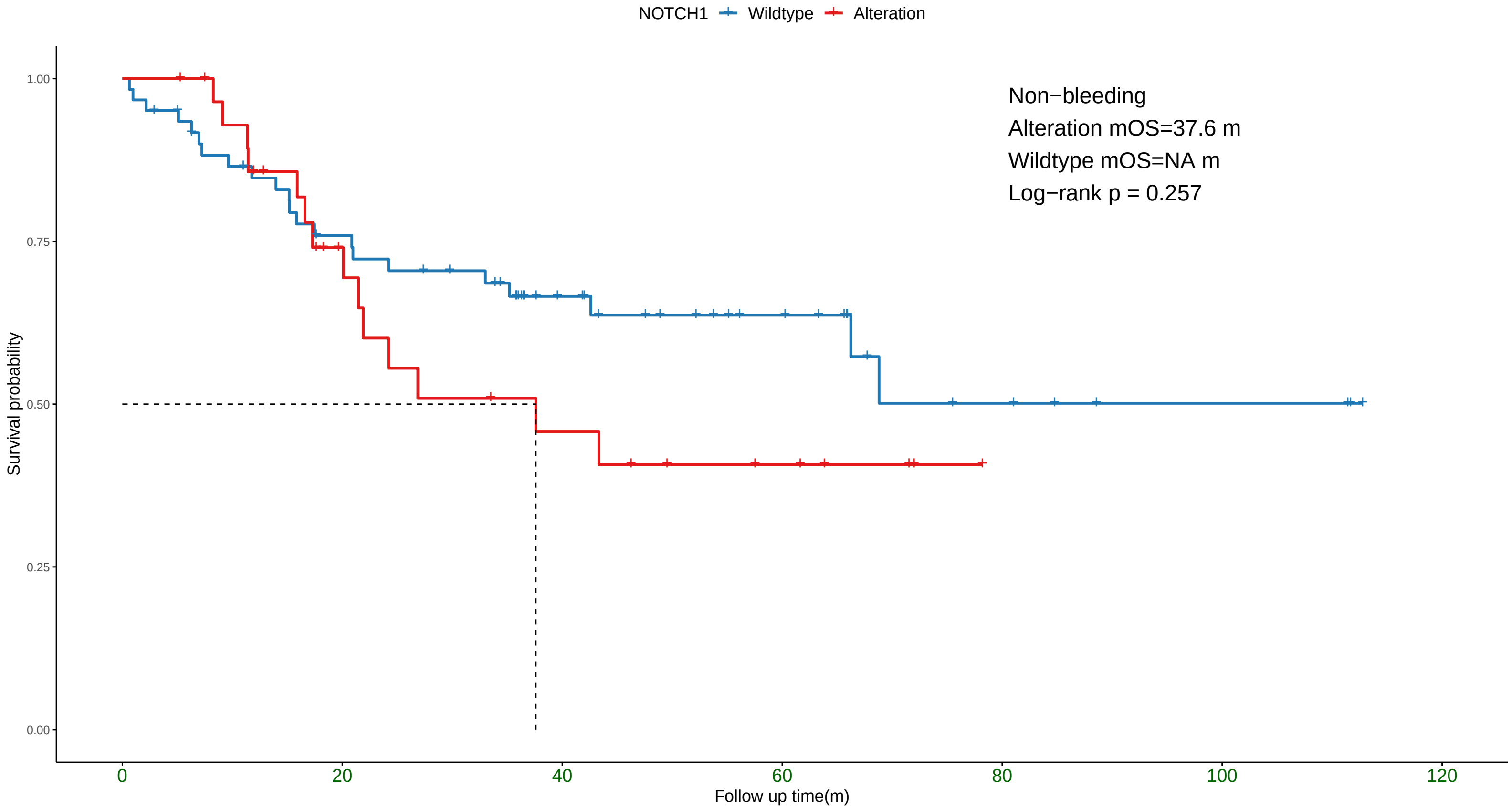
NOTCH1 Wildtype Alteration
Non−bleeding
Alteration mOS=37.6 m
Wildtype mOS=NA m
Log−rank p = 0.257
Survival probability
1.00
0.75
0.50
0.25
0.00
0
20
40
60
80
100
120
Follow up time(m)

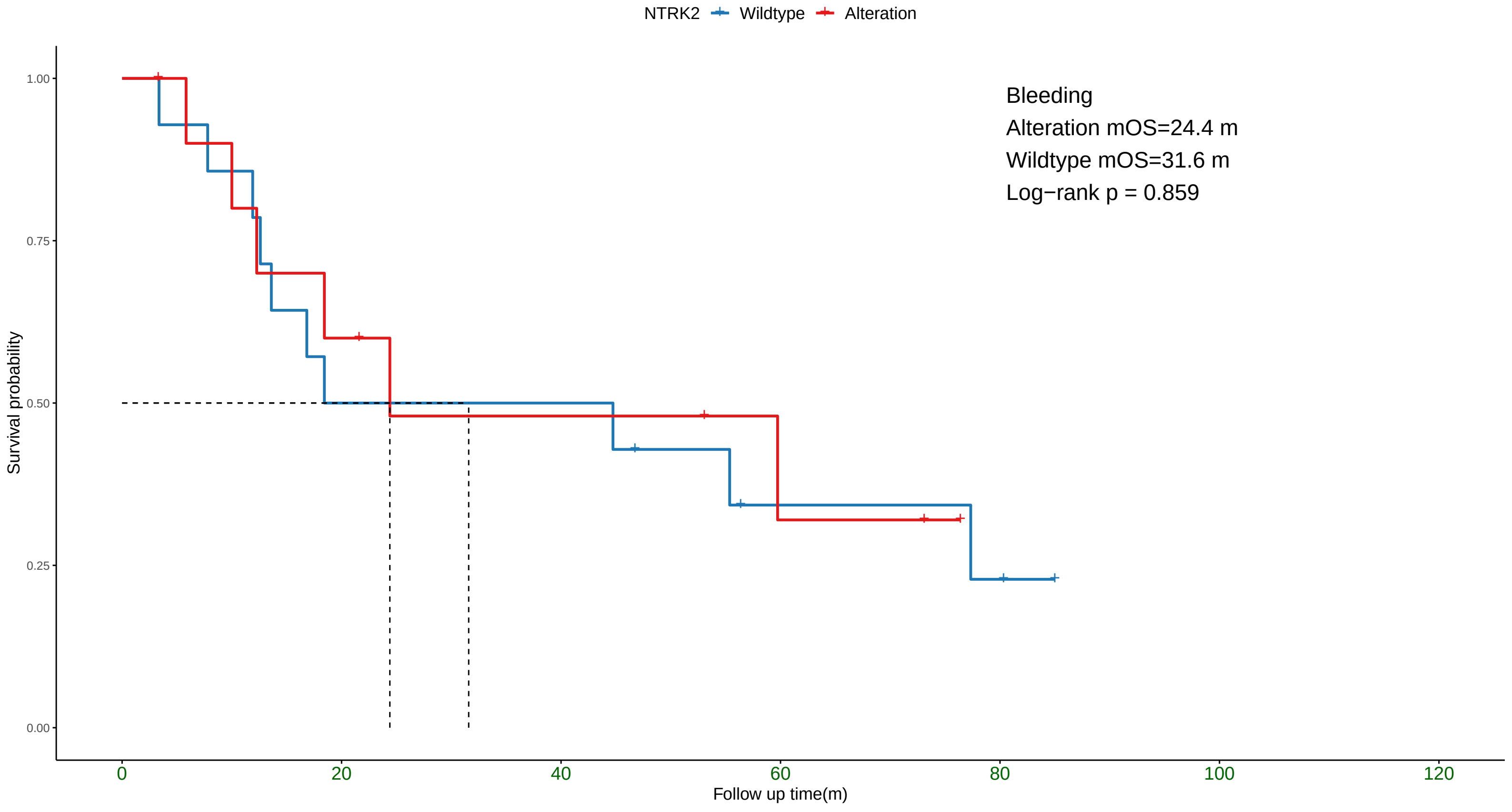
NTRK2 Wildtype Alteration
Bleeding
Alteration mOS=24.4 m
Wildtype mOS=31.6 m
Log−rank p = 0.859
Survival probability
1.00
0.75
0.50
0.25
0.00
0
20
40
60
80
100
120
Follow up time(m)

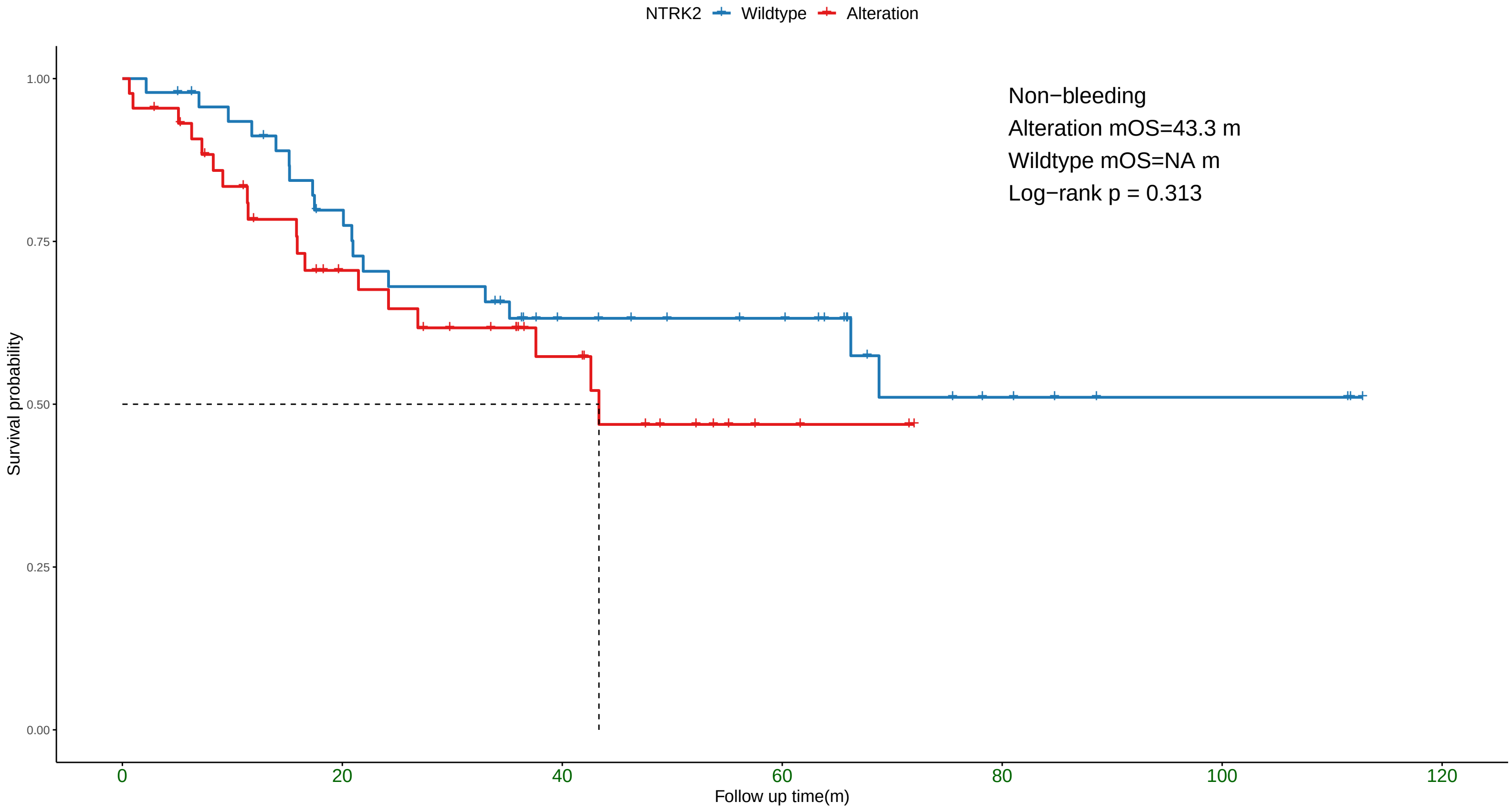
NTRK2 Wildtype Alteration
Non−bleeding
Alteration mOS=43.3 m
Wildtype mOS=NA m
Log−rank p = 0.313
Survival probability
1.00
0.75
0.50
0.25
0.00
0
20
40
60
80
100
120
Follow up time(m)

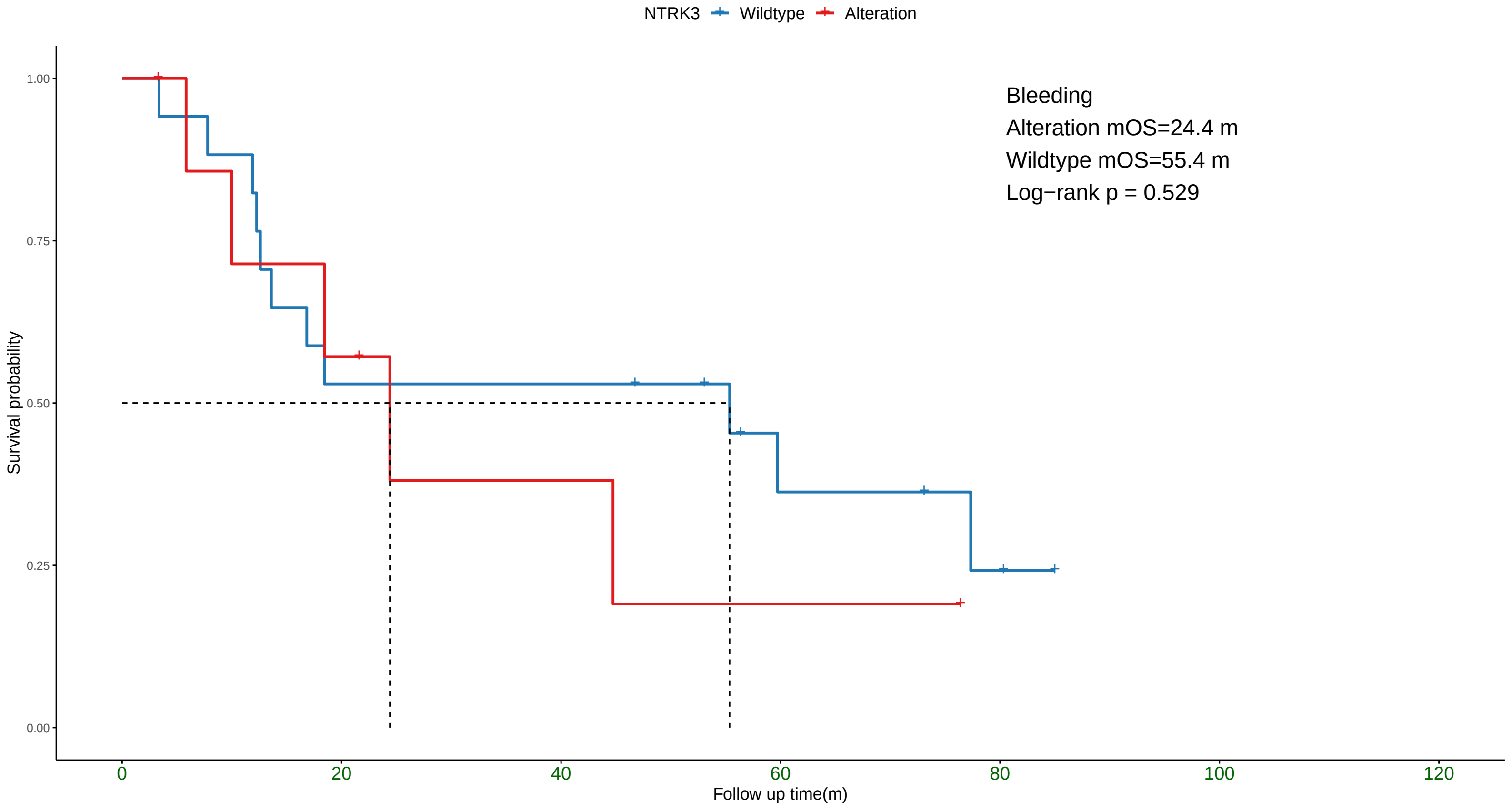
NTRK3 Wildtype Alteration
Bleeding
Alteration mOS=24.4 m
Wildtype mOS=55.4 m
Log−rank p = 0.529
Survival probability
1.00
0.75
0.50
0.25
0.00
0
20
40
60
80
100
120
Follow up time(m)

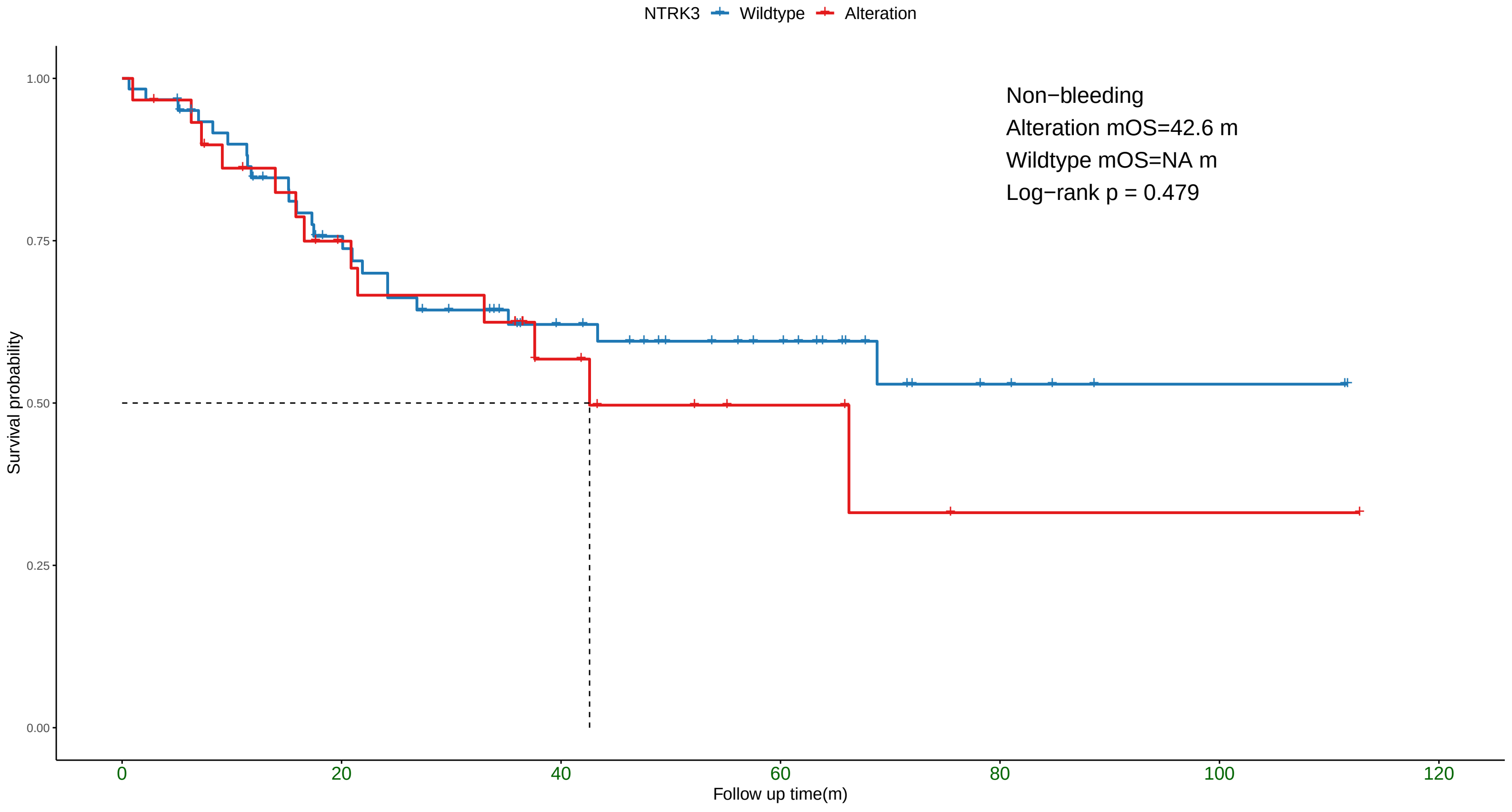
NTRK3 Wildtype Alteration
Non−bleeding
Alteration mOS=42.6 m
Wildtype mOS=NA m
Log−rank p = 0.479
Survival probability
1.00
0.75
0.50
0.25
0.00
0
20
40
60
80
100
120
Follow up time(m)

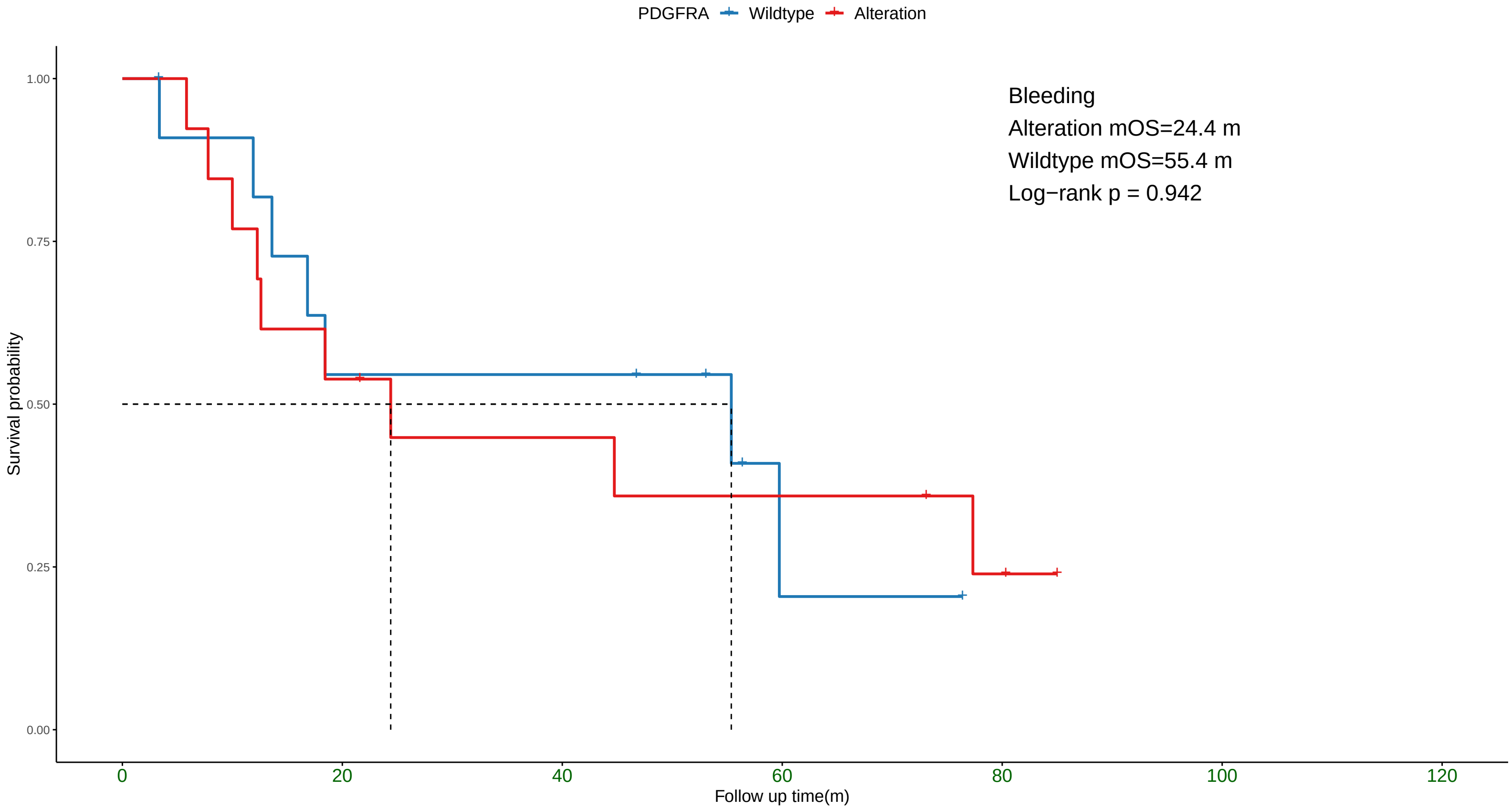
PDGFRA
Wildtype
Alteration
Bleeding
Alteration mOS=24.4 m
Wildtype mOS=55.4 m
Log−rank p = 0.942
Survival probability
1.00
0.75
0.50
0.25
0.00
0
20
40
60
80
100
120
Follow up time(m)

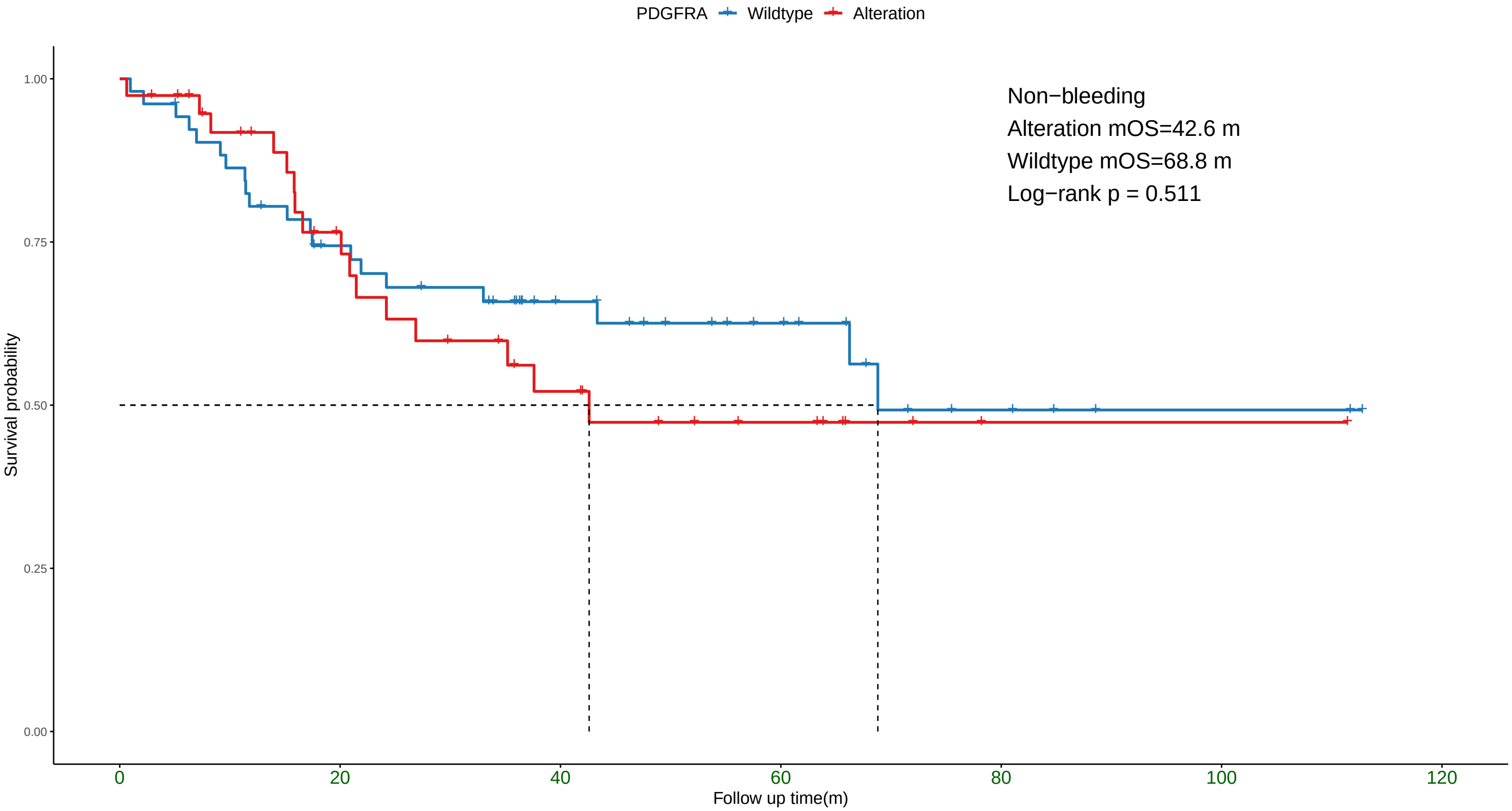
PDGFRA
Wildtype
Alteration
Non−bleeding
Alteration mOS=42.6 m
Wildtype mOS=68.8 m
Log−rank p = 0.511
Survival probability
1.00
0.75
0.50
0.25
0.00
0
20
40
60
80
100
120
Follow up time(m)

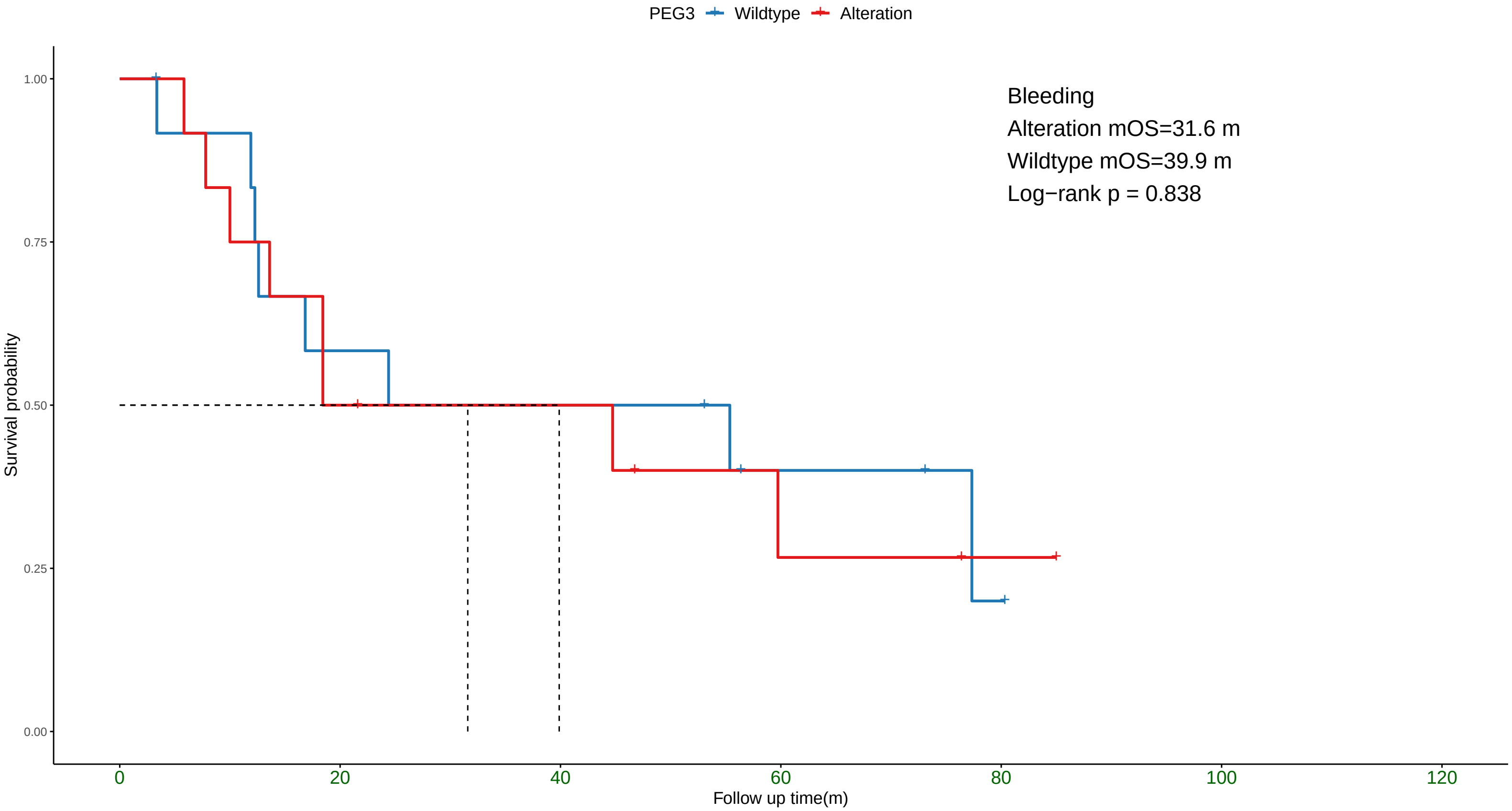
PEG3
Wildtype
Alteration
Bleeding
Alteration mOS=31.6 m
Wildtype mOS=39.9 m
Log−rank p = 0.838
Survival probability
1.00
0.75
0.50
0.25
0.00
0
20
40
60
80
100
120
Follow up time(m)

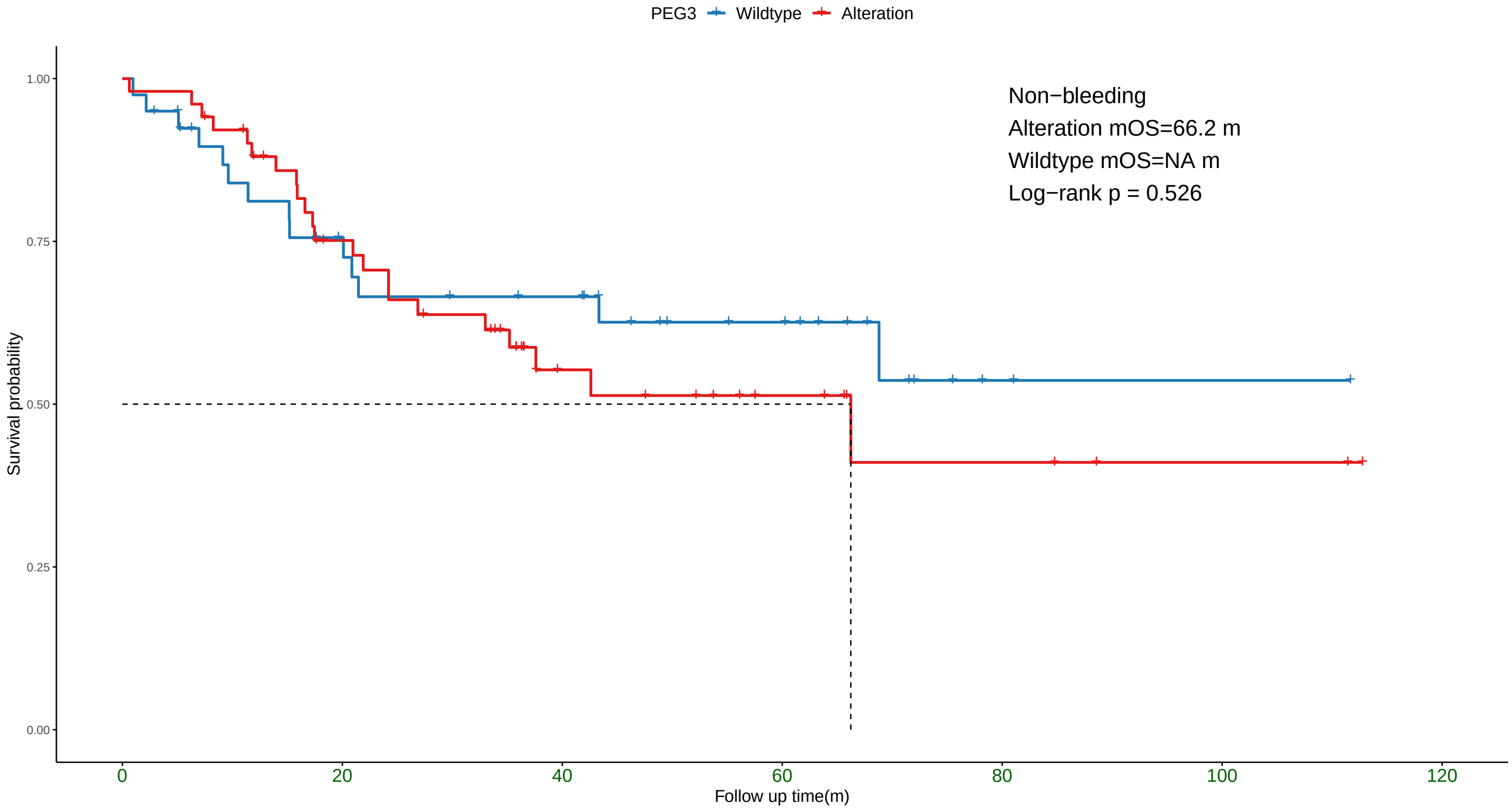
PEG3
Wildtype
Alteration
Non−bleeding
Alteration mOS=66.2 m
Wildtype mOS=NA m
Log−rank p = 0.526
1.00
0.75
0.50
0.25
0.00
Survival probability
0
20
40
60
80
100
120
Follow up time(m)

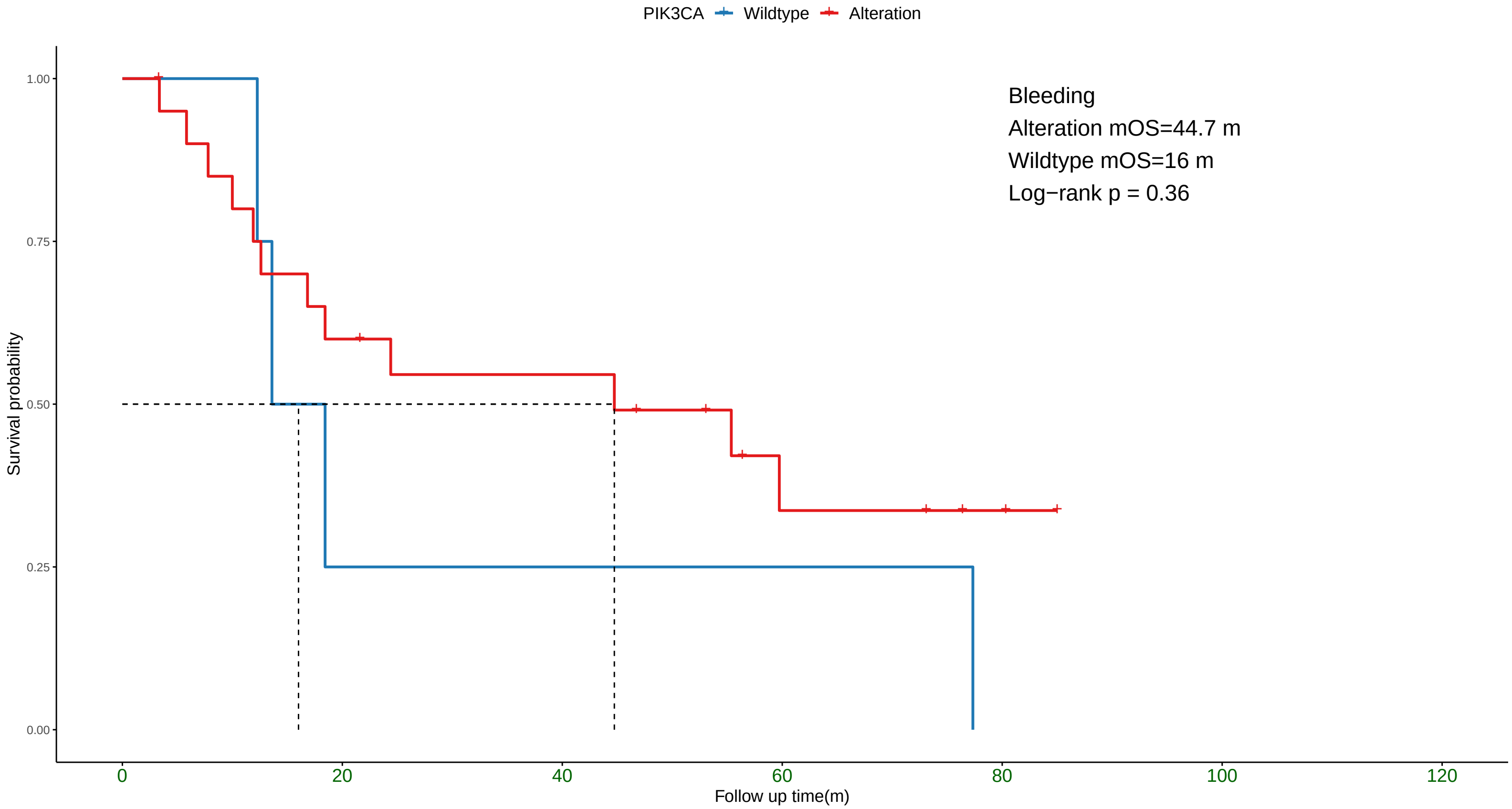
PIK3CA Wildtype Alteration
Bleeding
Alteration mOS=44.7 m
Wildtype mOS=16 m
Log−rank p = 0.36
Survival probability
1.00
0.75
0.50
0.25
0.00
0
20
40
60
80
100
120
Follow up time(m)

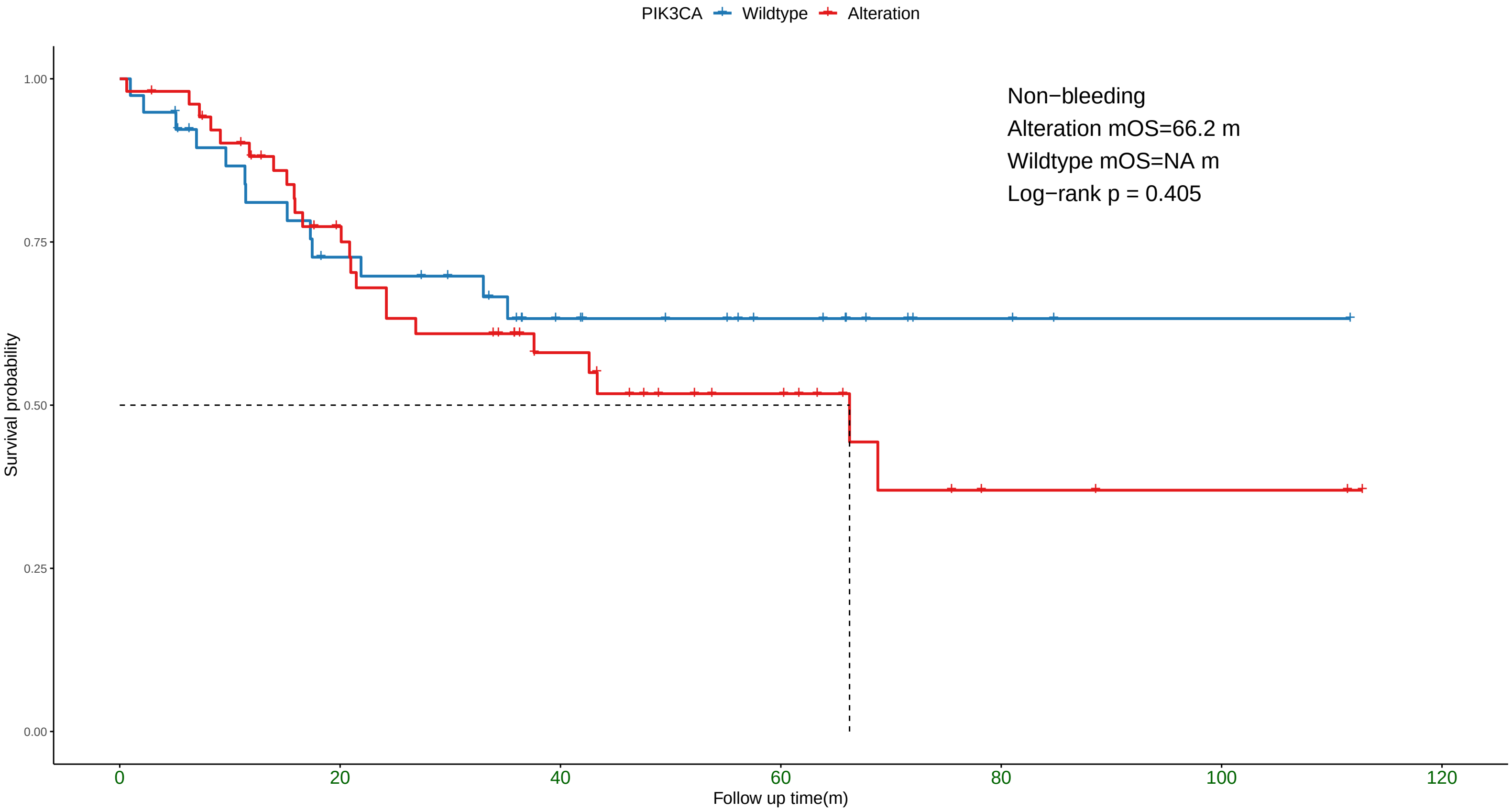
PIK3CA
Wildtype
Alteration
Non−bleeding
Alteration mOS=66.2 m
Wildtype mOS=NA m
Log−rank p = 0.405
Survival probability
1.00
0.75
0.50
0.25
0.00
0
20
40
60
80
100
120
Follow up time(m)

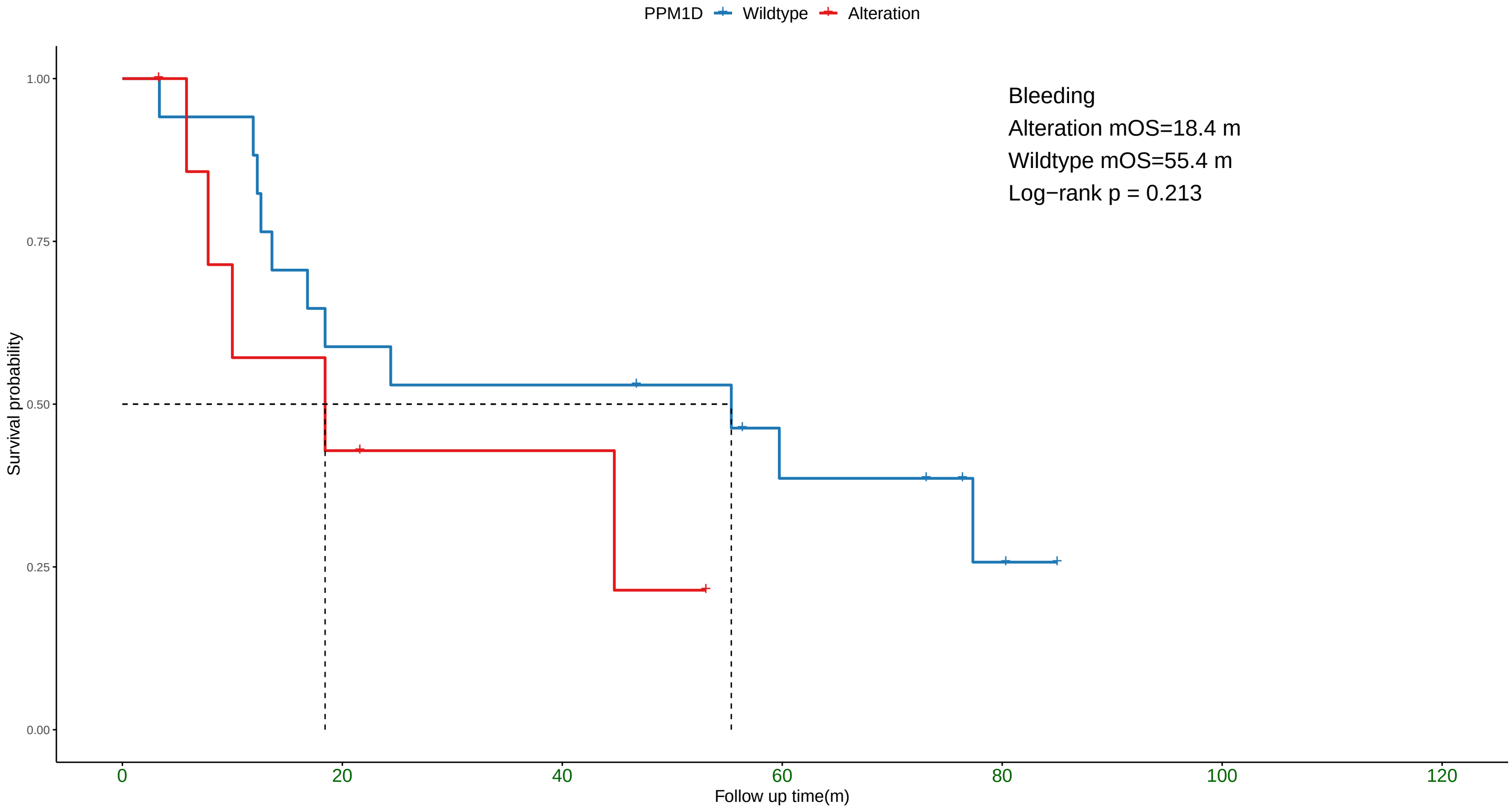
PPM1D Wildtype Alteration
Bleeding
Alteration mOS=18.4 m
Wildtype mOS=55.4 m
Log−rank p = 0.213
Survival probability
1.00
0.75
0.50
0.25
0.00
0
20
40
60
80
100
120
Follow up time(m)

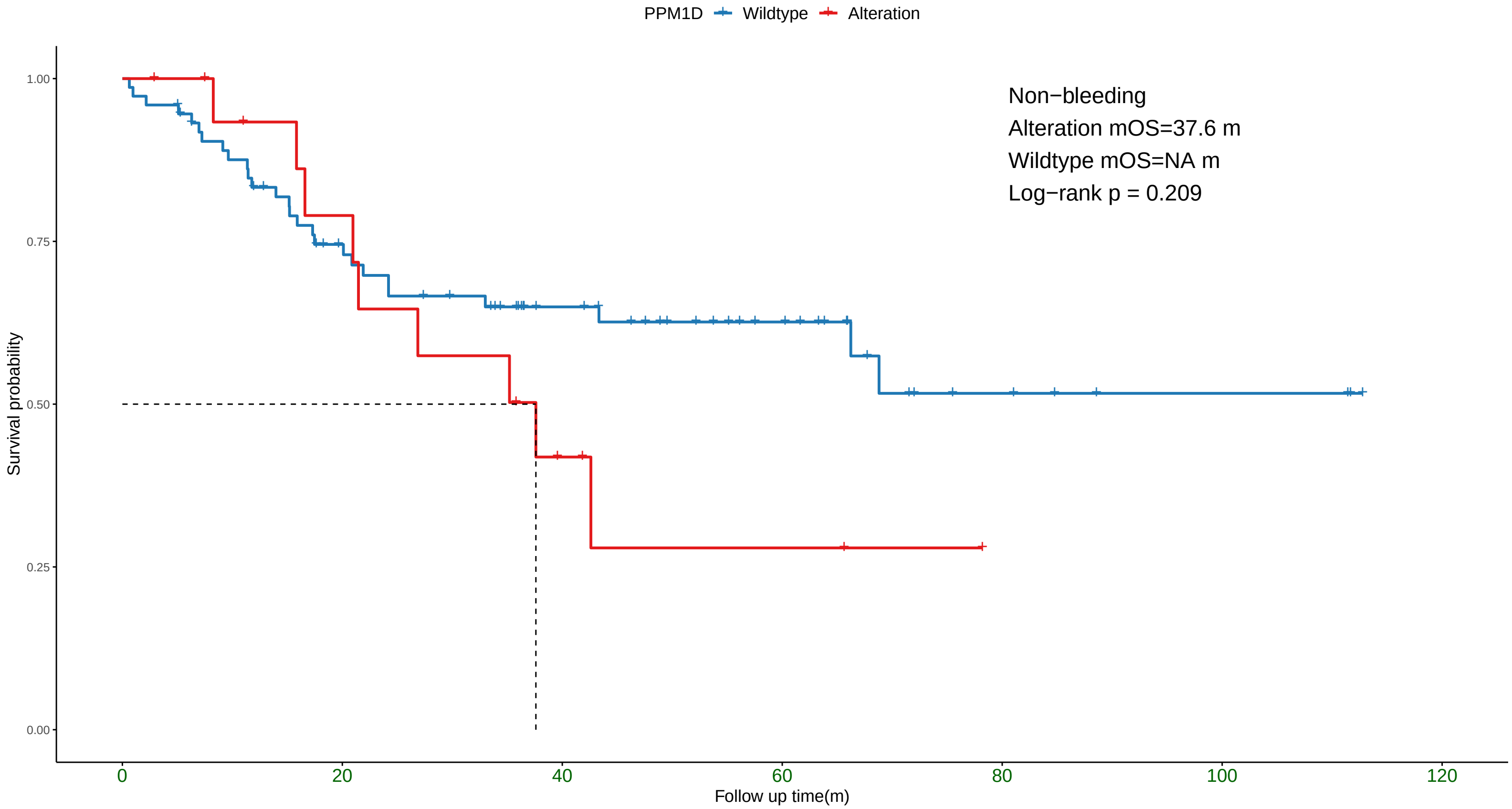
PPM1D
Wildtype
Alteration
Non−bleeding
Alteration mOS=37.6 m
Wildtype mOS=NA m
Log−rank p = 0.209
Survival probability
1.00
0.75
0.50
0.25
0.00
0
20
40
60
80
100
120
Follow up time(m)

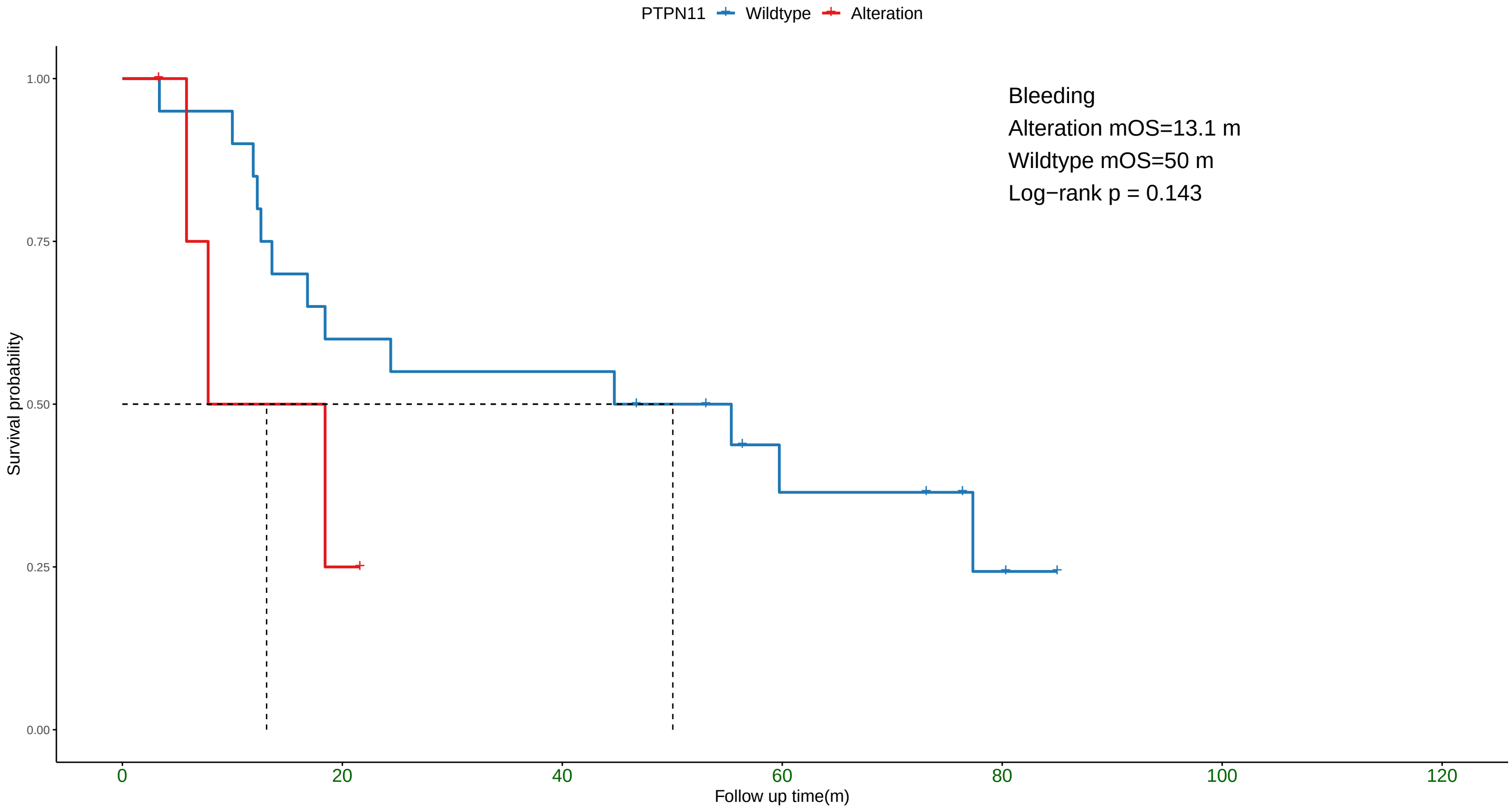
PTPN11
Wildtype
Alteration
Bleeding
Alteration mOS=13.1 m
Wildtype mOS=50 m
Log−rank p = 0.143
1.00
0.75
0.50
0.25
0.00
Survival probability
0
20
40
60
80
100
120
Follow up time(m)

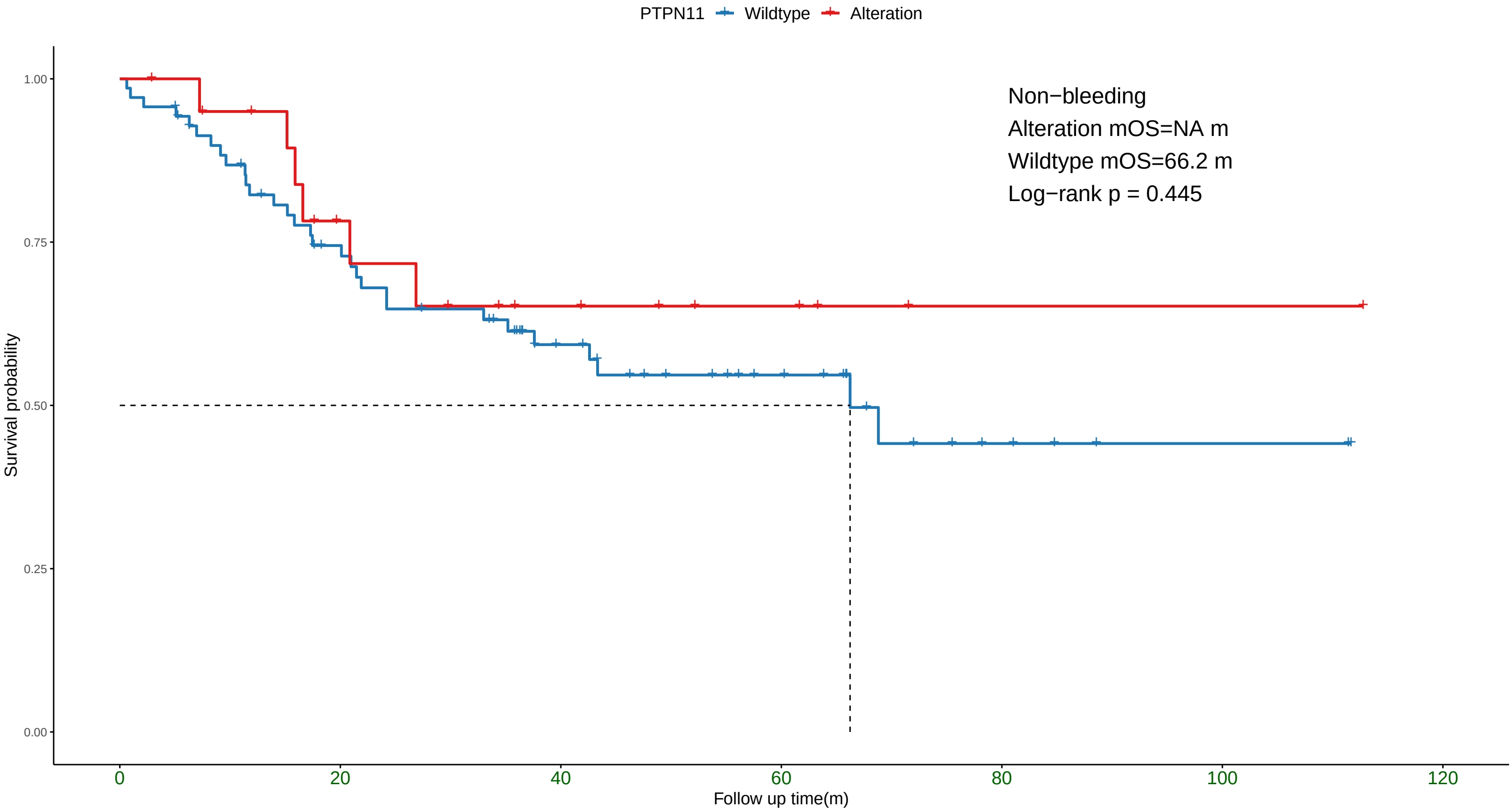
PTPN11 Wildtype Alteration
Non−bleeding
Alteration mOS=NA m
Wildtype mOS=66.2 m
Log−rank p = 0.445
Survival probability
1.00
0.75
0.50
0.25
0.00
0
20
40
60
80
100
120
Follow up time(m)

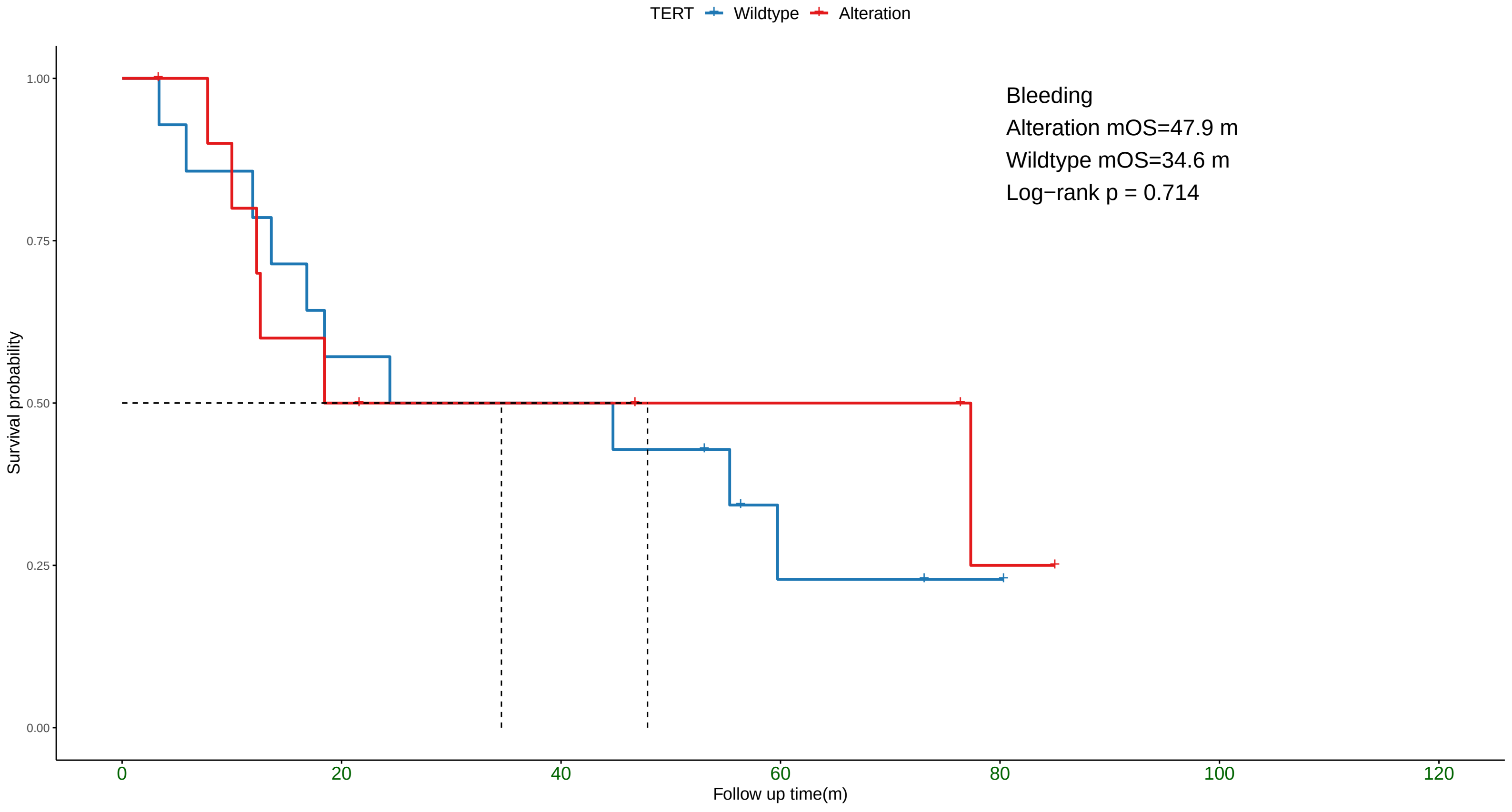
TERT
Wildtype
Alteration
Bleeding
Alteration mOS=47.9 m
Wildtype mOS=34.6 m
Log−rank p = 0.714
Survival probability
1.00
0.75
0.50
0.25
0.00
0
20
40
60
80
100
120
Follow up time(m)

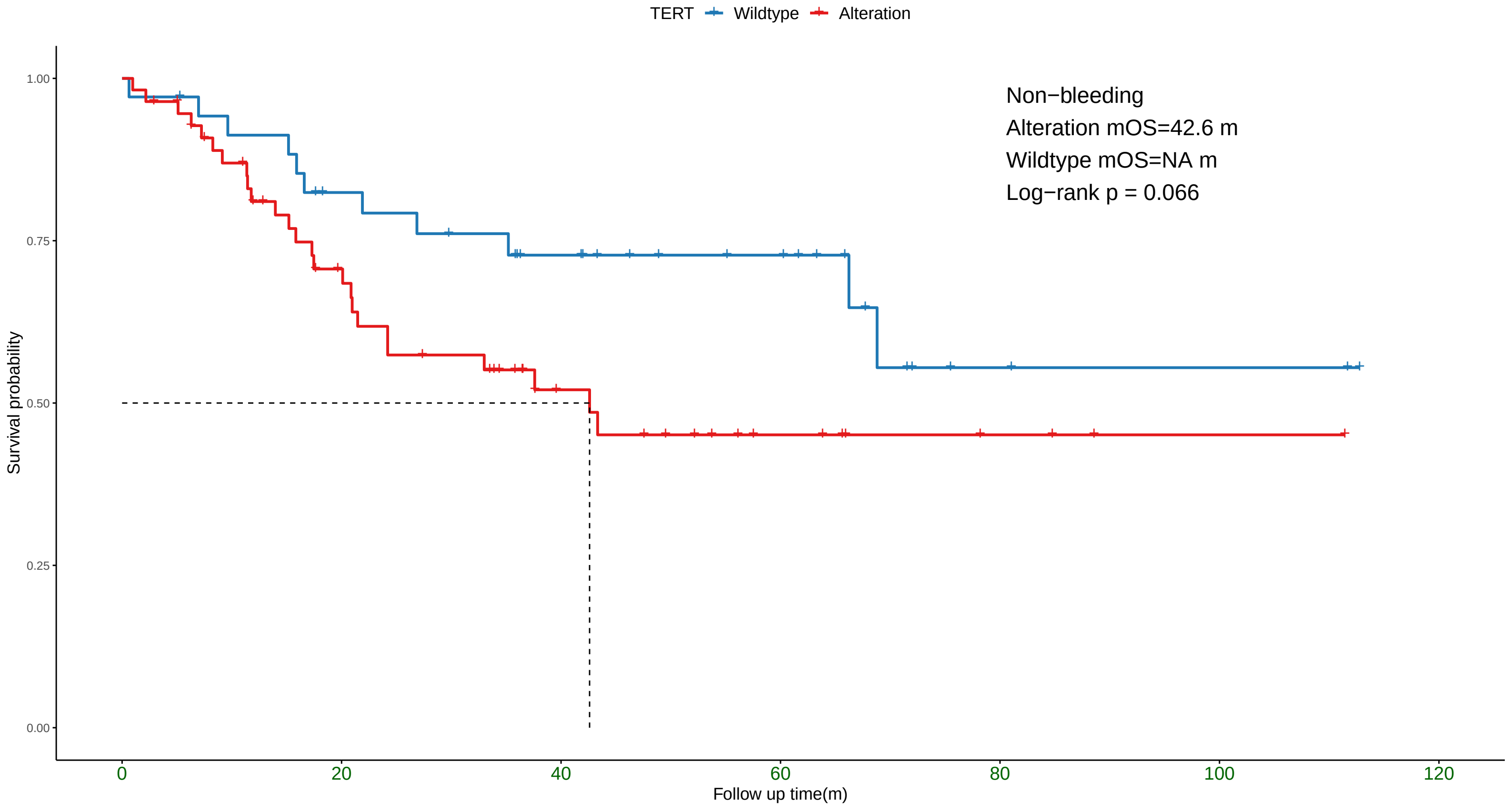
TERT
Wildtype
Alteration
Non−bleeding
Alteration mOS=42.6 m
Wildtype mOS=NA m
Log−rank p = 0.066
Survival probability
1.00
0.75
0.50
0.25
0.00
0
20
40
60
80
100
120
Follow up time(m)

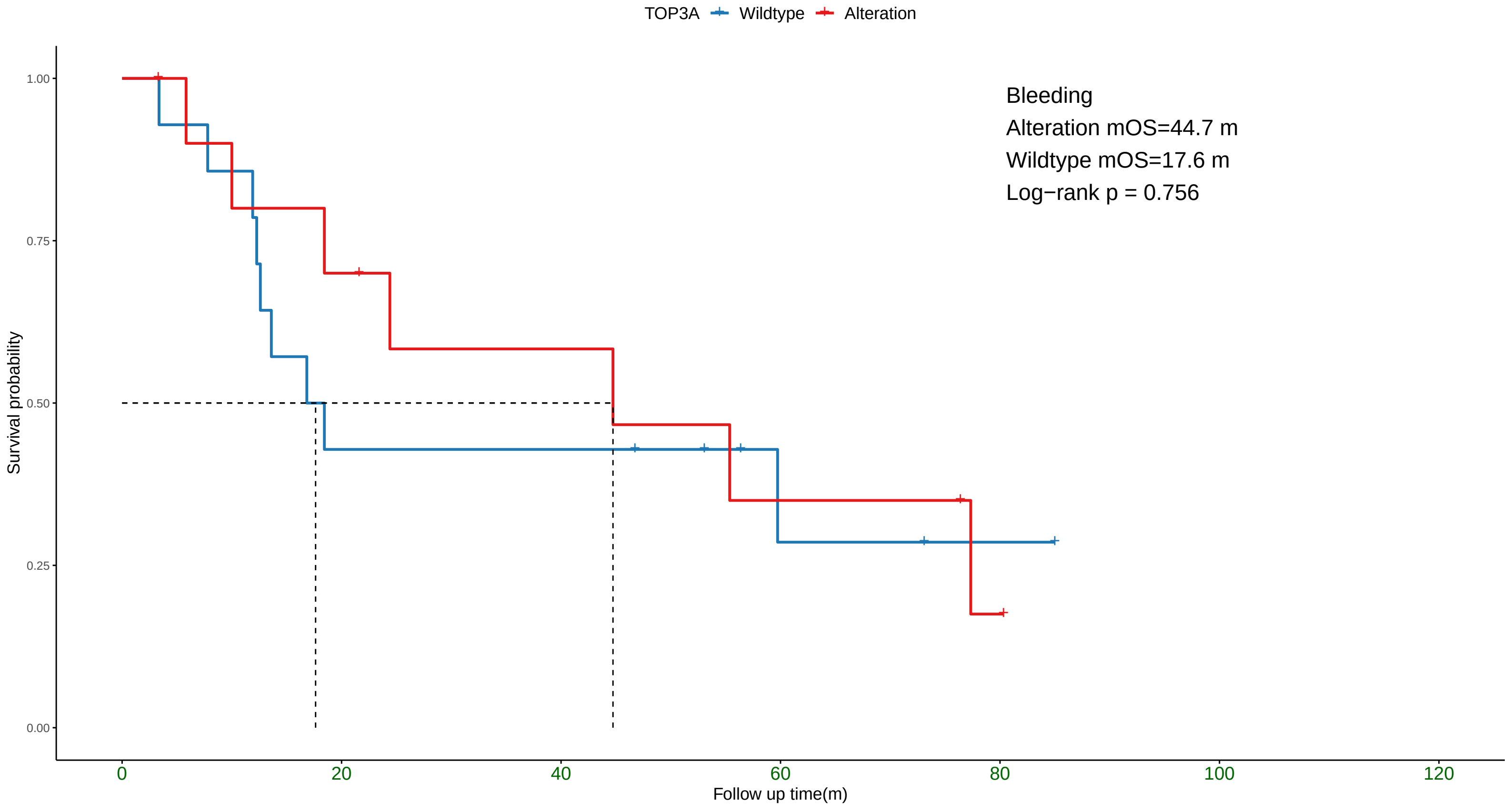
TOP3A Wildtype Alteration
Bleeding
Alteration mOS=44.7 m
Wildtype mOS=17.6 m
Log−rank p = 0.756
Survival probability
1.00
0.75
0.50
0.25
0.00
0
20
40
60
80
100
120
Follow up time(m)

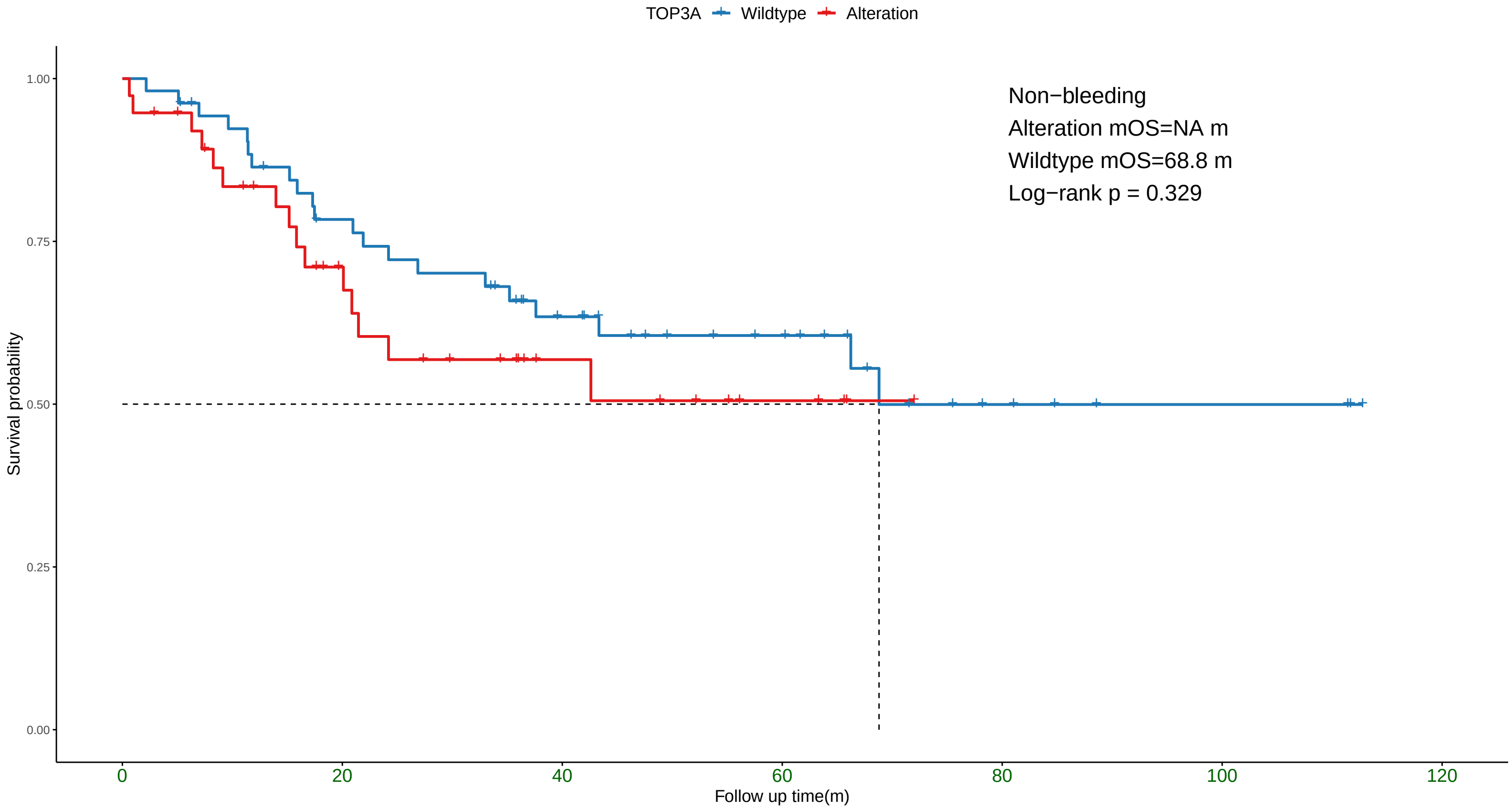
TOP3A
Wildtype
Alteration
Non−bleeding
Alteration mOS=NA m
Wildtype mOS=68.8 m
Log−rank p = 0.329
Survival probability
1.00
0.75
0.50
0.25
0.00
0
20
40
60
80
100
120
Follow up time(m)

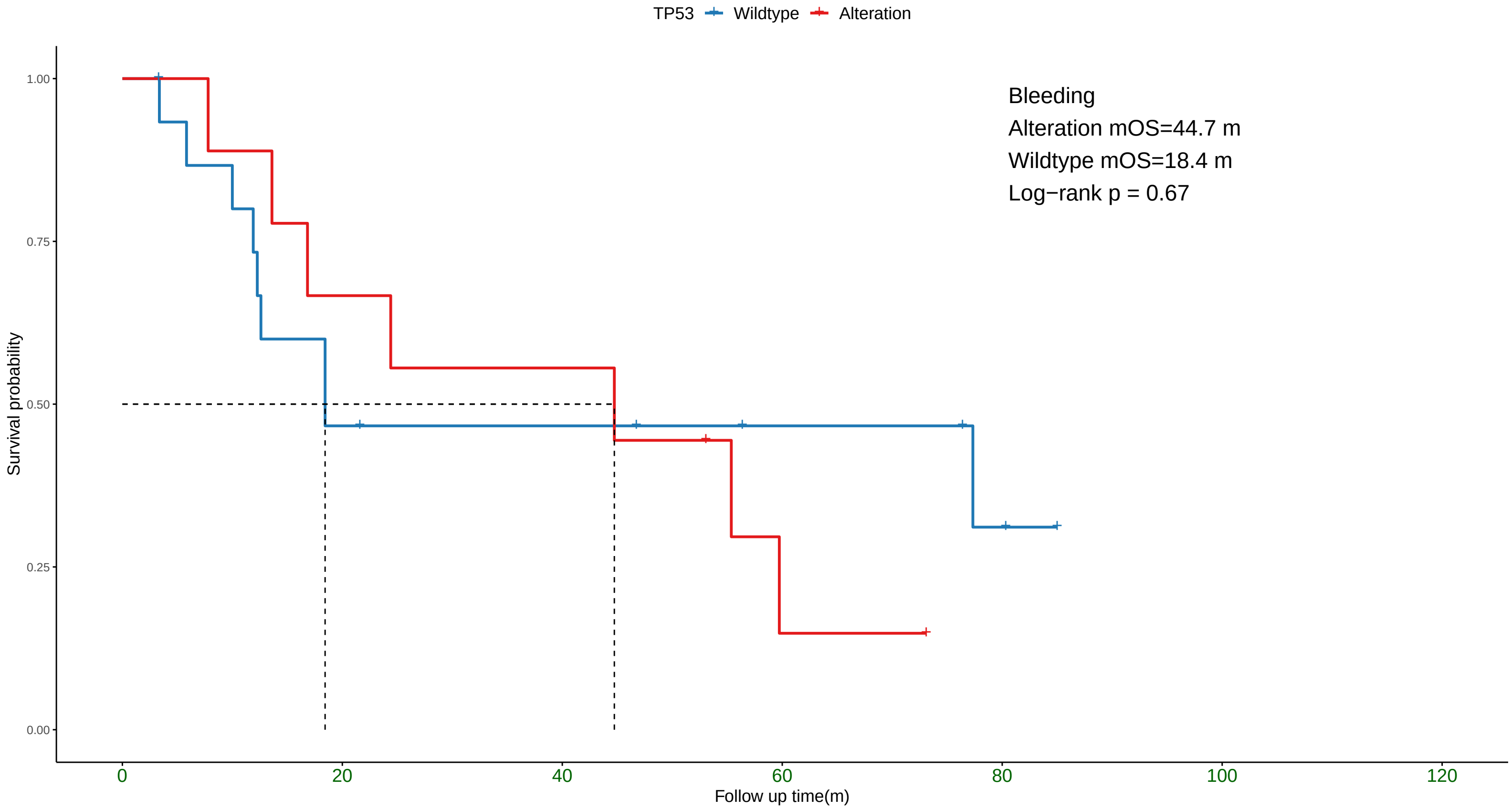
TP53
Wildtype
Alteration
Bleeding
Alteration mOS=44.7 m
Wildtype mOS=18.4 m
Log−rank p = 0.67
Survival probability
1.00
0.75
0.50
0.25
0.00
0
20
40
60
80
100
120
Follow up time(m)

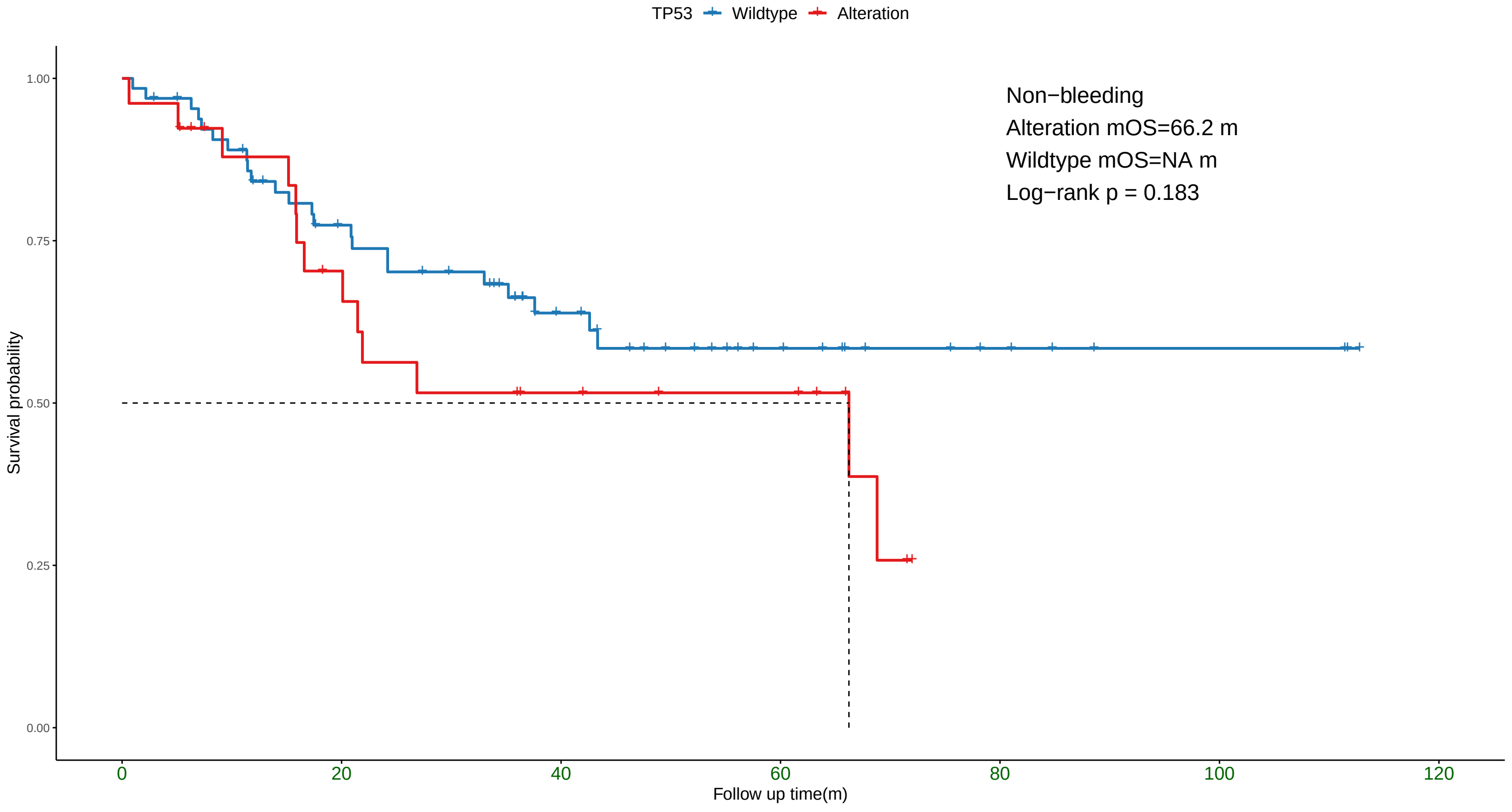
TP53
Wildtype
Alteration
Non−bleeding
Alteration mOS=66.2 m
Wildtype mOS=NA m
Log−rank p = 0.183
Survival probability
1.00
0.75
0.50
0.25
0.00
0
20
40
60
80
100
120
Follow up time(m)
